# Interactions in self-assembled microbial communities saturate with diversity

**DOI:** 10.1038/s41396-019-0356-5

**Published:** 2019-02-26

**Authors:** Xiaoqian Yu, Martin F. Polz, Eric J. Alm

**Affiliations:** 1 Department of Biology, Massachusetts Institute of Technology, Cambridge, MA USA; 2 Department of Civil and Environmental Engineering, Massachusetts Institute of Technology, Cambridge, MA USA; 3 Department of Biological Engineering, Massachusetts Institute of Technology, Cambridge, MA USA; 4 Center for Microbiome Informatics and Therapeutics, Massachusetts Institute of Technology, Cambridge, MA USA; 5 Broad Institute of MIT and Harvard, Cambridge, MA USA

**Keywords:** Biodiversity, Microbial ecology, Microbial ecology, Microbial biooceanography, Biodiversity

## Abstract

How the diversity of organisms competing for or sharing resources influences community function is an important question in ecology but has rarely been explored in natural microbial communities. These generally contain large numbers of species making it difficult to disentangle how the effects of different interactions scale with diversity. Here, we show that changing diversity affects measures of community function in relatively simple communities but that increasing richness beyond a threshold has little detectable effect. We generated self-assembled communities with a wide range of diversity by growth of cells from serially diluted seawater on brown algal leachate. We subsequently isolated the most abundant taxa from these communities via dilution-to-extinction in order to compare productivity functions of the entire community to those of individual taxa. To parse the effect of different types of organismal interactions, we defined relative total function (RTF) as an index for positive or negative effects of diversity on community function. Our analysis identified three overall regimes with increasing diversity. At low richness (<12 taxa), positive and negative effects of interactions were both weak, while at moderate richness (12–26 taxa), community resource uptake increased but the carbon use efficiency decreased. Finally, beyond 26 taxa, the effect of interactions on community function saturated and further diversity increases did not affect community function. Although more diverse communities had overall greater access to resources, on average individual taxa within these communities had lower resource availability and reduced carbon use efficiency. Our results thus suggest competition and complementation simultaneously increase with diversity but both saturate at a threshold.

## Introduction

Organismal diversity is recognized as a driver of ecological functions such as biomass production, resource turnover, and community stability. As the number of taxa increases so do positive and negative biotic interactions such as facilitation, niche complementation, and competition, which all modulate the efficiency of resource use. While niche complementation leads to optimization of resource use through avoidance of resource use overlap, competition—either directly for resources or indirectly by chemical interference—often negatively impacts ecosystem functions. A considerable number of models have been developed to determine how and why the relative strength of different interactions on community function changes with diversity [1–5]. Most models determine the net effect of interactions on communities by comparing the observed community function to that predicted from monoculture functions of community members. In many ecosystems, these models agree on a general increase of all types of interactions with diversity, as well as diverse communities being more productive due to the strong effect of niche complementation. It is, however, also possible for this relationship to be reversed [6]. Especially in microbial systems, it has been proposed that the negative effects of antagonism on community function are only outweighed by the positive effects of niche complementation if the microbes are functionally dissimilar and the resource environment is heterogeneous [2]. Recent work further shows that even when both conditions are satisfied, a negative relationship between diversity and biomass production can occur if interspecific competition is strong and hierarchical, such as in a highly antagonistic system of wood degrading fungi [4].

Because of their strong dependence on obtaining measurements of relevant functions of community members in monoculture, many community interaction models are most suitable for systems in which diversity can be varied by making a series of assemblages from well-characterized species. However, this approach is difficult for microbial ecosystems because they typically display high richness and often only a small portion of the total diversity can easily be isolated and grown in pure culture [7]. An alternative approach that removes species from natural communities via serial dilution can effectively generate communities of decreasing diversity while circumventing isolation, but in turn makes separating the effects of individuals on community function from that of interactions challenging [8–10]. As a result, artificial microbial assemblages have been used to experimentally study organismic interactions and these assemblages have been limited in richness and phylogenetic diversity. Importantly, it is not clear to what extent such artificial assemblages reflect naturally occurring interactions, which are often the result of long term evolutionary processes of co-occurring organisms. It therefore remains an open question how different types of biotic interactions contribute to community function across ecologically relevant ranges of diversity for microbes that have co-diversified in the wild.

Here, we address the problem of how microbial interactions and their effects on ecosystem functions change over a wide diversity spectrum by combining the dilution approach with isolation and pure culture studies. Dilution series of planktonic microbial communities are first allowed to self-assemble on seaweed extract as a realistic, complex environmental carbon substrate, which mimics an algal bloom in the coastal ocean [11, 12]. This process generated a series of replicate microbial communities with varying diversity and allowed measurement of biomass production and respiration as relevant community wide parameters. Following the measurements, each community was diluted to extinction to approach monoculture-level diversity, and the same production measures were obtained and compared to the community values. We extend relative yield total (RYT), a classical criterion for determining whether intercropping leads to higher crop yield than mono-cropping [13], to our multi-taxa system and generalize it to summable functions across community members as the relative total function (RTF). RTF can be further broken down to investigate how interactions affect community production through changes in total resource use or resource conversion efficiency. We find that with increasing diversity, total resource access increases due to niche complementation, but carbon use efficiency decreases due to competition, leading to a stronger increase in community respiration than biomass production. Our results show that the production of a natural community may be limited by both its potential to access resources and by the amount of niche overlap that allows members to coexist in nature.

## Results

### Experimental workflow

**Fig. 1:**
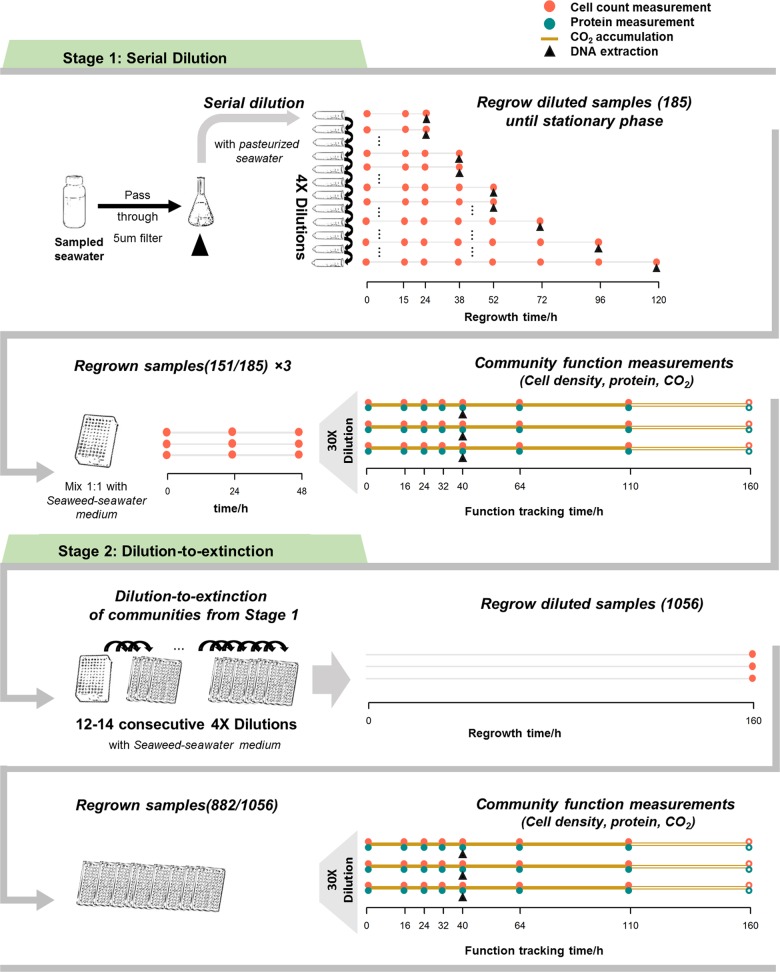
Schematic for community self-assembly and functioning measurements. In the first stage, a seawater sample is serially diluted and regrown in pasteurized  seawater to generate inocula of different diversities for community growth and function measurements in seaweed-seawater medium (SSM) over a period of 160 h. In the second stage, communities from the first stage are diluted to extinction to generate near monoculture samples that are subjected to the same growth and function measurements in SSM over a period of 160 h. Unfilled points/lines represent time points that were taken but are eventually omitted due to changes in physical properties of the cultures

To test the relationship between diversity and community function, we generated microbial communities of decreasing diversity by serially diluting the same seawater sample and subsequently tracking relevant measures of microbial functions during growth in seaweed-seawater medium (SSM, pasteurized seawater containing extract from the brown algae *Fucus*). Specifically, our approach consisted of two stages where the first generated communities of similar overall biomass but different richness for which community production and respiration were measured, and the second consisted of dilution-to-extinction of communities from the previous stage to generate monocultures for which the same community functions were measured as input to the RTF model (Fig. 1, Methods).

In the first stage, we generated 15 replicate samples from seawater passed through a 5 µm filter and serially diluted each sample in 12 steps of 4-fold dilution each to yield a total of 185 communities of differing cell numbers and diversity. To acclimate these communities to the conditions used for measurements of community function, each was regrown first in pasteurized filtered seawater without any additional nutrients, then diluted 1:1 into SSM and regrown for 48 h, and finally diluted 1:30 into SSM and grown for 160 h (Fig. 1). This final regrowth experiment was used to measure community functions at different diversity levels. In the second stage, the resultant communities were diluted to extinction to obtain low complexity communities (typically 1–10 taxa) and regrown in SSM for 160 h in order to determine the same functions under near monoculture conditions.

Biomass production was measured as the maximum of both cell numbers and per cell protein content that the communities reached within the first 110 h of observation, while respiration was estimated as the total CO_2_ production during this time. The combined measures of biomass production and respiration allow estimation of resource use efficiency. Finally, community composition was assessed by 16S rRNA amplicon sequencing immediately after the first diversity removal step and after each community reached early stationary phase in SSM. To estimate the total taxonomic richness, we first counted the number of taxa as clusters of identical 16S rRNA amplicon sequence variants (ASVs) [14, 15], and then inferred the number of ASVs that were uncounted due to limitations in sequencing depth [16]. Since observed and estimated taxonomic richness were highly similar in all cases (Fig. S1), we used direct ASV counts as the approximate taxonomic richness.

Of the total 185 possible communities from the first stage dilution series, 151 showed sufficient cell numbers after regrowth in pasteurized seawater to serve as inoculum into SSM. These communities ranged in taxonomic richness and cell densities from 5 to 350 and 1.1 × 10^4^ to 3 × 10^5^ cells/mL, respectively (Fig. 1 and S2a). As expected, more diluted communities contained fewer and more dissimilar sets of taxa (Fig. S2b, c). All communities experienced further loss of taxa during their regrowth in SSM (Fig. S3a, b) possibly due to environmental filtering, population bottlenecks during transfer, or competitive exclusion.

The second stagedilution-to-extinction experiment to create monocultures yielded 882 samples, most containing less than 10 taxa, with 275 almost completely dominated by a single taxon (>90% of reads belonging to one ASV). These monocultures represent 37 ASVs in 7 families (Table S1), and cover 75.8% of total reads for the communities grown in SSM from the first dilution, hence providing a good basis for comparison of community-level and single taxon measures of function.

### Relationships between diversity and community functions vary

**Fig. 2:**
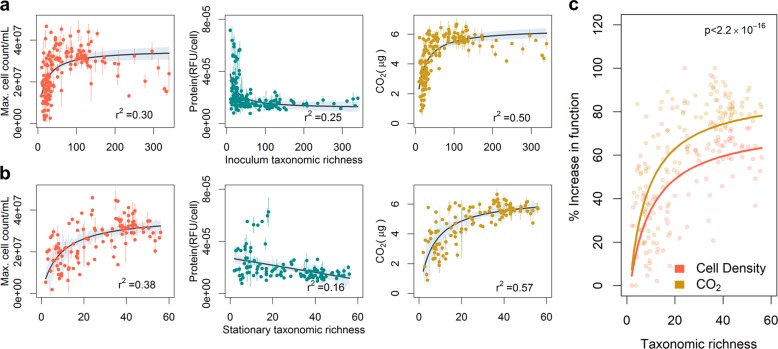
Diversity strongly impacts all measures of community function. Relationship of different measurements of community production (cell numbers and protein per cell) and respiration (CO_2_ production) with **a** inoculum taxonomic richness and **b** stationary phase taxonomic richness of communities. **c** Comparison of the hyperbolic fits for CO_2_ production and cell density scaled to 1 as maximum. Each dot represents one inoculum community; colored bars indicate the standard deviation of the measurement; black/colored lines indicate fits to an appropriate model selected between a linear, log-linear, and hyperbolic least squares fit with blue-gray regions around the line indicating the 95% confidence of the fit

We observed Michaelis-Menten-like hyperbolic relationships between taxonomic richness and cell density or CO_2_ accumulation, but negative relationships between taxonomic richness and protein production per cell, independent of whether taxonomic richness of the inoculum or resultant communities after growth in SSM was used (Fig. 2a, b). Normalizing both respiration (CO_2_ production) and cell density to the maximum measured difference between two communities, we found that respiration increased at a faster rate than cell density with increasing taxonomic richness (Fig. 2c, *p* < 2.2 × 10^−16^, paired *t*-test, two-tailed). Since the amount of protein per cell also decreased with diversity, this differential rate indicates that as the number of taxa in the communities rose, higher portions of assimilated carbon were released as CO_2_. Because such lowered yield in more complex communities could be due to individuals having intrinsically lower efficiencies in converting assimilated carbon into biomass or due to different taxa negatively affecting each other, we developed an indicator that allows differentiating between these two possibilities.

### Relative total function as an indicator for interaction effects on community function

**Fig. 3:**
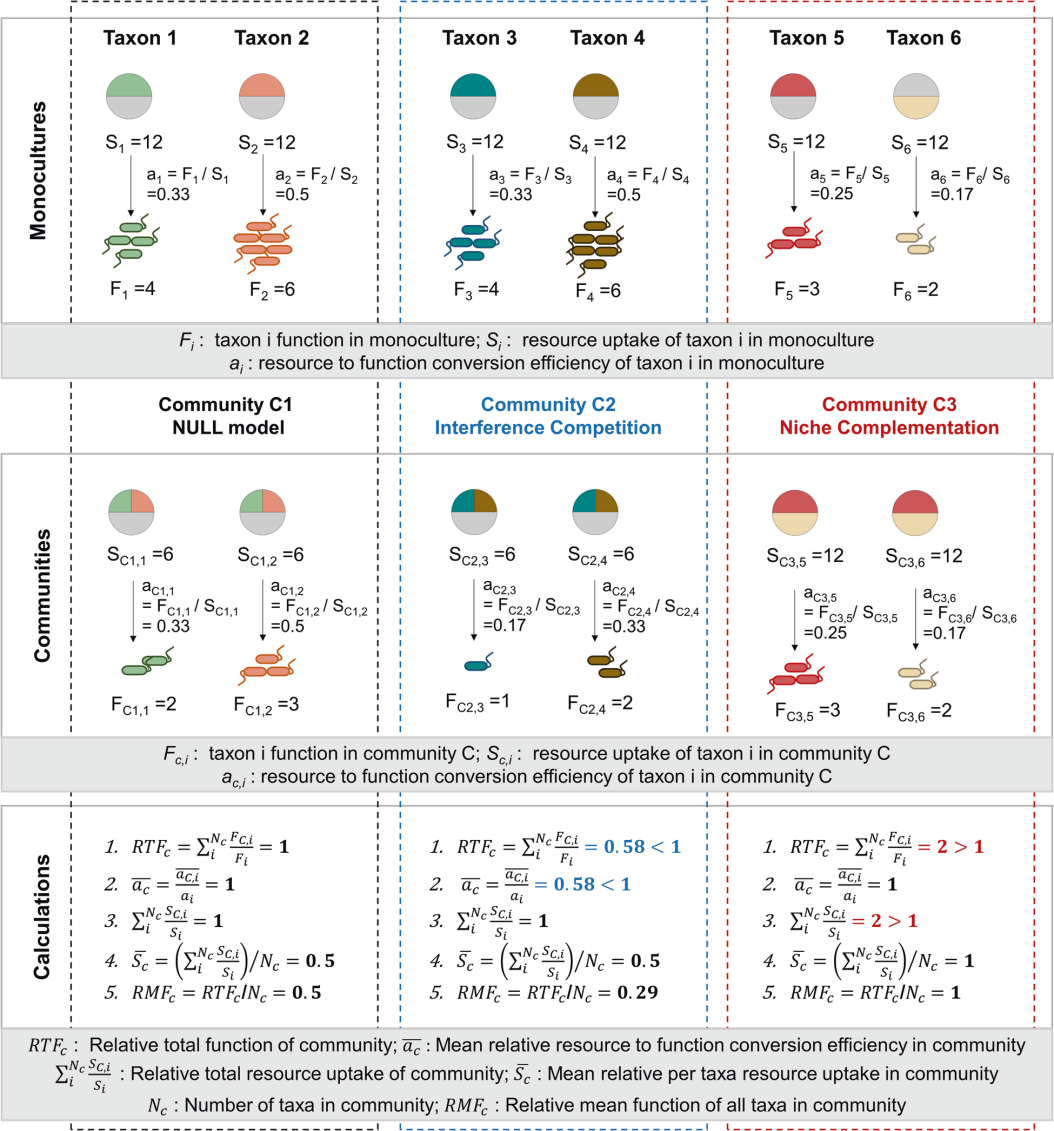
Graphical illustration of the *RTF*_C_ concept. The *RTF*_C_ allows determination of interaction effects on community function by summing the relative function of each taxon in the community compared to its monoculture. Functions suitable for this analysis are thus limited to those that can be summed across taxa, such as biomass or CO_2_ production. For illustration purposes, the function of interest used in this graph is the number of microbes. Community C1 represents a case of the null model (${\mathrm{RTF}}_{\mathrm{c}}=1$): taxa 1 and 2 are capable of utilizing the same resources when separately in monoculture (colored regions in circles; circles represent total resources available). When together in a community, the two taxa equally split the pool of resources that they are both capable of utilizing (relative total resource uptake $\mathop {\sum}\nolimits_i^{N_{\mathrm{C}}} {\frac{{S_{{\mathrm{C}},i}}}{{S_i}} = 1}$, mean relative per taxa resource uptake $\overline {S_{\mathrm{c}}} = 0.5$). However, the resource to function conversion efficiency of each taxon is not affected by the other (mean relative resource to function conversion efficiency $\overline {a_{\mathrm{c}}} = 1$). As a result, the output function (number of cells) is proportional to the resource allocation between the two taxa. In Community C2 (${\mathrm{RTF}}_{\mathrm{c}}\, < \, 1$), the two taxa 3 and 4 interfere with each other when sharing resources in a community, resulting in a mean relative resource to function conversion efficiency $\overline {a_{\mathrm{c}}} < 1$. Here, although resource uptake is exactly the same as in the null model, the output function is reduced. In Community C3 (${\mathrm{RTF}}_{\mathrm{c}}\, > \, 1$), the two taxa 5 and 6 perfectly complement each other in resource use (relative total resource uptake $\mathop {\sum}\nolimits_i^{N_{\mathrm{C}}} {\frac{{S_{{\mathrm{C}},i}}}{{S_i}} = 2}$, mean relative per taxa resource uptake $\overline {S_{\mathrm{c}}} = 1$), and do not affect each other’s resource conversion efficiencies $\left( {\overline {a_{\mathrm{c}}} = 1} \right)$. See also Fig. S4 for an extension case where complementation and competition cancel out to result in an *RTF*_C_ of 1 but should be distinguished from the null model

We established relative total function (RTF) as an indicator for inter-taxa relationship effects on community function by generalizing the concept of relative yield total (RYT) suggested by De Wit and Van den Bergh [13]. RYT is calculated as the sum of the relative yields of two species in a community compared to each of their monocultures. It is thus a measure of how resource use of one species is influenced by the other when the two occur together in a community, under the assumption that the resource to biomass conversion efficiency of each species is maintained between mono and community cultures so that biomass becomes an indirect readout for resource use. Generalizing two species to multi-species and yield to all community functions that are summable across species, we defined RTF as the summed relative function of each taxon in a community to their monocultures:

1 $${\mathrm{RTF}}_{\mathrm{C}} = \mathop {\sum}\nolimits_i^{N_{\mathrm{C}}} {\frac{{F_{{\mathrm{C}},i}}}{{F_i}}},$$

where *F*_C*,i*_ is the function of a taxon *i* in community C, *F*_*i*_ is the measured monoculture function of taxon *i*, *N*_C_ is the number of taxa in community C. Under the null model that community function is solely dependent on individual function, *RTF*_C_ equals 1, while an *RTF*_C_ larger than 1 indicates that biotic interactions increase community function, and an *RTF*_C_ smaller than 1 indicates a decrease (see Fig. 3, S4 for graphical illustrations).

Under most resource concentration regimes, the carrying capacity of a community can be seen as a linear function of resource uptake. Only when resource overabundance induces incomplete respiration of substrates, the assumption of linearity may be violated [17]. Since this is unlikely here due to limited resources being provided, we can further define for community functions analogous to carrying capacity:

2 $${\mathrm{RTF}}_{\mathrm{C}} = \mathop {\sum}\nolimits_i^{N_{\mathrm{C}}} {\frac{{a_{{\mathrm{C}},i}}}{{a_i}}\frac{{S_{{\mathrm{C}},i}}}{{S_i}} = ^{\ast} \overline {a_{\mathrm{c}}} } \mathop {\sum}\nolimits_i^{N_{\mathrm{C}}} {\frac{{S_{{\mathrm{C}},i}}}{{S_i}}},$$

_*__represents that the second step only holds when_ $\small \frac{{a_{{\mathrm{C}},i}}}{{a_i}}$_and_ $\small\frac{{S_{{\mathrm{C}},i}}}{{S_i}}$_do not co-vary._

where *a* is efficiency of resource conversion into the function of interest (i.e., the amount of resource converted into a specific community function, such as biomass or carbon dioxide production, divided by the total resource uptake), and *S* is the amount of resource uptake. Thus, there are two major ways that interactions could affect community function: by altering the total amount of resource uptake or the efficiency of resource conversion into the community function of interest. Under the null model, both the mean relative resource conversion efficiency ($\overline {a_{\mathrm{c}}}$), and the relative total resource uptake $\left( {\mathop {\sum}\nolimits_i^{N_{\mathrm{C}}} {\frac{{S_{{\mathrm{C}},i}}}{{S_i}}} } \right)$ would be 1. If either factor is larger or smaller than 1, it is positively or negatively affected by interactions between taxa (Fig. 3, S4).

While *RTF*_C_ compares the function of a community to the monoculture functions of its constituents, the function of individual taxa in a community can be compared to its monoculture using relative mean function *RMF*_C_. *RMF*_C_ is calculated by dividing *RTF*_C_ with the total number of taxa in the community (*N*_C_). A relative mean function (*RMF*_C_) larger than 1 indicates facilitation between the majority of community members. Furthermore, relative mean function (*RMF*_C_) can be seen as the product of mean relative per taxa resource uptake $\left( {\overline {S_{\mathrm{c}}} = \mathop {\sum}\nolimits_i^{N_{\mathrm{C}}} {\frac{{S_{{\mathrm{C}},i}}}{{S_i}}/N_{\mathrm{c}}} } \right)$ and mean relative resource conversion efficiency $\left( {\overline {a_{\mathrm{c}}} } \right)$. Thus, in ecological terms, the mean relative per taxa resource uptake $\left( {\overline {S_{\mathrm{c}}} } \right)$ is the ratio of the realized niche to the fundamental niche for the average community member (Fig. 3, S4).

We used the *RTF*_C_ indicator to evaluate how interactions between taxa impacted different community function measurements as diversity increased. Since *RTF*_C_ is only suitable for community functions that are summed across taxa, we applied it to three functions of interest: cell number, total protein production, and total respiration (as CO_2_ accumulation). However, calculation of *RTF*_C_ also requires knowing the function in community (*F*_C*,i*_) and in monoculture (*F*_*i*_) for every taxon in a community. We achieved this by defining communities that were at least 85% covered by the 37 taxa we were able to isolate in sufficient purity as “constitutable”, and assuming that the remaining 15% of taxa contribute to community function similar to the constitutable 85%. The 85% cutoff was chosen because it was the highest criterion that allowed constitutable communities to cover the full diversity spectrum of the stationary phase communities (Fig. S5). Using this cutoff, we identified 82 (35%) of the 235 total communities with good sequencing coverage to be constitutable for every community function. Subsequently, we calculated functions of the individual taxa in each community (*F*_C*,i*_) by multiplying the measured total community function *F*_C_ with relative abundances of each taxon from community composition measurements. This calculation estimates cells counts for each taxon (*F*_C*,i*(Cell count)_) in the community; however, for estimating protein and CO_2_ productions of individual taxa in the community (*F*_C*,i*(Protein)_ and *F*_C*,i*(CO2)_) the calculation is only valid if there are no large variations in the amount of protein per cell and CO_2_ production per cell across different taxa, which we found to be the case for the majority of taxa in our communities (see Methods for details).

### Interaction effects on community function differentially increase with diversity

**Fig. 4:**
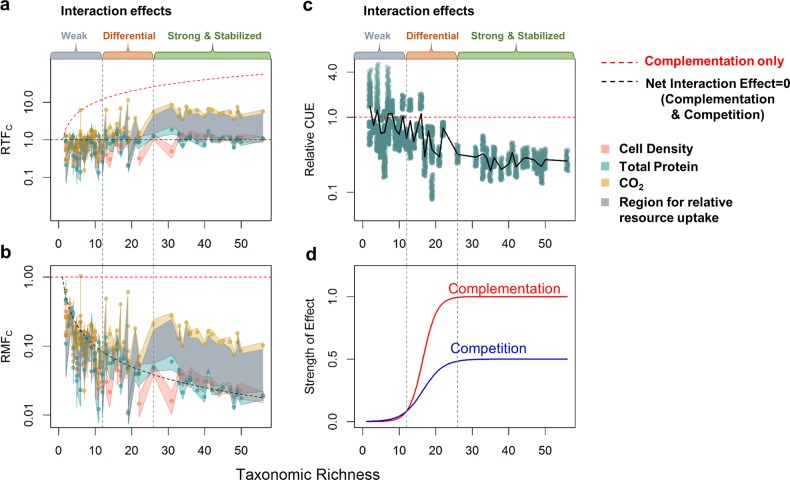
Relative community-to-monoculture functions indicate differential increases in niche complementation and competition with diversity. Relationship between diversity and **a** relative total biomass production, respiration, and resource uptake (*RTF*_C_ and $\mathop {\sum}\nolimits_i^{N_{\mathrm{C}}} {\frac{{S_{{\mathrm{C}},i}}}{{S_i}}}$) and **b** relative per taxa biomass production, respiration, and resource uptake (*RMF*_C_ and $\overline {S_{\mathrm{c}}}$) **c** estimated relative carbon use efficiency (CUE). In **a**, **b** each point represents one community at stationary phase (biological replicates were not averaged). In **c**, each point represents a combination of one relative total function (*RTF*_C_) ratio with a random value of CUE drawn from 0–0.6. The black line represents the mean of all combinations with the same taxonomic richness. Colored regions around the points indicate the standard deviation of the interaction effect, and the gray areas indicate the range where relative total and per taxa resource uptake is limited to at each taxonomic richness. The *y-*axes are in log scale for better resolution of points (see Fig. S6 for the same data in linear scale). **d** A hypothetical model for how the effects of niche complementation and competition on community function scale with taxonomic richness. Numbers on the *y*-axis are arbitrary

By tracking how different community functions relative to monoculture change with diversity, we found that as diversity increased, interactions lead to a stronger increase in community respiration (CO_2_ production) than community biomass production (cell count and total protein). At low richness (*N*_c_ ≤ 12), *RTF*_C_ was not significantly different from 1 for CO_2_ production, and slightly below 1 for total protein production and cell count, indicating that the effect of interactions on respiration was negligible but weakly negative for biomass production (Fig. 4a, S6a, *t*-test, two-tailed, *p*_CO2_ = 0.90, *p*_protein_ = 1.0 × 10^−6^, *p*_cell_ = 2.4 × 10^−10^). However, as taxonomic richness increased beyond 12, *RTF*_C(CO2)_ steadily rose above 1 until it eventually plateaued when a moderate richness level of 26 taxa was reached. Meanwhile, *RTF*_C(Protein)_ remained around 1 over the entire diversity range, and *RTF*_C(Cell)_ remained around 1 until 26 taxa, beyond which it stayed slightly above 1 (*t*-test, two-tailed, *p*_CO2,12<Nc ≤26_ = 6.4 × 10^−3^, *p*_CO2, Nc >26_ = 1.4 × 10^−11^, *p*_protein,12<Nc ≤26_ = 0.88, *p*_protein, Nc >26_ = 0.29, *p*_cell,12<Nc ≤26_ = 0.21, *p*_cell, Nc >26_ = 7.5 × 10^−3^). Thus, at moderately high diversity, interactions had a strong net positive effect on community respiration, but only a weakly positive effect on the production of cells, and no net effect on community protein production.

### Community resource uptake increases with diversity while individual taxa resource uptake decreases

Since the relative community CO_2_ production increased significantly with diversity, and relative community biomass production also exhibited a weak increase, the relative resource uptake of the community must also increase with diversity. Detailing this under the *RTF*_C_ framework, since each community has a single relative total resource uptake value $\left( {\mathop {\sum}\nolimits_i^{N_{\mathrm{C}}} {\frac{{S_{{\mathrm{C}},i}}}{{S_i}}} } \right)$, and the conversion efficiencies for CO_2_ and biomass accumulation have to change in opposite directions (i.e., they cannot simultaneously increase or decrease), the relative total resource uptake of a community must fall between *RTF*_C(CO2)_ and *RTF*_C(Biomass)_. Taking total protein production as the measurement for biomass, we found that the region between *RTF*_C(CO2)_ and *RTF*_C(Protein)_ moved from around 1 to higher than 1 as diversity increased (Fig. 4a, S6a). This indicates that biotic interactions with positive effects on resource uptake, such as niche complementation, are more prevalent in more diverse communities.

However, individual taxa in more diverse communities on average take in fewer resources, i.e., they have narrower realized niches relative to their fundamental niches in monoculture. This is supported by the gradual decrease of the mean relative per taxa resource uptake $\left( {\overline {S_{\mathrm{c}}} } \right)$, whose boundaries are defined by relative mean functions *RMF*_C(CO2)_ and *RMF*_C(Protein)_, with diversity (Fig. 4b, S6b). Also, since all *RMF*_C_s never exceeded 1, communities where most community members facilitate each other are rare. In fact, only a very small fraction of taxa produced more biomass or CO_2_ in communities than in monocultures (Fig. S7), indicating that each taxon always has more competitors than facilitators, and the frequency of mutualistic interactions among different taxa is lower than negative interactions.

### Competition in more diverse communities reduces carbon use efficiency (CUE)

Since relative community CO_2_ production increased faster with diversity than relative community biomass production, the relative carbon to biomass conversion efficiency (often known as carbon use efficiency, CUE) decreased, possibly as a result of stronger competition. Because the ratio of *RTF*_C(CO2)_ to *RTF*_C(Biomass)_ should scale positively with both the expected CUE from monocultures and how much this CUE changes upon introduction of the organism into a community, we estimated the range of the relative-to-expected CUE from the *RTF*_C(CO2)_ to *RTF*_C(Protein)_ ratios and limiting the expected CUE to the thermodynamic limits of 0–0.6 [[18], see methods for details]. We found that as taxonomic richness increased, interactions had increasingly negative effects on the relative CUE of the community, but the effect leveled off at moderate taxonomic diversity (Fig. 4c, S6c). Furthermore, since at higher richness *RTF*_C(Protein)_ was lower than *RTF*_C(Cell)_ (*N*_C_ > 26, *p* = 0.06, paired Wilcoxon rank sum test, one-tailed), the relative CUE decrease is a joint effect of both smaller cell size and lower cell number. This is likely because microbes in more diverse communities are under stronger competition for resources, as evidenced by their narrower relative realized niches. They either have to grow faster to directly compete with other microbes for preferred resources or avoid competition by using more recalcitrant and less preferred resources. Because growth rate generally scales positively with cell size [19–21], it appears more likely that our observation of smaller cells in more diverse communities indicates reduced growth rate and partitioning of a larger portion of carbon taken up toward respiration due to the lower energy yield of more recalcitrant substrates.

**Fig. 5:**
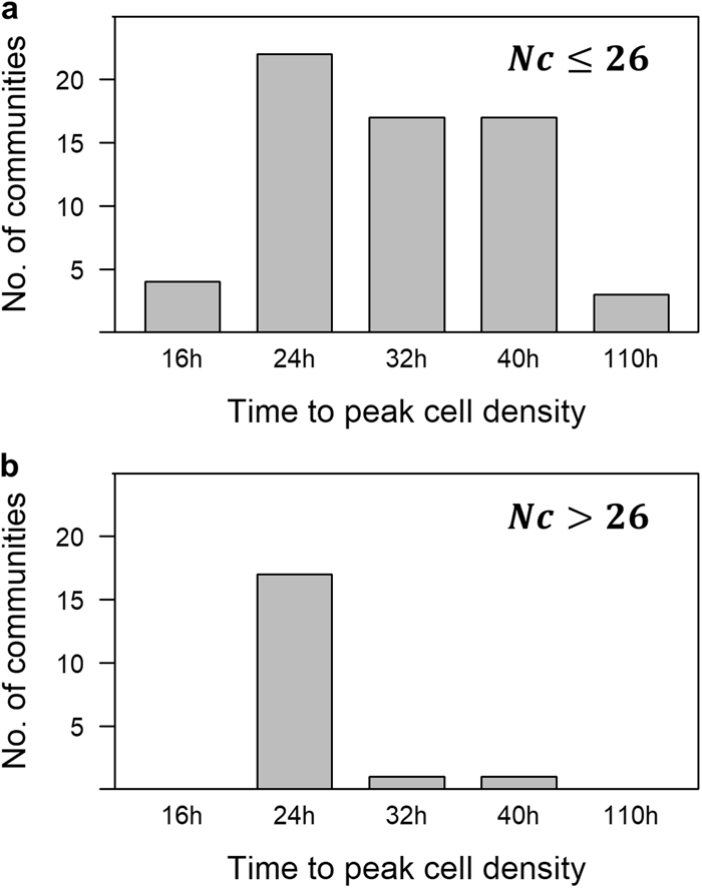
More diverse communities reach stationary phase earlier. Distributions of time for communities to reach peak cell density in communities with **a** less than 26 taxa (*N*_c_ ≤ 26), and **b** more than 26 taxa (*N*_c_ > 26)

However, it is possible that the “faster growth” strategy dominates initial periods of growth and the “recalcitrant resource” strategy takes over in later growth phases. We observed that more diverse communities had, at least initially, faster growth rates. An overwhelming majority of communities with more than 26 taxa reached maximum cell density by 24 h of growth, while those with less than 26 taxa had equal probability of growing to maximum at 24, 32, or 40 h (Fig. 5). Hence, our data suggest that diverse communities had a high probability of containing members specialized for substrates that allow initial rapid growth. Conversely, communities with lower diversity only occasionally contained such potentially fast growers, which can bloom under conducive conditions but otherwise occur at low concentration in natural communities, explaining the broader distribution of times to maximum cell numbers. Although this does not provide direct evidence for individual taxa growing faster in more diverse communities, it may indicate that it could be advantageous for taxa in more diverse communities to regulate itself for faster growth compared to monocultures. We would then expect relatively lower CUE but larger cells to accumulate early in the experiment [22, 23]. Over the full observation period, however, more diverse communities on average had smaller cell size, possibly because after reaching peak density, the initial population was replaced by other taxa or switched into a metabolism that utilizes substrates that are more recalcitrant and less energy efficient.

### Effects of interactions on community function increase logistically with diversity

Although cell density and CO_2_ both exhibit Michaelis-Menten-like hyperbolic relationships with diversity, both competition and complementation have logistically increasing effects on community function as diversity increases. By estimating CUE through the size of the difference between *RTF*_C(CO2)_ and *RTF*_C(Protein)_ and total niche occupation through their relative position to 1, we found that at low diversity (*N*_C_ ≤ 12), the effects of both competition and niche complementation were weak, with the negative effects of competition narrowly exceeding benefits of niche complementation. As diversity increased beyond this level, the positive effect of niche space expansion gradually exceeded that of the negative effect of competition until they both stabilized at moderately high diversity (*N*_c_ > 26). This indicates that while niche complementation is probably limited by the total amount of resources available, competition also has plateaued (Fig. 4d). Overall, these patterns translate to a logistic model of growth as diversity increases for impacts on community function by either competition or complementation.

### Specific taxa effects on community function

Since it is often assumed that organisms that are more phylogenetically distant are also more metabolically distinct, and taxonomic richness only partially explained the variance of community function (Fig. 2a, b), we asked if phylogenetic diversity of the communities affected community cell production or respiration through niche complementation. We found that when compared to species richness, neither of the two most common measurements for phylogenetic distance, the abundance weighted mean pairwise distance (MPD) or the abundance weighted mean nearest taxon distance (MNTD), was better in explaining the variance of community functions (see Table S2 for comparison between all models/diversity metrics).

We then checked the possibility of specific “key” taxa affecting community function, i.e., whether one taxon could alter community function without altering MPD or MNTD. We screened for these taxa by looking for ASVs whose relative abundance significantly correlated (Kendall rank correlation, *q* < 0.05 after FDR correction) with community function within sliding windows of taxonomic richness (window widths ranging from 2 to 10) (see Table S3 for all significant correlations). In most cases, each identified taxon was specific to a certain range of taxonomic richness (usually of size 10–15), with the direction of correlation highly consistent among windows within the range. Also, correlations between taxa and CO_2_ were few compared to those for cell density and total protein production, indicating that biomass production is more sensitive to taxonomic alterations than respiration. We identified five ASVs that exemplify how diversity may affect the outcome of microbial interactions and consequently community function.

Taxonomic richness of the inoculum strongly influenced dominance patterns in communities and thereby had significant effects on community function. For example, one taxon (ASV3) belonging to the genus *Alteromonas* was found to positively correlate with CO_2_ production but only in communities with less than 10 taxa. However, ASV3 did not show strong dominance in communities with higher inoculum diversity. This suggests that there were other, more rare taxa that were able to compete with ASV3 and demonstrates the importance of priority effects in colonization of small resource patches in the environment [24].

Despite having generally more niche overlap for resources, communities with high diversity can still benefit from having taxa that use the less common resources. ASV30, identified as *Wenyingzhuangia*, positively correlated with total protein production in communities with high taxonomic richness. Members in the *Wenyingzhuangia* genus are among the few organisms known to degrade the highly sulfated and recalcitrant sugar Fucoidan [25], estimated to be 4–10% of the dry weight of *Fucus* [26]. ASV30 may thus have positively affected community production by occupying a niche inaccessible to most other taxa, and might even have acted as a pioneer taxon by degrading the recalcitrant Fucoidan and converting it into more easily usable substrates.

Other taxa were found to have effects on community production likely through facilitation and predation. Several ASVs belonging to the genus *Sulfitobacter* were consistently found to be positively correlated with community production across different ranges of community richness and production measurements. Members of this genus are found as stable associates with algae [27], and possess sulfite-oxidation and aromatic compound degradation abilities [28]. Given that *Fucus* contains large amounts of aromatic compounds, *Sulfitobacter* could have positively affected the community production through degradation of aromatic compounds, which could otherwise impede the growth of other bacteria. We also found a negative correlation between ASV66, a putative predatory bacterium from the genus *Halobacteriovorax* [29], and community cell density at high taxonomic richness, indicating that predatory behavior may also play a role in determining community production.

## Discussion

In this study, by growing serially diluted seawater on brown algal leachate to generate self-assembled communities that span a wide range of diversity, we examined how diversity could impact community function through various interactions. Consistent with observations from artificially assembled systems, we found communities with greater diversity to have greater resource uptake due to niche complementation. However, the expansion of resource use came at the cost of increased competition driven by niche overlap and decreased the carbon use efficiency of the communities.

Compared to assembling “bottom-up” communities from known isolates, our method has both advantages and caveats. Since our communities self-assemble, our workflow is inevitably less well controlled compared to artificial assembly experiments and has more challenges regarding detailed characterization of individual traits and interactions. For example, unlike isolate assemblages that could be mixed to the exact same cell density, our inoculum communities only had cell densities on the same order of magnitude. Thus, checking that relationships between diversity and community function remain significant after considering the effect of initial cell density via multi-variable regression is necessary (Table S4). Also, since our method was based on 16S rRNA gene amplicon sequencing, we were only able to distinguish bacteria that had at least one nucleotide difference in the 16S rRNA V4 region. Different strains of bacteria that show genetic variability beyond the 16S rRNA region and have different productivities would be collapsed as one taxon in our analyses.

Despite these limitations, our method has the advantage of providing a more accurate recapitulation of how natural microbial communities assemble under defined environmental conditions and a more precise measurement of how community diversity alters different measurements of community function. Using organisms that have shared and adapted to a common habitat is especially important in light of studies that show evolutionary history can alter resource use patterns of taxa and can have a strong influence on how community functions are affected by biodiversity [30, 31]. Therefore, communities artificially assembled from strains that do not necessarily have a common history may provide a less realistic picture than provided by using a “top-down” approach of directly allowing natural communities to self-assemble to different diversities.

We thus argue that a “top-down” approach provides a better window into how diversity affects community function in nature. In previous work, this self-assembly approach has proven powerful in evaluating how and when various biogeochemical functions are affected by loss of diversity in a number of ecosystems such as fresh water lakes and top soil [8–10]. However, it was not possible to provide more detailed exploration of the underlying mechanisms for functional loss [8–10]. We addressed this problem by adding a dilution-to-extinction step to the traditional dilution approach, effectively allowing us to study how community function is mediated via interactions across a diversity gradient by comparing community function to monoculture functions via the *RTF*_C_ index.

Data generated from our method is also suitable for most other models that evaluate the net effect of interactions on communities by comparing the observed community function to that predicted from monoculture functions of community members. While our null model places a strong emphasis on comparing the uptake and utilization efficiency of resources between monocultures and communities, many other null models assume the performance of an individual in a community is directly proportional to its initial relative abundance, i.e., all individuals have the same growth rate. For example, in the Loreau and Hector model [3], the deviation between the expected and observed community function is called the net biodiversity effect, which can be further broken down into a part due to growth rate differences between individuals (“selection effect”) and a part due to resource complementation and competition (“complementarity effect”, which is linearly related to the *RTF*_C_ index). Applying such a null model towards our system shows that the net biodiversity effect is mostly driven by the complementarity effect (Fig. S9). Thus, since our major purpose was to elucidate how interactions influence community functions through different resource use patterns across a diversity gradient, we chose to use our current null model that allows easy breakdown of interaction effects on functions into that on resource use and resource to function conversion efficiency.

In our system, we found a large decrease in CUE with diversity, possibly due to increased competition for resources. Although decrease of CUE with increasing diversity due to interactions has been documented previously, our data suggest an unusually large effect. For example, in an artificially assembled system of saprophytic basidiomycete fungi, it was found that interactions in multispecies communities can decrease CUE by up to 25%, a stronger reduction than induced by many abiotic factors such as temperature increase [32]. However, it is estimated that most of our communities at moderately high diversity have a 60–80% decrease in CUE due to interactions. It is unlikely that this large CUE decrease in our system is due to antagonism as suggested by Maynard et al. [32], since instead of selecting a system (wood decaying fungi) and conditions that favor antagonism, we are mimicking a free-living and dilute marine environment. Although antagonistic potential has been demonstrated in marine bacteria [33, 34], it is more likely that the large interaction effect on CUE in our system results from resource competition, which Maynard et al. made efforts to minimize by having excess supply of both carbon and nitrogen [32]. By contrast, with seaweed extract being mostly organic matter, our system is likely limited by nitrogen or phosphate. Thus, with the narrower realized niches we observe as diversity increases, resource competition is likely the major driving force for the CUE decrease in our communities.

Furthermore, in our system, the impacts of both competition and complementation on community function logistically increased as diversity increased. This is probably a result of algal exudates being overall ubiquitous in the coastal ocean, but consisting of substrates that have different numbers of bacterial consumers. Certain substrates may only be utilized by a portion of bacteria in the environment, and the chance of getting such bacteria in a community would be equally small among communities with different richness when diversity is low (*N*_c_ ≤ 12). Only when there was a sufficient number of taxa in the community did adding in new taxa actually expand the resource use profile, and this expansion quickly become limited by the amount and types of resources available. Moreover, the speed of the resource use expansion was slower than that of the increase in taxa, thus niche overlap also increased with diversity. However, niche overlap also only started to translate to negative effects on community production when there was a sufficient amount of taxa in the community, possibly because only when there are enough competitors around there would be a need for employing a fast growth, low efficiency strategy.

The saturation of competition in our system at moderately high diversity is possibly due to a combined effect of organisms co-diversifying over long periods of time under the specific environmental conditions of the coastal ocean. The limited amount of competition we observe is consistent with theoretical predictions that environmental fluctuation place upper bounds on how much overlap there can be between niches for species in the community to stably coexist [35]. Indeed, the communities assembled here were drawn from the highly fluctuating coastal environment, and given that niche overlap is the result of organisms having sets of redundant functional genes, this might indicate that there is a limited amount of functional similarity between algal degrading bacteria due to the frequency of environmental fluctuation.

In conclusion, using self-assembled microbial communities directly derived from a costal ocean seawater sample, we found that complementation and competition for resources both increase with diversity but reach a threshold at a moderate number of taxa, beyond which no further effect of these interactions is evident. The simultaneous increase of complementation and competition with diversity generates trade-offs between the range and efficiency of resource use. Although the exact diversity thresholds where the impact of competition and complementation on community function saturates in our system are specific to our experimental setup, such a threshold should exist in many natural habitats: while it is expensive for organisms to maintain pathways for resources they rarely encounter, competing for more common resources can also require costly strategies and puts the organism at higher risk of competitive exclusion. Therefore, limits on interactions between wild populations of bacteria are likely a result of them maintaining a tremendous amount of diversity in fluctuating environments over long periods of time.

## Methods

### Media preparation

To prepare pasteurized seawater as a media for bacterial growth and as the solvent for making seaweed-seawater media (SSM), a total of 8 L of costal surface seawater was collected from a sampling site near Northeastern University’s Marine Science Center (Canoe Beach, Nahant, MA, USA; N 42° 25′ 11.6″,W 70° 54′ 24.8″), on Nov 12th, 2016. The water temperature at the time of sample collection was 12.0 °C. Seawater was pasteurized as described in Takemura et al. [11]. Briefly, the seawater sample was divided into 2 L bottles, heated to temperatures between 78 and 82 °C in water baths and maintained at the temperature for 1 h. Each bottle was pasteurized twice with at least 48 h intervals between the pasteurization events. The pasteurized seawater was then combined and filtered through 0.22 µm filters to remove any large size particles.

Stock solution for making the SSM was made from *Fucus vesiculosus* collected from the rocky shorelines of Canoe Beach on July 12th, 2015. The *Fucus* was washed, sun dried and grinded using a blender (Waring). Four grams of the ground *Fucus* was mixed with 100 mL of pasteurized seawater, and stirred at 150 rpm for 2 h at room temperature. The mixture was then passed through 20 µm Steriflip filters (Millipore), diluted 4-fold with pasteurized seawater, passed through a 0.22 µm filter (Corning, pre-washed three times with MilliQ water), and pasteurized again. The seaweed media extract stock solution was stored in the dark at room temperature till time of use, when it was diluted 10-fold in pasteurized seawater to make SSM. Lyophilizing 10 mL of the 10X stock solution resulted in 22 mg of dried material; thus the concentration of dissolved organic matter in the SSM was approximately 0.022% (w/v).

### Sample collection and experimental design

In order to generate the inoculum communities, a costal surface seawater sample was collected from a sampling site near Northeastern University’s Marine Science Center (Nahant, MA, USA; N 42° 25′ 11.6″,W 70° 54′ 24.8″), on Nov 18th, 2016. The water temperature at the time of sample collection was 11.5 °C.

The collected seawater was filtered through a 5 µm filter (Whatman) to remove particulates and larger eukaryotes and was estimated to have a microbial concentration of 3 × 10^5^ cells/mL via Fluorescence-activated cell sorting (FACS) using absolute count beads. Thus, an initial “undiluted” seawater community was defined as 30 mL of the filtrate, containing approximately 10^6^ cells in total. Fifteen “undiluted” seawater communities were 4-fold serial diluted with pasteurized seawater, generating 15 communities (30 mL) for each dilution level (4^2^X to 4^11^X). An additional 5 and 15 sub-communities were generated for the two highest dilution levels (4^10^X and 4^11^X).

All diluted communities were placed in 50 mL Falcon tubes and rotated end-over-end at 6.5 rotations per minute in the dark. Bacterial growth in each tube was repeatedly sampled over time using FACS until they were determined to have reached stationary phase (less than 20% increase in cell count between two consecutive time points following time points with over 20% growth in between), when they were destructively sampled for DNA extraction and used as inoculate into SSM. Time points for sampling were 0, 15, 24, 38, 52, 72, 96, 120, and 360 h.

For each community, three replicates of 0.3 mL each were used to inoculate an equal volume of 2X SSM. The communities were allowed to grow for 48 h in 96 deep well plates on a floor shaker at 300 rpm before being diluted 1/30 into 580 µL of SSM. The re-inoculated cultures were grown in MicroResp Systems (James Hutton Ltd, Aberdeen, UK) on a floor shaker at 300 rpm, and their community functions tracked as cell count, total protein, and CO_2_ production at time points 0, 16, 24, 32, 40, 64, 110, and 160 h. The 160 h data point was eventually omitted due to possible changes in the physical properties of the culture interfering with the FACS measurements.

At the end of the tracking period, communities similar in initial dilution levels and 160 h cell count were combined. The combined communities were diluted in SSM according to their cell densities so that on average each diluted community would contain 1 cell per 200 µL of culture. Each combined community had 18–24 corresponding diluted communities. These diluted communities were allowed to grow for 7 days in flat-bottom 96-well plates before they were screened for positive growth using FACS.

Communities that scored positive for growth were diluted 1/30 into 580 µL of SSM, and again grown in MicroResp Systems on a floor shaker at 300 rpm, with their community functions tracked as cell count, total protein, and CO_2_ production at time points 0, 16, 24, 32, 40, 64, 110, and 160 h. The 160 h data point was eventually omitted due to possible changes in the physical properties of the culture interfering with the FACS measurements.

### Community growth and function measurements

For tracking growth of the diluted communities in pasteurized seawater for generating inoculum communities, at each time point 100 µL subsamples of the communities were obtained and fixed 1:1 with 0.8% Formaldehyde + 0.5 µg/mL 4′,6-Diamidino-2-Phenylindole (DAPI, Sigma). During subsequent growth of the inoculum communities in SSM for community function measurements, at each time point 20 µL of each culture was fixed 1:10 with 0.8% Paraformaldehyde (BeanTown Chemical), and mixed 1:1 with staining media (1:5000 SYPRO red + 0.02% SDS + 1 µg/mL DAPI) for 30 min in the dark at room temperature [36], with some modifications].

All FACS measurements were performed using a BD LSRFortessa Flow Cytometer with a high throughput sampler. Bacterial cells were gated by forward and side scatter as well as the intensity of DAPI staining under a 500 V laser with activation wavelength of 405 nm, and collected through a 450/50 nm band-pass filter. Signal points that were between 20-4 × 10^4^ FSC-H, 40-4 × 10^4^ SSC-H and showed more than 200U blue fluorescence were counted as bacteria (Fig. S10). These gates were based on the following criteria: (a) The SSC-H threshold represents the lowest values above the region that accumulated 1000 events/min when running a blank sample, while the gated region above the threshold accumulated less than 100 events/min. (b) Events in the gated region shift to higher FSC-H values when the voltage of FSC is dialed up, while events below the FSC-H threshold do not move and are likely machine noise. (c) A population of cells clearly distinctive from background noise appeared in the region bound by SSC-H and FSC-H for different isolate cultures as well as environmental samples stained with DAPI, while no events appeared for blank samples or unstained cells (To reduce background noise, acquisition was triggered by blue fluorescence signal). The gating protocols were validated by comparing the CFU/ml of a *Vibrionaceae* strain (grown with twice the substrate concentration as SSM) to cell density calculated from the number of gated events (Fig. S10f). The CFUs were counted in triplicate by plating 100 µL of serial dilutions on Marine Broth 2216 plates (BD Difco). Fluorescence for the SYPRO red stain was determined with a 561 nm excitation laser (630 V) and 610/20 band-pass filter.

For consistency, cell counts for both community function measurements and growth tracking were determined by the number of DAPI positive events in the selected gated region for bacteria. Since 99.0 ± 3.6% DAPI positive cells in all non-blank samples also stained positive for SYPRO red, the protein per cell was determined by measuring the mean SYPRO red fluorescence for all DAPI positive events. Total protein was calculated via multiplying protein per cell by cell counts. For each 96-well plate of samples, cell counts were normalized to 3–6 standards of CountBright absolute count beads (Thermo Fisher) at 990,000 beads/mL, and total protein was normalized to 3–6 wells that contained a fixed standard marine bacteria mixture containing approximately 1:1 *Vibrionaceae and Halomonadaceae*.

CO_2_ production of communities was calculated from reading indicator plates in the MicroResp System on a plate reader at *λ* = 572 nm [37]. In the MicroResp system, all target communities were placed in deep 96-well blocks and connected to a top indicator plate using a seal. The seal insulated wells from each other but allowed the indicator plate to reflect the % of CO_2_ accumulated in the headspace of each well. The MicroResp indicator plates were made and calibrated according to the manufacturer’s instructions. The relationship (*R*^2^ = 0.996) between %CO_2_ in headspace and absorbance was (%CO_2_) = 0.1648/ (Δ_572_−0.2457)−0.2301, with Δ_572_ being the difference in A_572_ between the start and end of CO_2_ production time. Compared to the manufacturer’s instructions, we increased the number of measurements at low CO_2_ concentration, and were able to detect CO_2_ at levels as low as 0.025% (v/v). The average precision for all points on the standard curve was ±4% of each measurement (Fig. S11). The rate of CO_2_ production per volume of culture was calculated from %CO_2_ for each sampling interval, and a normalized total CO_2_ production of communities was calculated by summing CO_2_ production rate × average time × average community volume for each sampling interval. The effect of atmospheric CO_2_ was removed by normalizing the CO_2_ production values to the average of the blank wells.

Measurements eventually used as community functions were: maximum cell density the community reached within 110 h, maximum total protein production and protein per cell within 110 h, as well as total normalized CO_2_ production within 110 h. To account for cells clumping in later time points, the maximum protein per cell was calculated from maximum total protein production/maximum cell density, instead of directly comparing protein per cell measurements between time points.

### DNA extraction

In order to determine community composition for the inoculum communities, 30 mL of the inoculum communities were pushed through Swinnex Filter holders (13 mm, Millipore) containing 13 mm 0.22 µm filters (autoclaved, Durapore membrane PVDF, Millipore) connected to Luer-Lok syringes (BD). Filter paper was removed from the holder, cut into 4–6 smaller pieces, submerged in 125 µL QE buffer with 1% Ready-Lyse Lysozyme (Epicenter, Quick Extract Kit) in eppendorf tubes, and shook at 400 rpm overnight at room temperature. The tubes were spun down at 1700 rpm for 5 min the second day and the supernatant was stored at −20 °C till future use.

The composition for communities growing in SSM were determined at early stationary phase. For each community, 200 µL of sample was taken and filtered through MultiScreen HTS GV filter plates (0.22 µm, sterile, PVDF membrane, Millipore) by spinning the plates for 5 min at 3000 rpm. Each well was incubated overnight in 100 µL QE buffer with 1% Ready-Lyse Lysozyme (Epicenter) on a tabletop shaker at 400 rpm. DNA extract was collected by spinning the plates for 5 min at 3000 rpm and obtaining the flow through.

### Library prep, sequencing, and quality control

16S rRNA gene amplicon libraries (V4 hypervariable region, U515-E786) were prepared according to the method described by Illumina 16S metagenomic library preparation with some slight modifications (first PCR clean-up was done by using ExoSAP-IT express PCR clean up reagent, Thermo Fisher). Samples were sequenced on an Illumina MiSeq (PE 250 + 250) at the BioMicro Center (Massachusetts Institute of Technology, Cambridge, MA). Reads were processed using a custom pipeline where cutadapt was used for primer trimming, QIIME 1.9 [38] was used for demultiplexing, and DADA2 [15] was used to infer amplicon sequence variants (ASVs). Default settings were used except forward reads were truncated to 200 base pairs, and reverse reads were truncated to 175 base pairs before merging. Communities with less than 2000 reads were removed. ASVs that were more than 2% in more than 20% of the blank samples were considered as contaminants and also removed. Taxonomy for the sequence variants was assigned using the RDP database [39]. 16S copy number correction was performed with microbiome helper [40]: sequence variants were combined with the Greengenes database v13.5 [41] to build a new reference tree using FastTree [42], and assigned copynumbers using PICRUST [43].

### Calculation of **RTF**_C_

For *RTF*_C_ calculation, the monoculture functions measured from the second stage dilution-to-extinction was used as *F*_*i*_. *F*_C,*i*_ was calculated from *F*_C_*R*_C*,i*_, where *F*_C_ is the total community function and *R*_C*,i*_ is the relative abundance of the of taxon *i* in community C. This is only completely accurate when the community function of study is cell density, since different taxa may have different function to cell ratios. Thus, for community functions other than cell density,

$$F_{{\mathrm{C}},i} = \frac{{a_{{\mathrm{c}},i}}}{{a_{{\mathrm{c}},i}\left( {{\mathrm{cell}}} \right)}}\frac{{\overline {a_{{\mathrm{c}},i\left( {{\mathrm{cell}}} \right)}} }}{{\overline {a_{{\mathrm{c}},i}} }}F_{\mathrm{C}}R_{{\mathrm{C}},i}$$

*F*_C_*R*_C,*i*_ can be used as an approximation for *F*_C*,i*_ as long as the adjustment factor $\frac{{a_{{\mathrm{c}},i}}}{{a_{{\mathrm{c}},i}\left( {{\mathrm{cell}}} \right)}}\frac{{\overline {a_{{\mathrm{c}},i\left( {{\mathrm{cell}}} \right)}} }}{{\overline {a_{{\mathrm{c}},i}} }}$ is close to 1. Under the null model, the adjustment factor becomes $\frac{{a_i}}{{a_i\left( {cell} \right)}}\left( {\frac{{\overline {a_{i\left( {cell} \right)}} }}{{\overline {a_i} }}} \right)_C$ for each taxa in a community, which we find to be between the ranges of 0.5–2 for the majority of our taxa in communities (Fig. S8). The overall effect of these adjustment factors should be even closer to one when they are further averaged across different taxa in a community for calculating *RTF*_*C*_. Thus, for simplicity, the adjustment factor was assumed to be 1 for all taxa in communities, i.e., the protein/cell ratio and CO_2_/cell ratio among taxa in a community were equal.

Other estimations and assumptions used to calculate *RTF*_C_ included: (i) The criteria for determining if a sample was a monoculture was that at least 90% of the reads in the sample belonged to one sequence variant; the functions of the community was directly used as the monoculture functions. (ii) The criteria for a community to be “constitutable” was that 85% of the reads were covered by sequence variants of which we had monoculture functions. The constitutable parts of the communities were re-normalized so that the relative abundances of the sequence variants added up to 100%. (iii) CO_2_ production from 0 to 40 h were used for *RTF*_C_ calculations for CO_2_, since community composition measurements taken at early stationary phase were all around 40 h.

### Estimation of relative CUE

Since ${\mathrm{Ratio}}\,_{{\mathrm{ RTF}}_{\mathrm{c}}} = \frac{{{\mathrm{RTF}}_{{\mathrm{C}}\left( {{\mathrm{CO}}_{2}} \right)}}}{{{\mathrm{RTF}}_{{\mathrm{C}}\left( {{\mathrm{Protein}}} \right)}}} = {\frac{{\frac{{a_{{\mathrm{c}},i}}}{{a_i}}\left( {{\mathrm{CO}}_2} \right)}}{{{\frac{{a_{{\mathrm{c}},i}}}{{a_i}}\left( {{\mathrm{Protein}}} \right)} }}}$, and $\frac{{a_{{\mathrm{c}},i}}}{{a_i}}$ is a ratio, we can alter the units of *a*_*i*_ so that *a*_*i*(Protein)_ and *a*_*i*(CO2)_ are expressed in the same units, i.e., number of carbons in protein/number of carbon uptaken. Now *a*_*i*(Protein)_ is equivalent to CUE, and *a*_*i*(CO2)_ is equivalent to 1-CUE. By definition, *a*_C*,i*(Protein)_ is relative-to-expected CUE (denoted as *rCUE*) times CUE. Thus, by seeing the whole community as the behavior as an “average taxa”, $\frac{{{\mathrm{RTF}}_{{\mathrm{C}}\left( {{\mathrm{CO}}_{2}} \right)}}}{{{\mathrm{RTF}}_{{\mathrm{C}}\left( {{\mathrm{Protein}}} \right)}}} = \frac{{\frac{{1 - rCUE \ast {\mathrm{CUE}}}}{{1 - {\mathrm{CUE}}}}}}{{\frac{{rCUE \ast {\mathrm{CUE}}}}{{{\mathrm{CUE}}}}}}$. This gives

$$rCUE = \frac{1}{{{\mathrm{Ratio}}\,_{{\mathrm{ RTF}}_{\mathrm{c}}}\left( {1 - {\mathrm{CUE }}} \right) + {\mathrm{CUE}}}}$$

Estimation of relative CUE depending on the ratio between *RTF*_C(CO2)_ and *RTF*_C(Protein)_ was performed according to the equation above and by setting up the expected CUE in intervals of 0.01, ranging from 0 to 0.6.

### Curve fitting

All curve fitting was performed using the nls function in R [44], and the 95% confidence interval of the curvefits were calculated using uncertainty propagation by first-/second-order Taylor expansion and Monte Carlo simulation including covariances using the package “propagate” [45]. A linear, log-linear, hyperbolic least squares fit was performed for each dataset, and the model with the least AIC or an AIC comparable to the least AIC was selected. All sum of squares calculations were type II, and performed using the function “Anova” in the R package “car” [46].

### Diversity calculations

Species richness was determined as the number of ASVs in each community, and they were compared against a rarefactioned species richness, using the R package “vegan” [47], and an estimated species richness, using the R package “breakaway” [16]. MPD and MNTDs of each community were calculated by first performing a multiple-alignment using the R package “DECIPHER” [48], then constructing a GTR + G+I (Generalized time-reversible with Gamma rate variation) maximum likelihood tree with the R package “phangorn” [49], and calculating the actual values using the R package “picante” [50].

## Supplementary information

Supplemental Figures

Supplemental Tables

## Data Availability

All amplicon sequencing data generated in this study can be accessed upon publication on the US National Center for Biotechnology Information SRA database under BioProject PRJNA477654. All community function measurements, ASV tables, and code for data analysis will available at https://github.com/cusoiv.
