# Supplementary material for: Interactions in self-assembled microbial communities saturate with diversity
Source: ISME J. 2019 Feb 26;13(6):1602–17. doi: 10.1038/s41396-019-0356-5 (PMC6775987; doi:10.1038/s41396-019-0356-5)
Supplement: Supplementary file 1 — Supplemental Figures [file 41396_2019_356_MOESM1_ESM.docx]

**
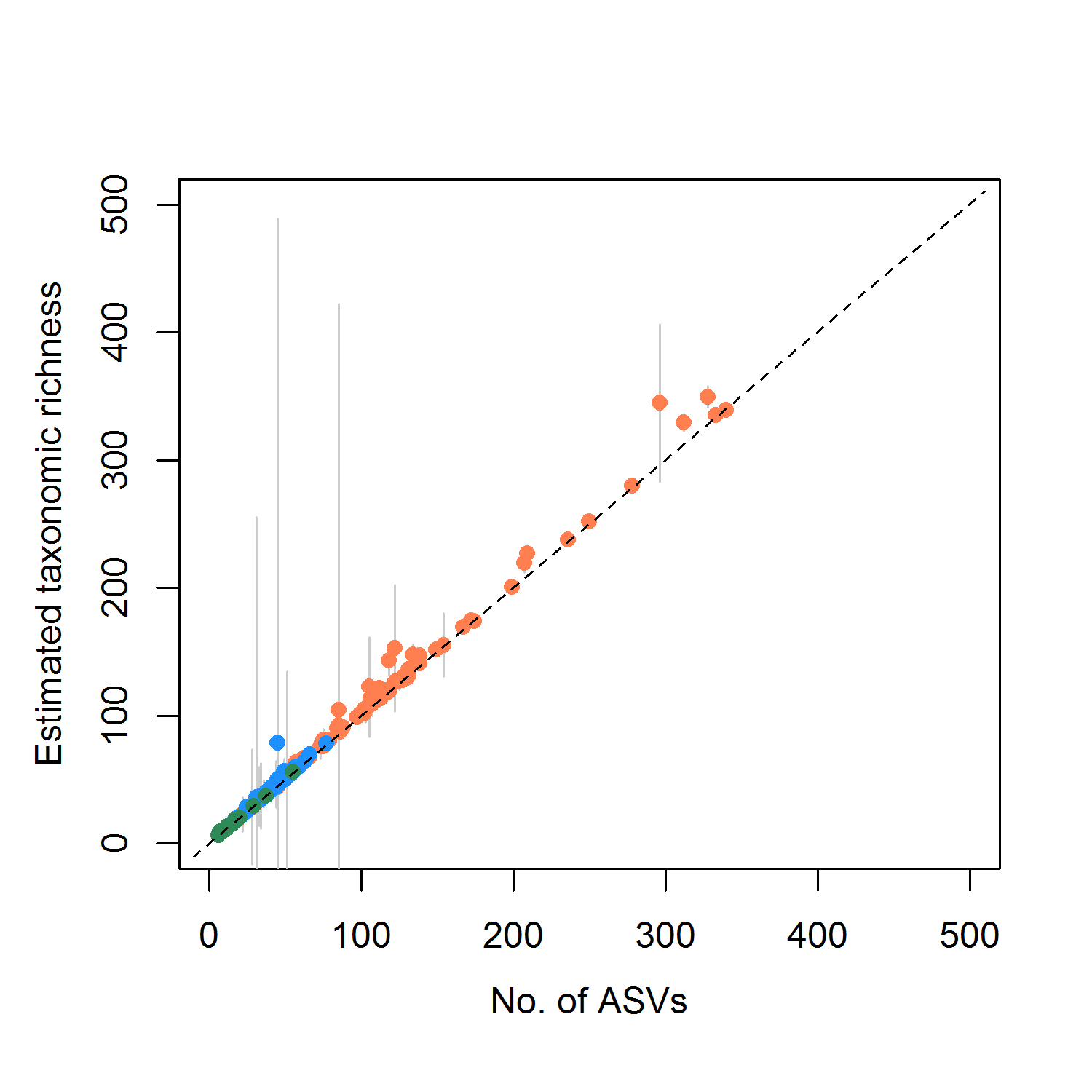
Figure S1**

**
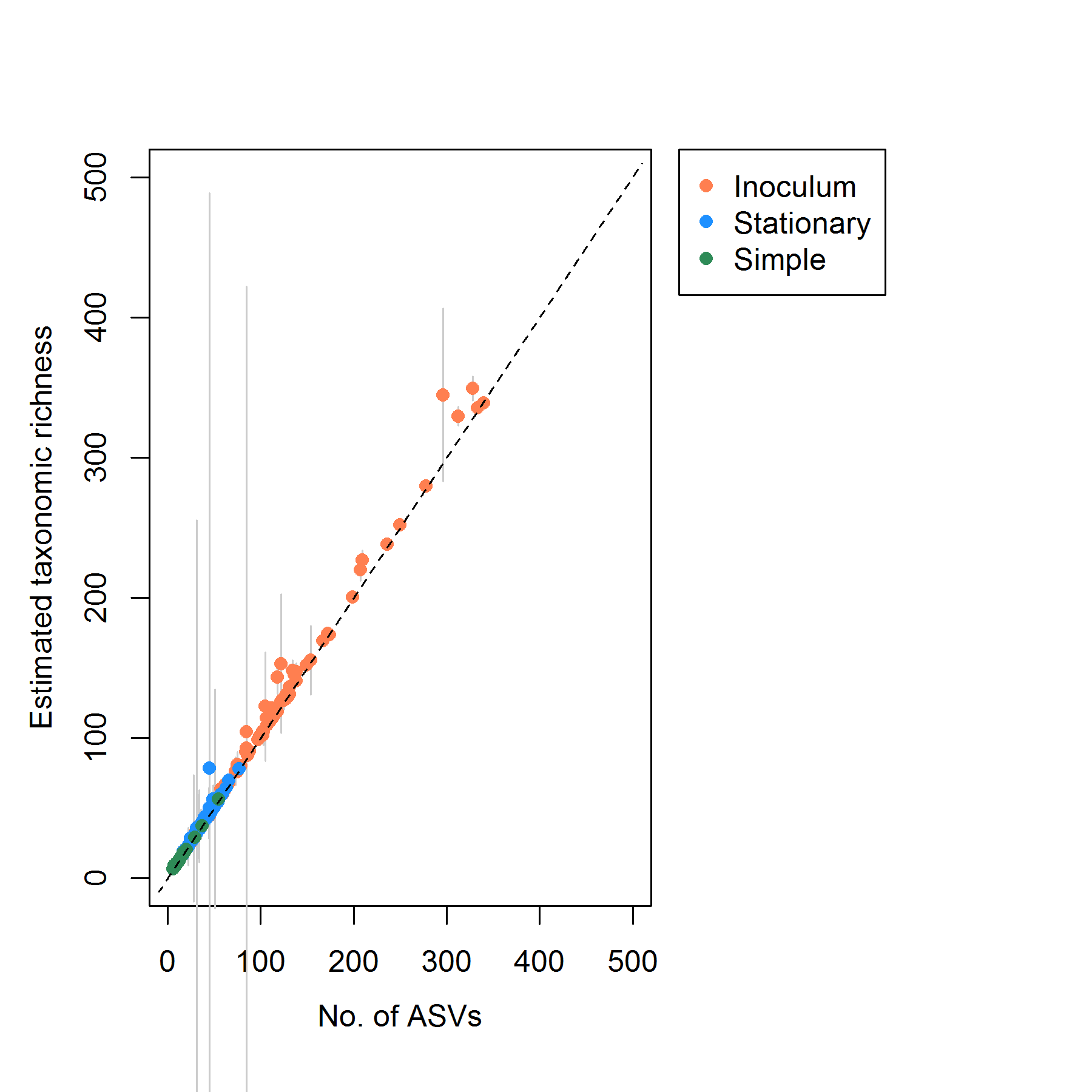
**

**Figure S1 Estimated taxonomic richness are almost identical to ASV counts.** Relationship between the number of ASVs counted directly from sequencing data and estimated after accounting for uncounted ASVs due to finite sequencing depth for all sequenced communities. Only communities that fit the estimation criterion (at least 6 different read frequencies) are shown. Grey bars, standard error of the estimate; broken line, estimated taxonomic richness=counted number of ASVs.

**Figure S2**

**
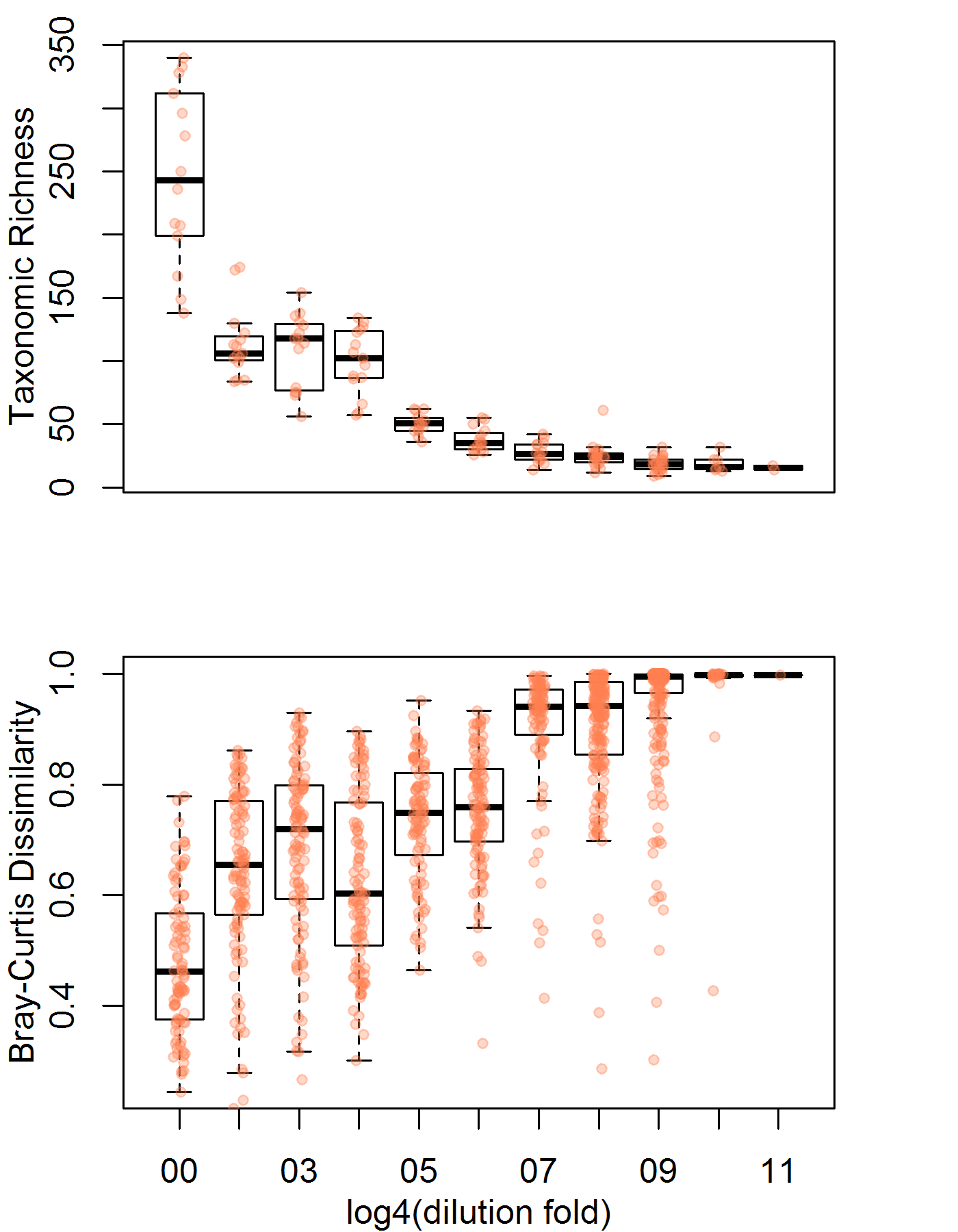
**
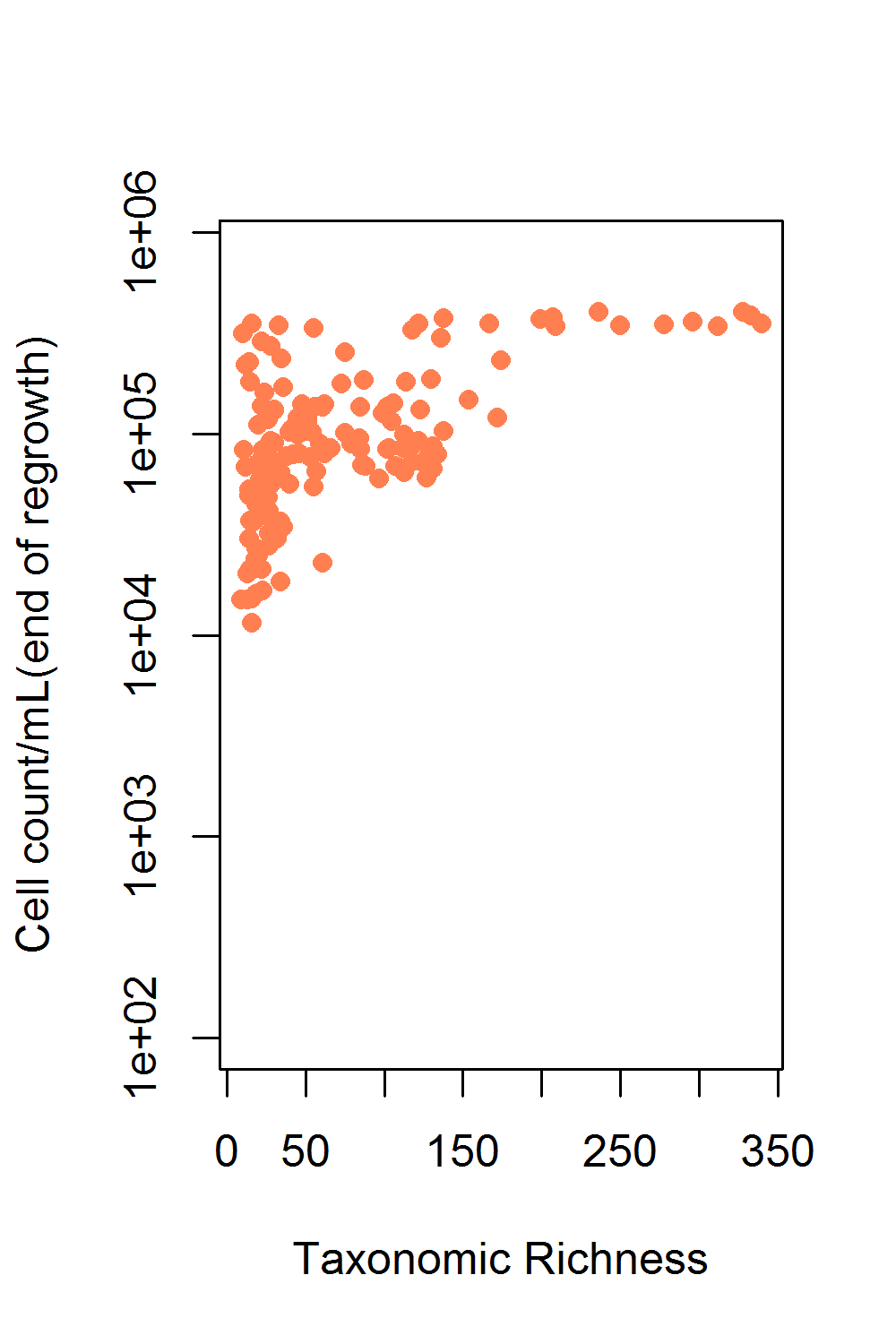


**a b**

**c**

**Figure S2 Effects of dilution-regrowth of microbial communities in pasterized seawater.** (a) Taxonomic richness and cell denisty of all inoculum communities. (b) Relationship of dilution factor and taxonomic richness. (c) Bray-Curtis dissimilarity between any two communities with the same dilution factor. Each red dot represents one community; the black line in box plots represent the median; wiskers represent 95% confidence interval of the median.

**
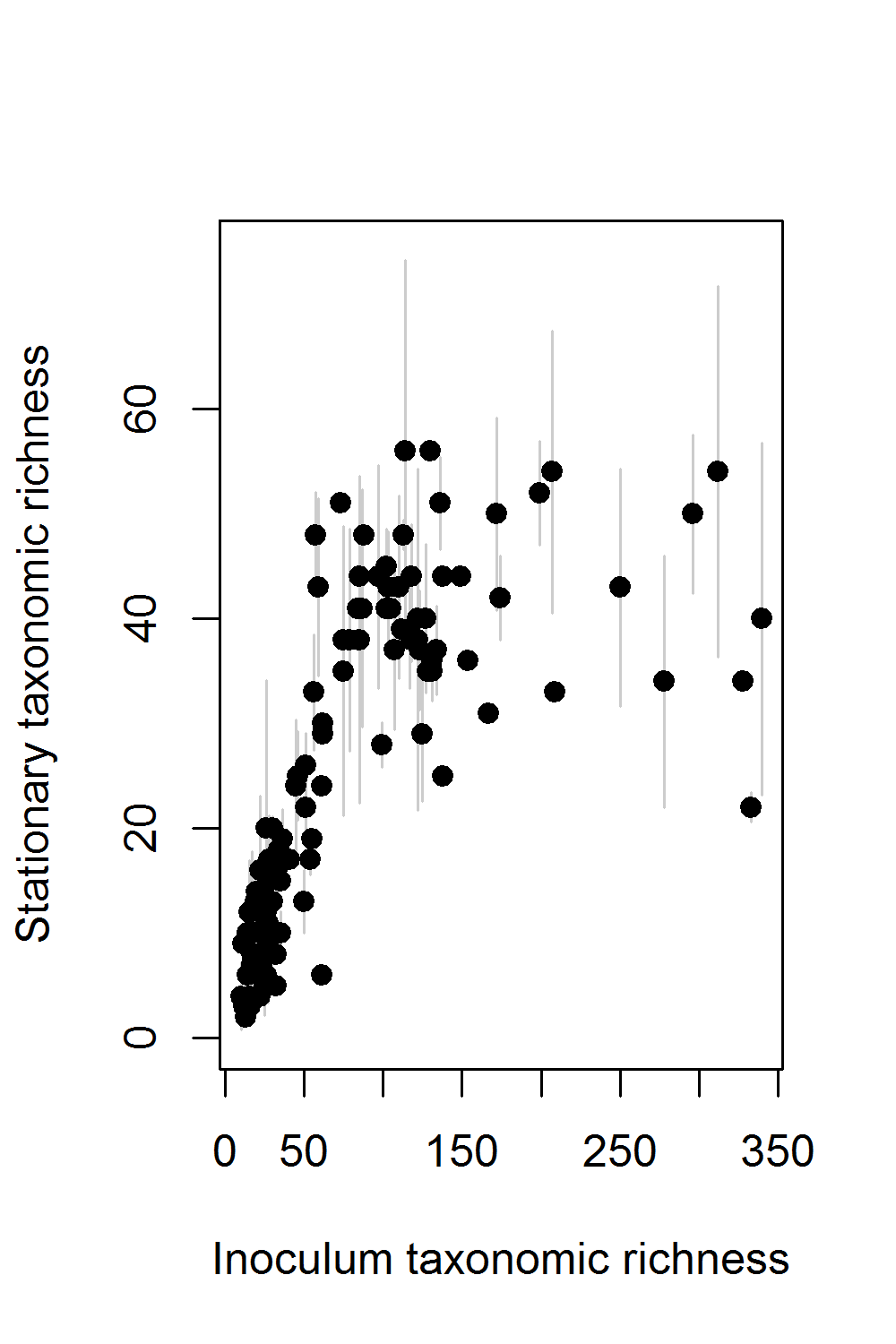

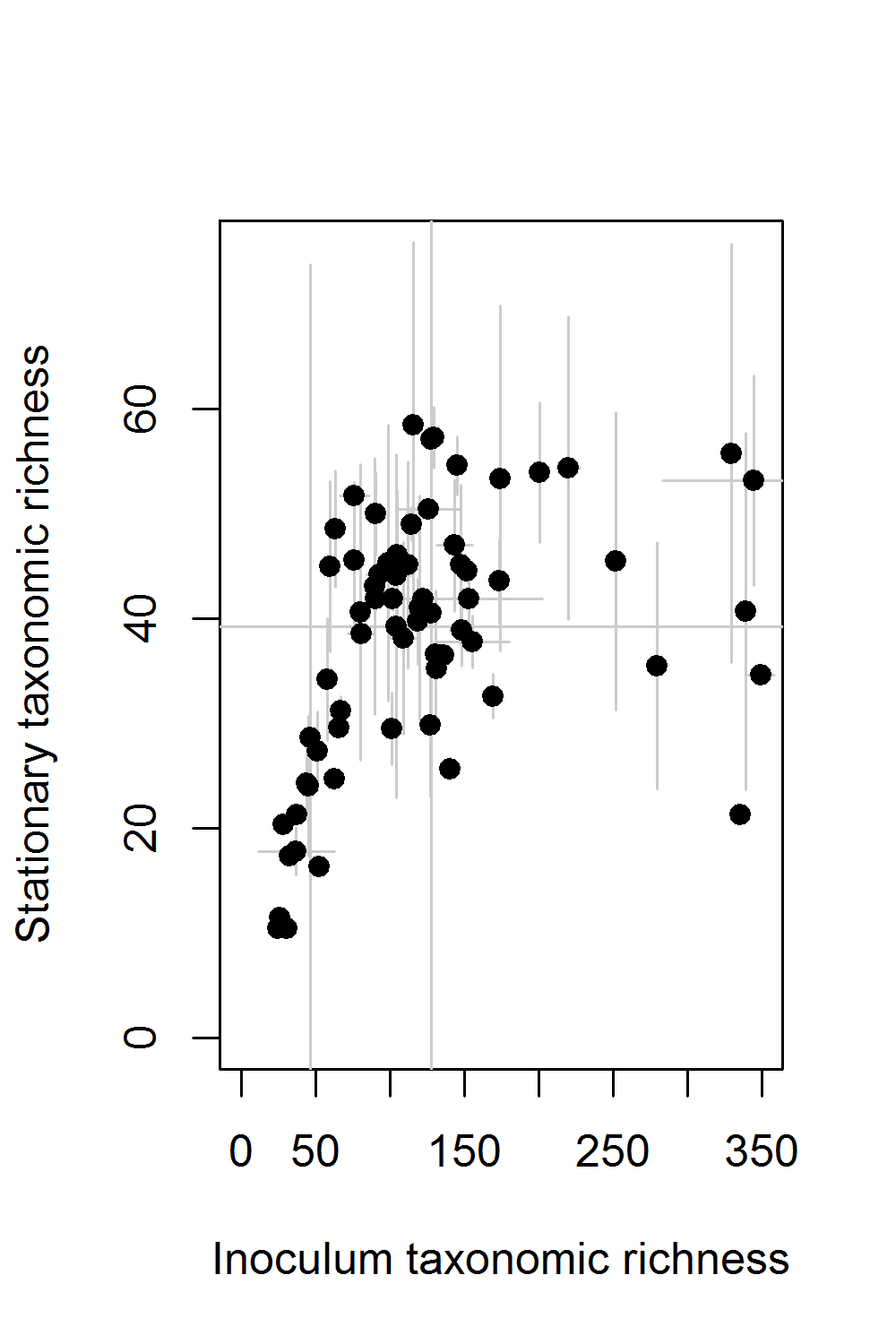
Figure S3**

**a b**

**Figure S3 Relationship between inoculum taxonomic richness and stationary taxonomic richness.** Taxonomic richness displayed as (a) direct ASV counts and (b) estimated taxonomic richness. Each black dot represents one inoculum community, and error bars are standard deviations over three biological replicates derived from the same inoculum community. Error bars in b) include the propagated standard error of the taxonomic richness estimates.

**Figure S4**

**
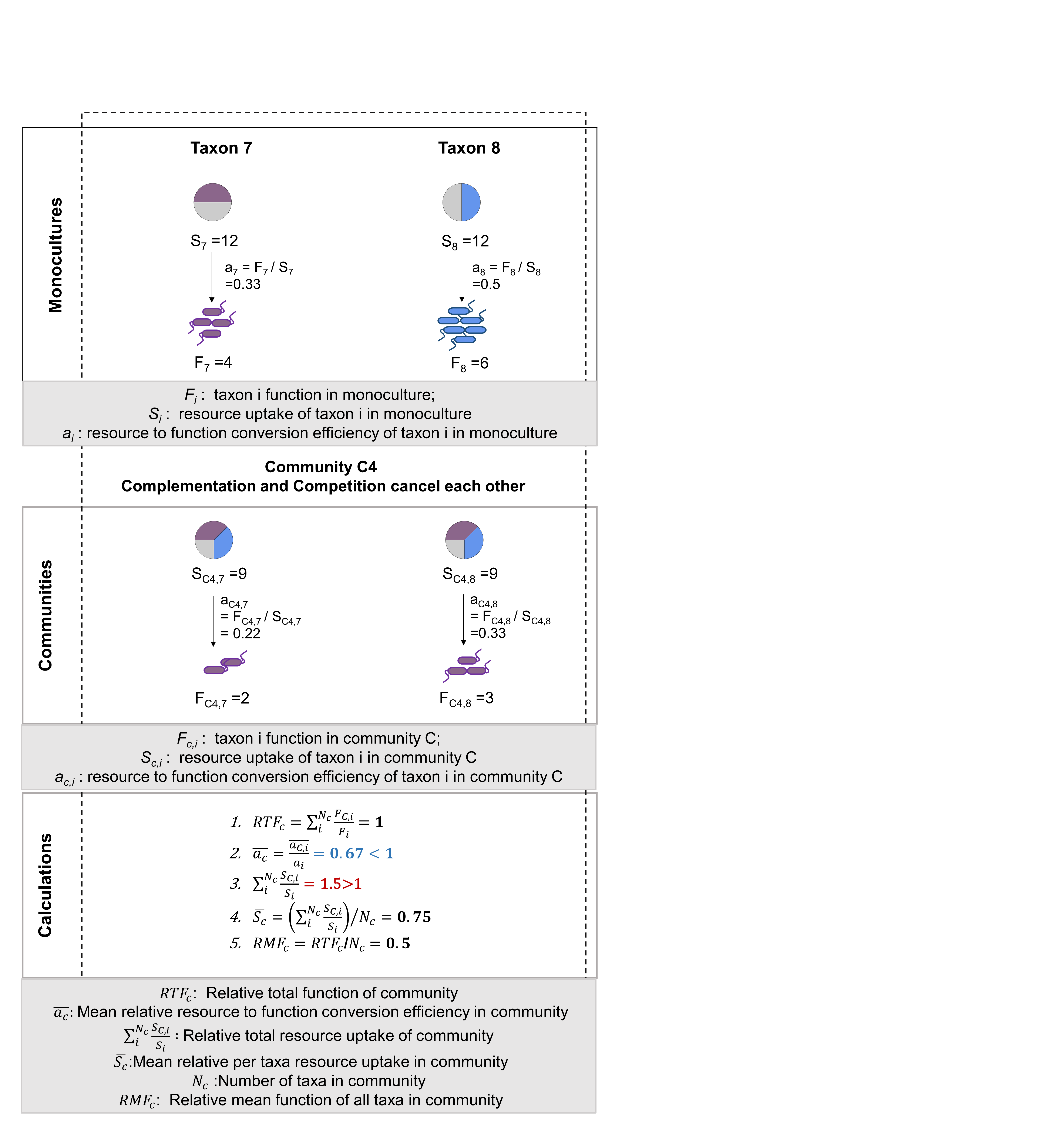
**

**Figure S4 Extended graphical illustration of the RTF concept.** Illustration of a case where RTF_c_ equals 1 but is caused by interactions having positive effects on the relative total resource uptake ($\sum_{i}^{N_{c}} \frac{S_{C,i}}{S_{i}})$ and negative effects on the mean relative resource to function conversion efficiency ($\bar{a_{c}})$. The purpose of this illustration is to emphasize there is a difference between interactions having no effects on community function (only requires RTF_c_ equal 1), and no interactions having effects on community function (requires RTF_c,_$\sum_{i}^{N_{c}} \frac{S_{C,i}}{S_{i}}$, and $\bar{a_{c}}$ to all be 1). Our null model corresponds to the latter case.

**Figure S5**


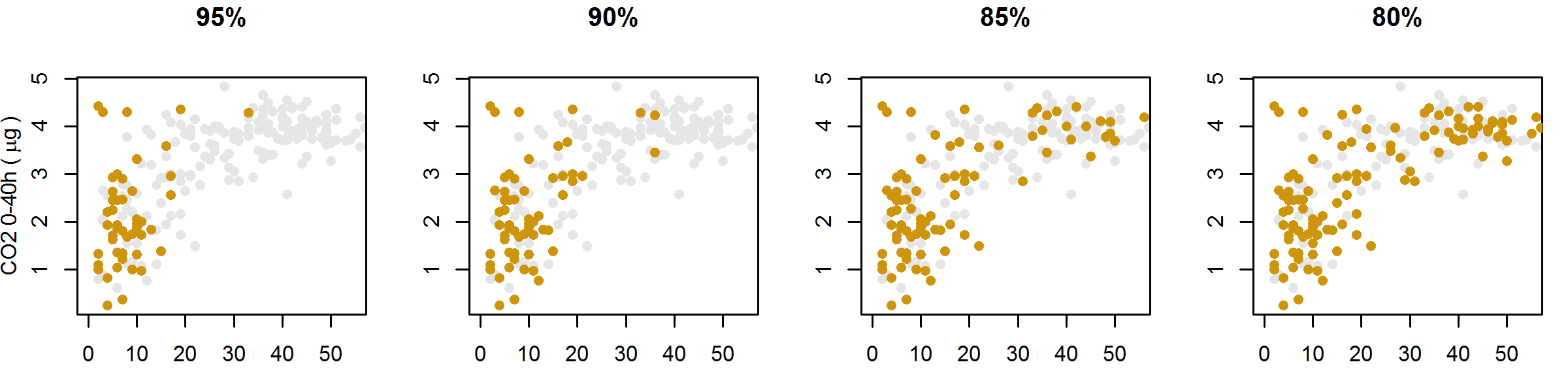

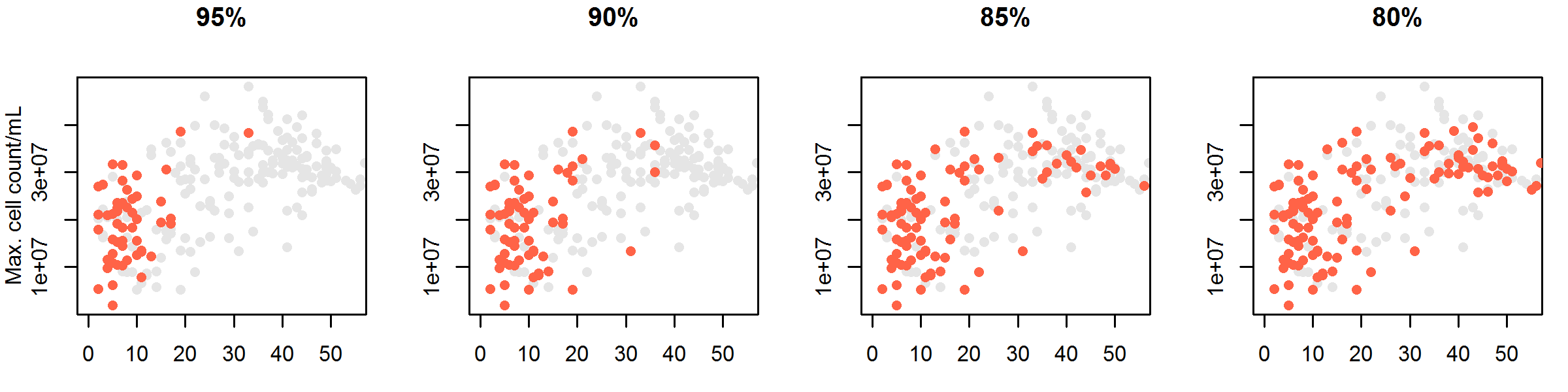

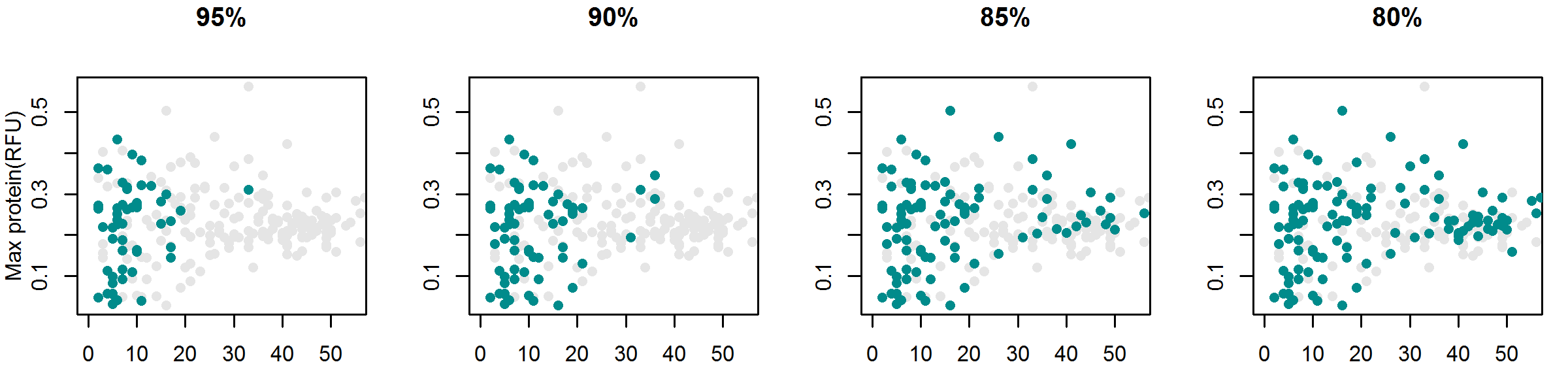


**
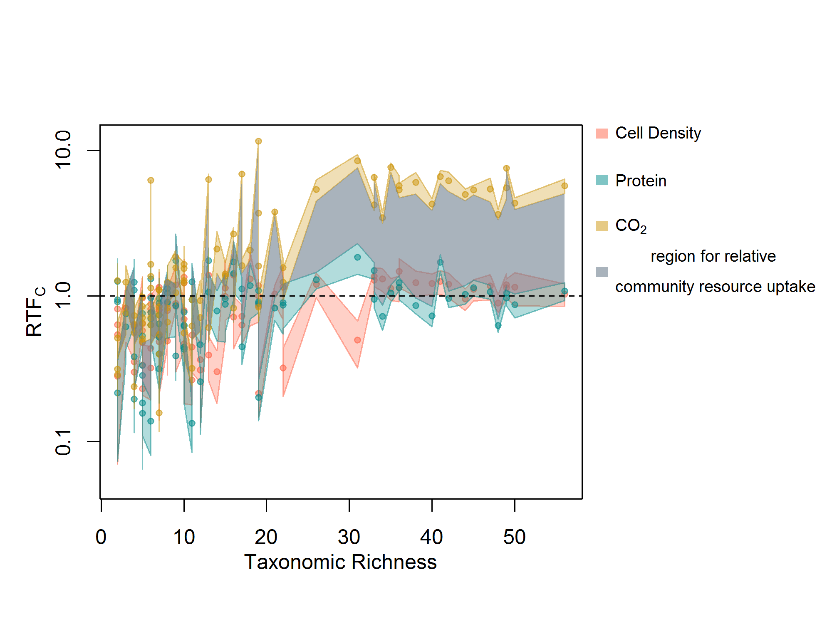
**

**Figure S5** **Constitutable communities under different community coverage criteria.** Relaxing criteria (95%, 90%, 85%, 80% reads covered by taxa with known monoculture functions) for constitutable communities increase their diversity coverage. Final criteria selected for constitutable communities was 85% coverage since it was the highest standard that allowed sufficient coverage of diversity. Colored dots, communities with production and respiration measurements that are constitutable; grey dots, communities with production and respiration measurements but are not constitutable.

**Figure S6**

**
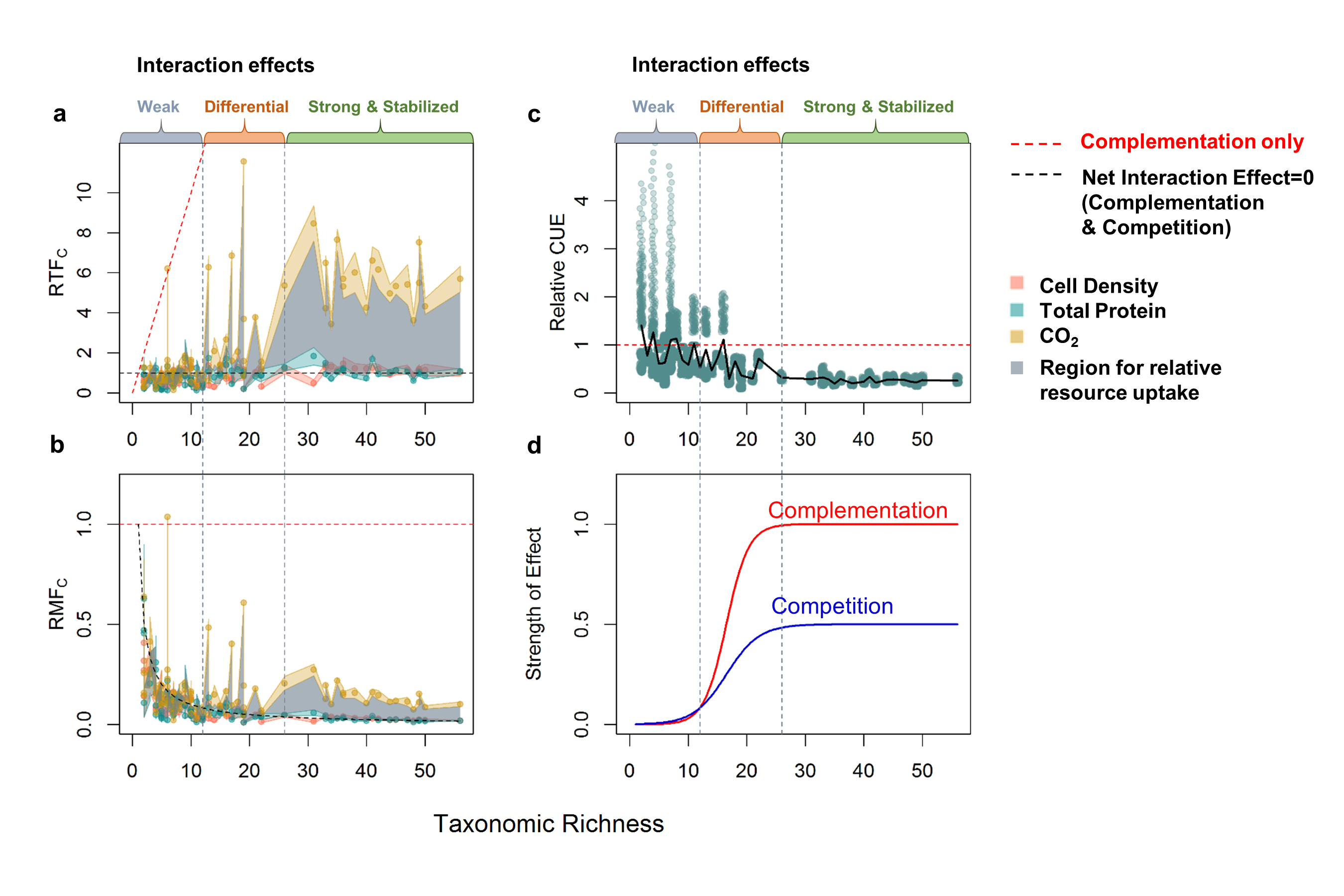
**

**Figure S6 Relative community-to-monoculture functions indicate differential increases in niche complementation and competition with diversity (linear scale).** Relationship between diversity and (a) relative total community biomass production, respiration, and resource uptake, and (b) relative per taxa biomass production, respiration, and resource uptake (c) estimated relative carbon use efficiency (CUE). In (a), (b) each point represents one community at stationary phase (biological replicates are not averaged). In (c), each point represents a combination of one relative total function (${RTF}_{C})$ ratio with a random value of CUE drawn from 0-0.6. The black line represents the mean of all combinations with the same taxonomic richness. Colored regions around the points indicate the standard deviation of the interaction effect, and the grey areas indicate the range where relative total community and per taxa resource uptake is limited to at each taxonomic richness. (d) A hypothetical model for how the effects of niche complementation and competition on community function scales with taxonomic richness. Numbers on the y-axis are arbitrary.

**
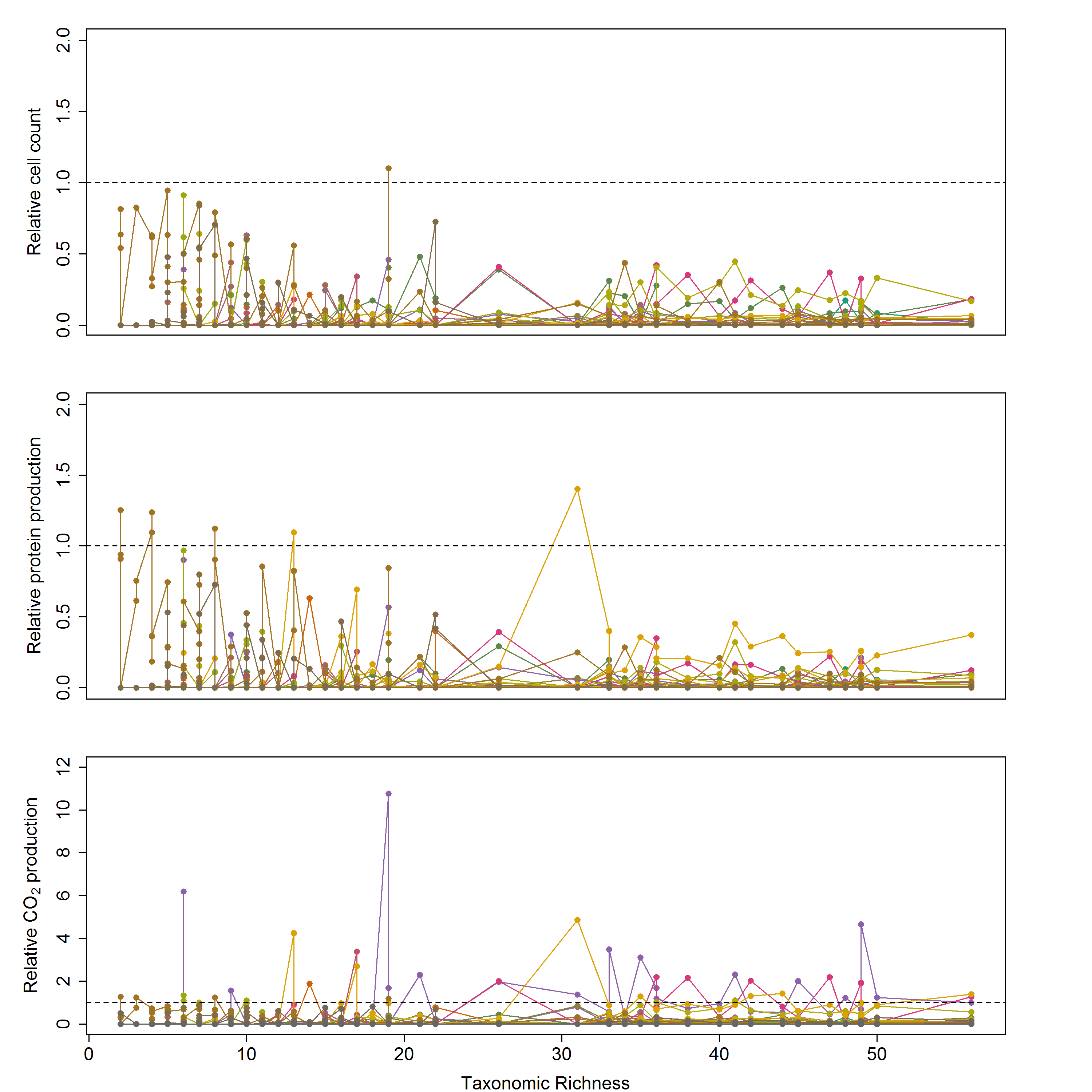
Figure S7**

**a**

**b**

**c**

**Figure S7 Relative function of all isolate taxa in communties.** The (a) relative cell count, (b) relative protein production, and (c) relative CO_2_ production for all the isolate taxa in communities compared to their monocultures. Each colored line with points represents one taxa. Dotted lines represent relative functions of 1.

**
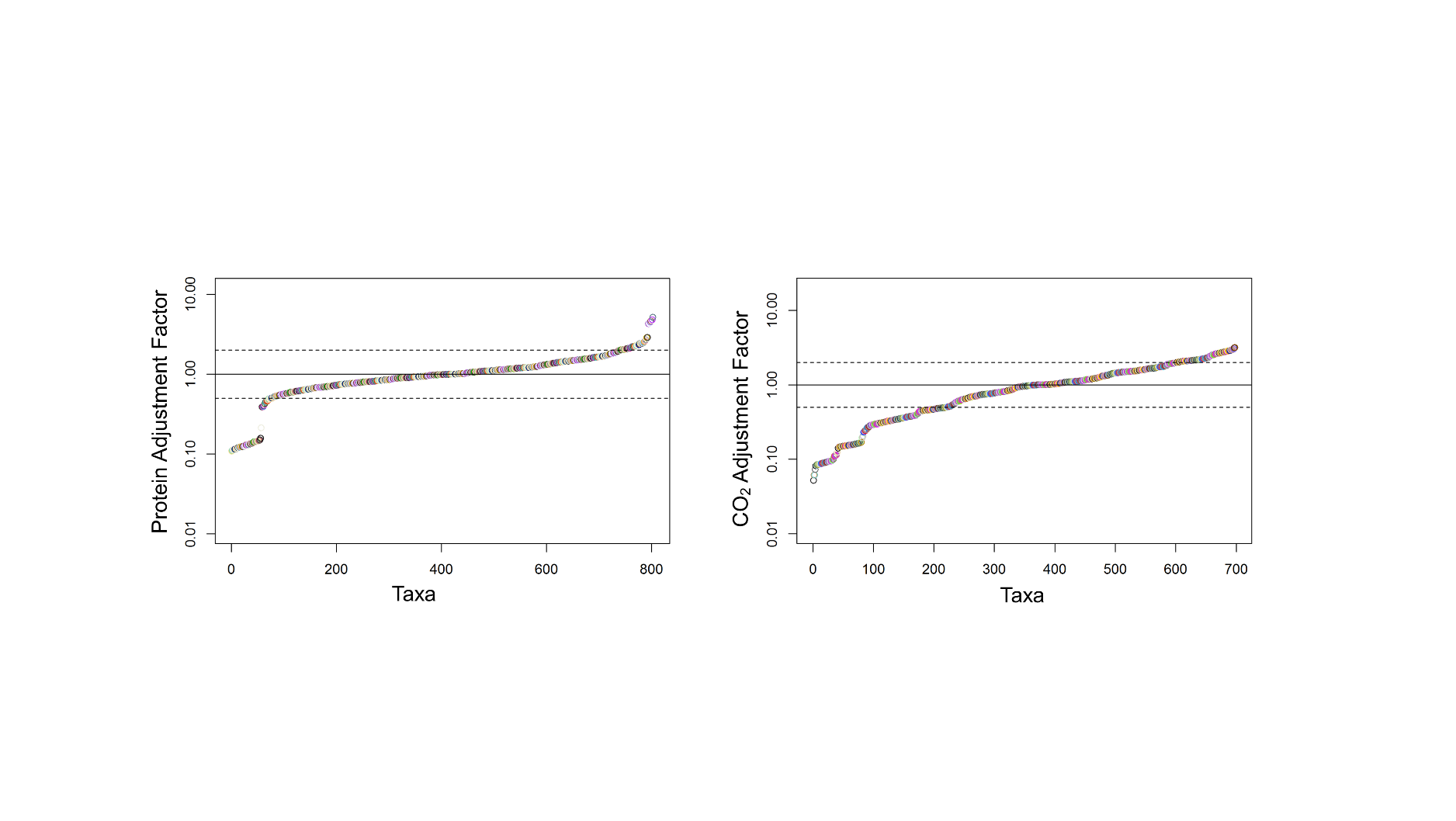
Figure S8**

**a b**

**Figure S8 Adjustment factors for all taxa in communities.** The adjustment factor for (a) protein measurements and (b) CO_2_ production (0-40h) for all taxa of non-zero abundance in communities. Dotted black lines represent the range 0.5-2.

**
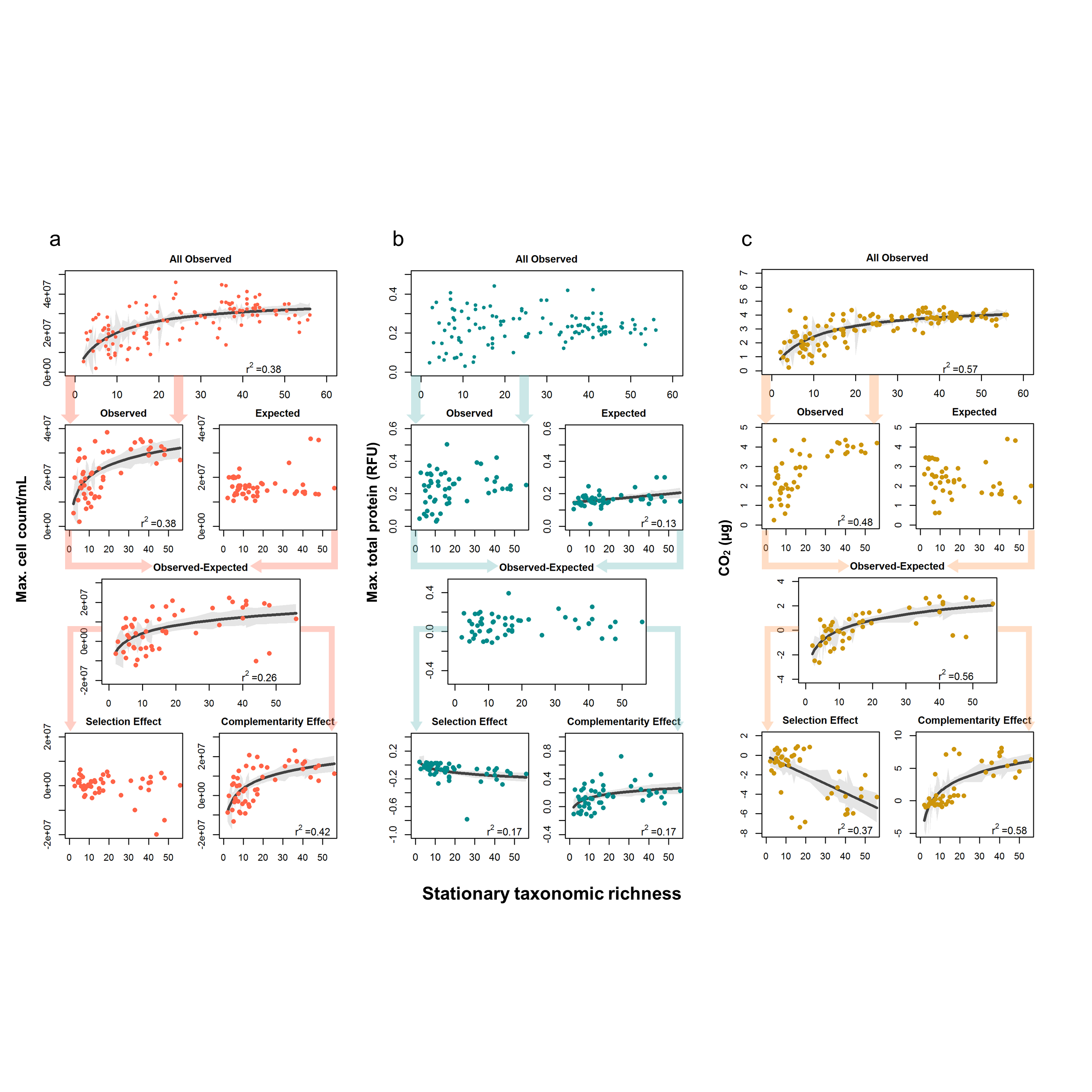
Figure S9**

**Figure S9 Additive partitioning of diversity-function relationships with the Loreau and Hector model.** Relationship between diversity as taxonomic richness at stationary phase and community function as (a) Maximum cell density the communities reached (b) Maximum measured protein production (c) total CO_2_ production in 0-40h. In (a), (b), and (c), the top panels labeled “All Observed” show all observed data points for relationships between stationary taxonomic richness and different measurements of community function. “Observed”, observed taxonomic richness and community function relationships for all constitutable communities. “Expected”, expected taxonomic richness and community function relationships under the null model. “Observed-Expected”, relationship between taxonomic richness and deviance between observed function and expected function of communities (NBE). “Observed-Expected” can further be broken down as the sum of “Selection Effect” and “Complementarity Effect”. “Selection Effect”, relationship between taxonomic richness and the selection effect on community function. “Complementarity effect”, relationship between taxonomic richness and the complementarity effect on productivity. Each dot represents one community; black lines indicate fits to an appropriate model between a linear, log-linear, and hyperbolic least squares fit, with gray regions around the line indicating the 95% confidence of the fit.

**Figure S10**

**
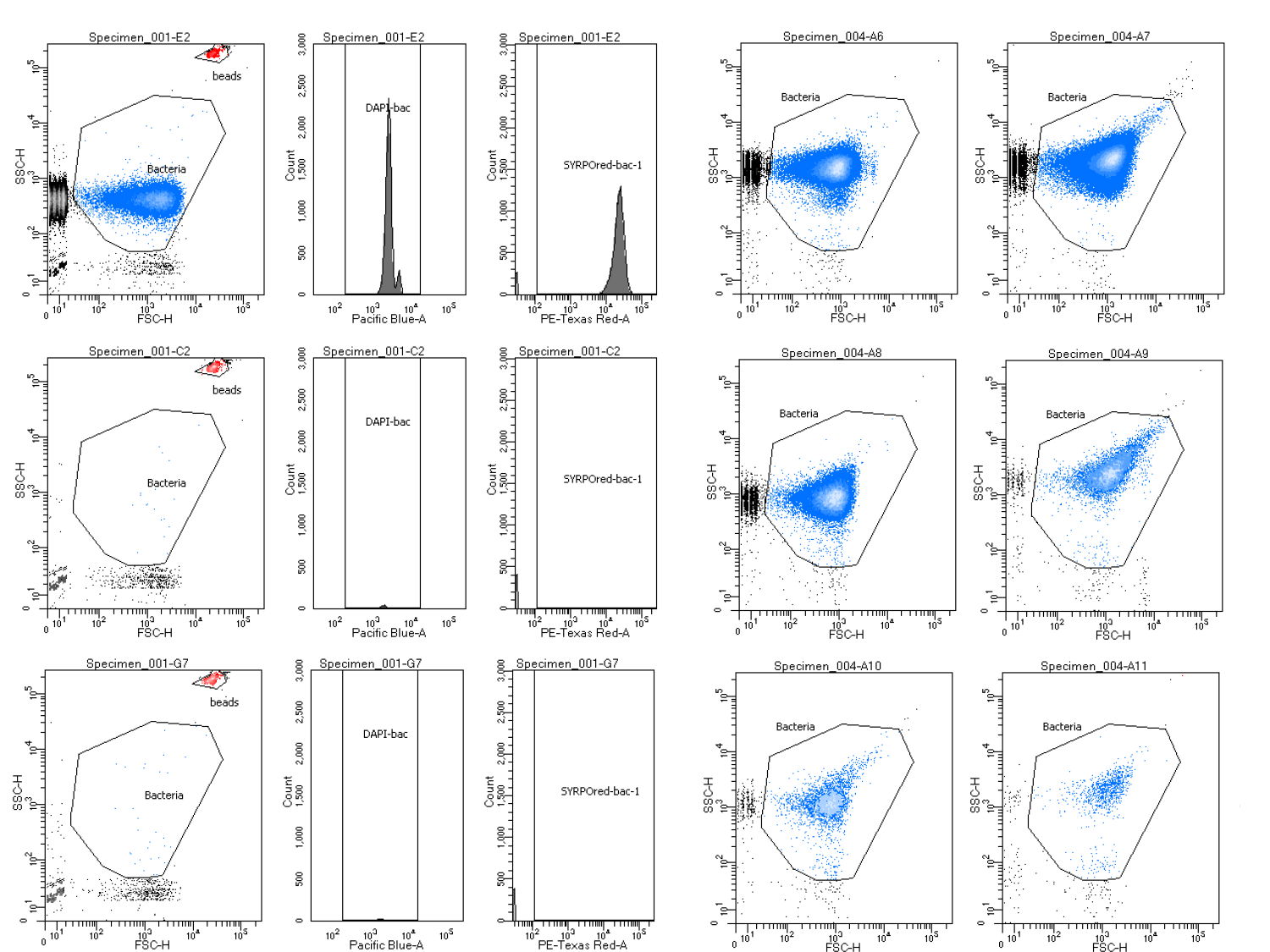
a**

**b**

**c**

**
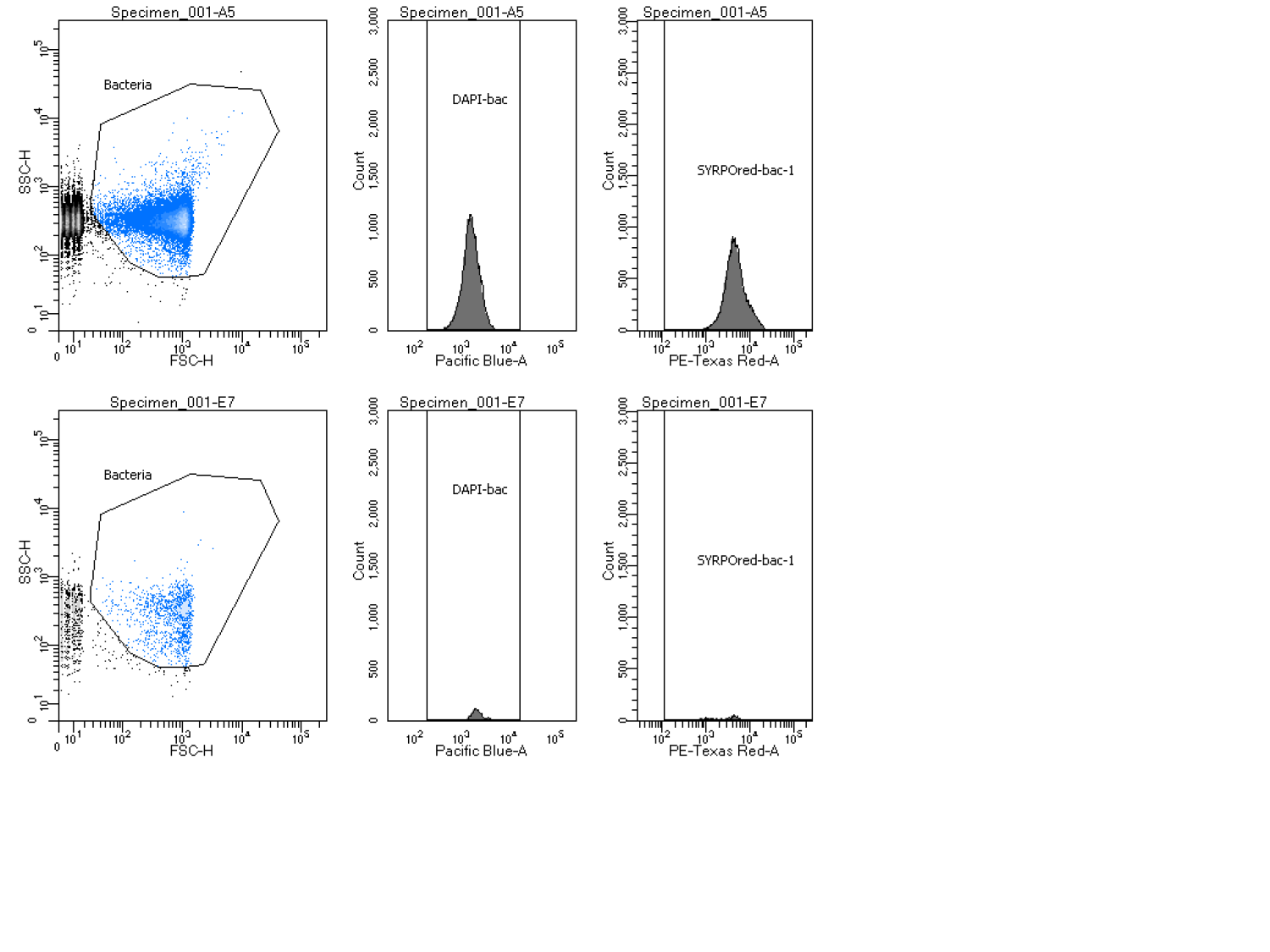
**

**d**

**e**

**
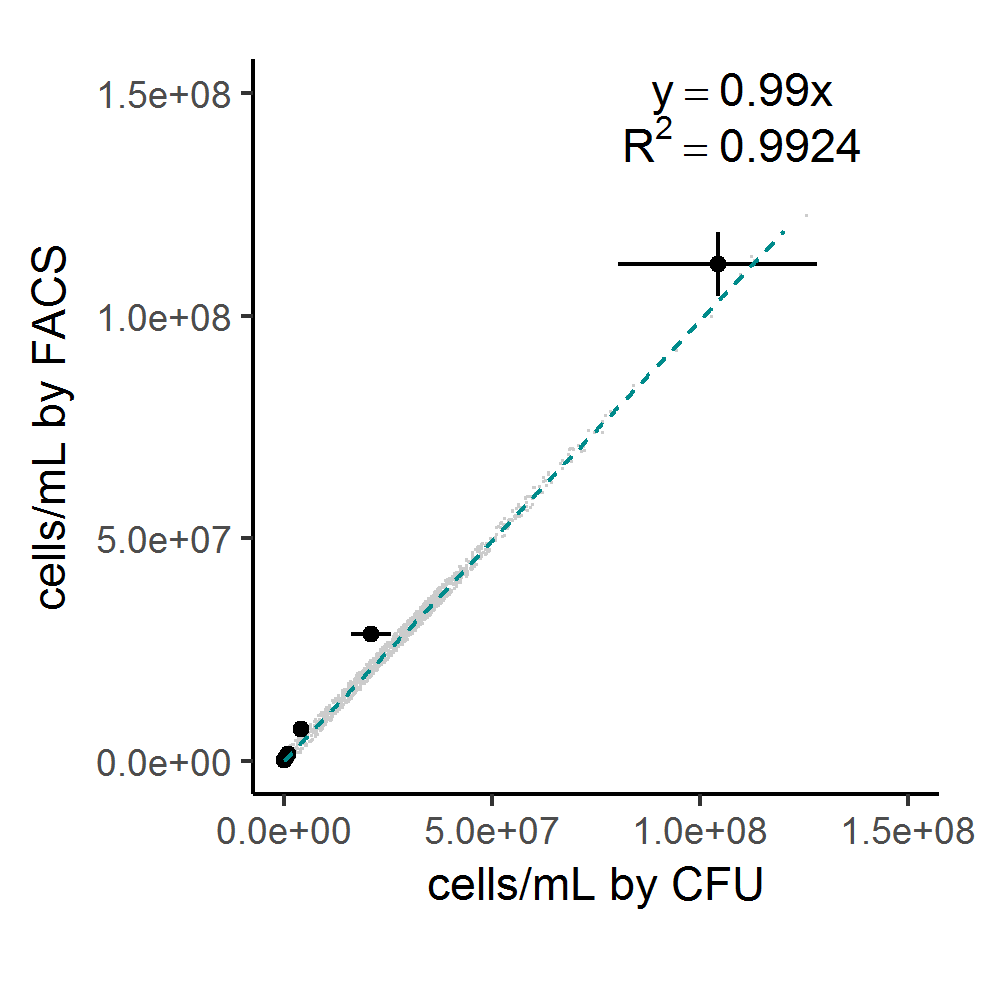
f**

**
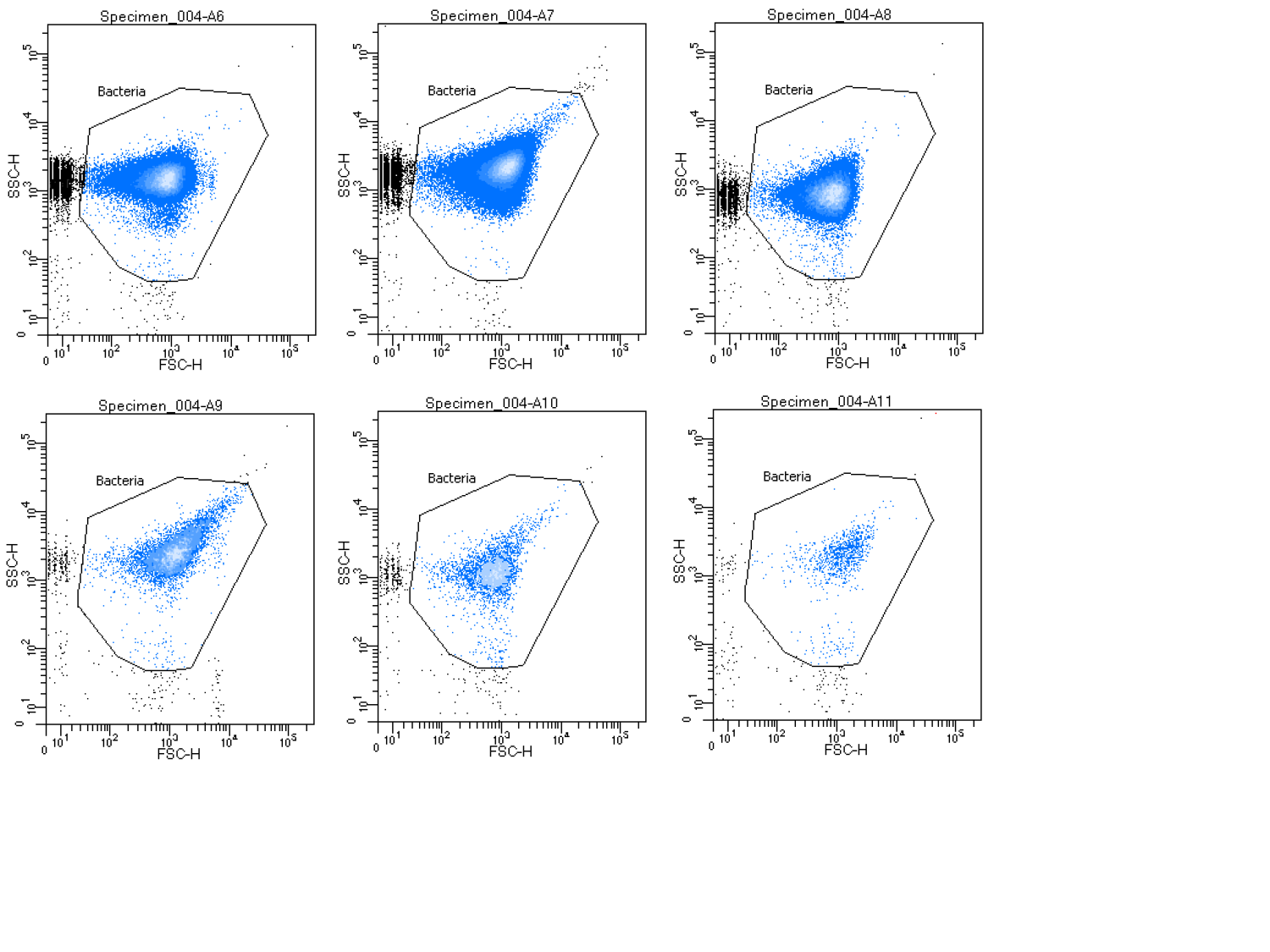
g h i**

**j k l**

**Figure S10 FACS histograms and calibrations.**  The 2D FSC-H, SSC-H density plots as well as fluorscence histograms for several bacterial isolates, communities of different diversity and controls.

(a) a *Vibrionaceae* strain at mid-log phase stained with DAPI and SYPRO red and 7 µm beads, (b) an unstained *Vibrionaceae* strain at mid-log phase and 7 µm beads, (c) 7 µm beads only, (d) a high diversity community at mid-log phase stained with DAPI and SYPRO red, and (e) a low diversity community at mid-log phase stained with DAPI and SYPRO red. (f) Comparison between FACS and CFU counts for the strain of *Vibrionaceae* from (a). Each black dot represent the average FACS counts/CFUs for three replicates, and error bars represent standard deviations of the measurement. The dark cyan line represents the fitted line that describes the relationship between FACS measurements and CFU counts. The grey shaded area represents all the FACS measurements (jittered for better visualization) in our experiment. The 2D FSC-H, SSC-H density plots for cultures of (g) *Pseudoalteromonadaceae*, (h) *Halomonadaceae*, (i) *Alteromonadaceae*, (j) *Rhodobacteraceae,* (k) *Flavobacteriaceae*, (l) *Oceanospirillaceae.*

**Figure S11**

**
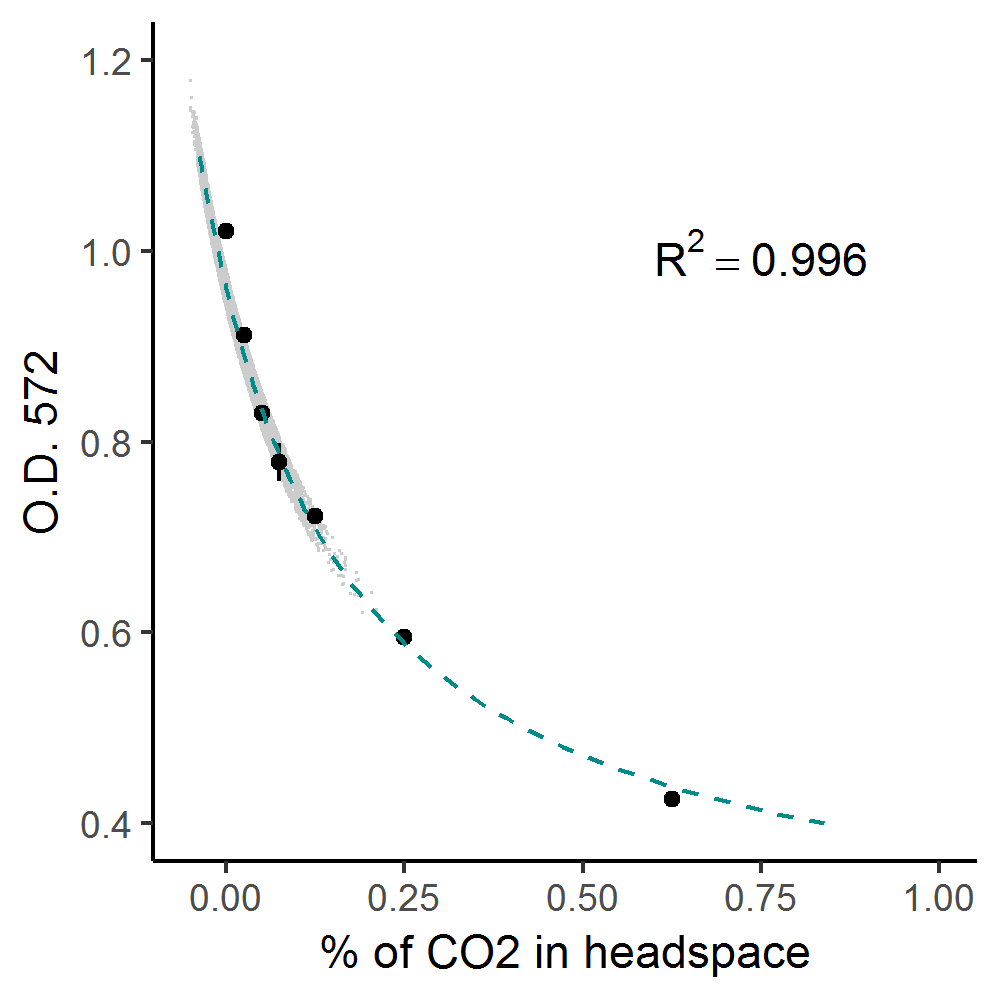
**

**Figure S11 Standard curve for CO_2_ measurements with Microresp colormetric assay.**  Each black dot represent the average O.D. for four replicates at the same CO_2_ concentration, and error bars represent standard deviations of the measurement. The dark cyan line represents the fitted standard curve (%CO2) =0.1648/ (Δ_572_-0.2457)-0.2301, and the grey shaded area represents all the raw O.D. measurements (jittered for better visualization) for CO_2_ in our experiment.
