# Supplementary material for: Interactions in self-assembled microbial communities saturate with diversity
Source: ISME J. 2019 Feb 26;13(6):1602–17. doi: 10.1038/s41396-019-0356-5 (PMC6775987; doi:10.1038/s41396-019-0356-5)
Supplement: Supplementary file 2 — Supplemental Tables [file 41396_2019_356_MOESM2_ESM.docx]

**Table S1-List of isolates**

|  | **Kingdom** | **Phylum** | **Class** | **Order** | **Family** | **Genus** | **Species** |
| --- | --- | --- | --- | --- | --- | --- | --- |
| ASV_13 | Bacteria | Bacteroidetes | Flavobacteriia | Flavobacteriales | Flavobacteriaceae | Algibacter | NA |
| ASV_71 | Bacteria | Bacteroidetes | Flavobacteriia | Flavobacteriales | Flavobacteriaceae | Algibacter | NA |
| ASV_33 | Bacteria | Bacteroidetes | Flavobacteriia | Flavobacteriales | Flavobacteriaceae | Maribacter | NA |
| ASV_18 | Bacteria | Bacteroidetes | Flavobacteriia | Flavobacteriales | Flavobacteriaceae | Polaribacter | NA |
| ASV_40 | Bacteria | Bacteroidetes | Flavobacteriia | Flavobacteriales | Flavobacteriaceae | Polaribacter | NA |
| ASV_30 | Bacteria | Bacteroidetes | Flavobacteriia | Flavobacteriales | Flavobacteriaceae | Wenyingzhuangia | NA |
| ASV_55 | Bacteria | Bacteroidetes | Flavobacteriia | Flavobacteriales | Flavobacteriaceae | Zobellia | russellii |
| ASV_28 | Bacteria | Proteobacteria | Alphaproteobacteria | Rhodobacterales | Rhodobacteraceae | Celeribacter | NA |
| ASV_64 | Bacteria | Proteobacteria | Alphaproteobacteria | Rhodobacterales | Rhodobacteraceae | Labrenzia | NA |
| ASV_29 | Bacteria | Proteobacteria | Alphaproteobacteria | Rhodobacterales | Rhodobacteraceae | Litoreibacter | NA |
| ASV_26 | Bacteria | Proteobacteria | Alphaproteobacteria | Rhodobacterales | Rhodobacteraceae | Loktanella | NA |
| ASV_8 | Bacteria | Proteobacteria | Alphaproteobacteria | Rhodobacterales | Rhodobacteraceae | Loktanella | pontiacus |
| ASV_20 | Bacteria | Proteobacteria | Alphaproteobacteria | Rhodobacterales | Rhodobacteraceae | NA | NA |
| ASV_23 | Bacteria | Proteobacteria | Alphaproteobacteria | Rhodobacterales | Rhodobacteraceae | NA | NA |
| ASV_38 | Bacteria | Proteobacteria | Alphaproteobacteria | Rhodobacterales | Rhodobacteraceae | NA | NA |
| ASV_42 | Bacteria | Proteobacteria | Alphaproteobacteria | Rhodobacterales | Rhodobacteraceae | NA | NA |
| ASV_45 | Bacteria | Proteobacteria | Alphaproteobacteria | Rhodobacterales | Rhodobacteraceae | NA | NA |
| ASV_72 | Bacteria | Proteobacteria | Alphaproteobacteria | Rhodobacterales | Rhodobacteraceae | NA | NA |
| ASV_88 | Bacteria | Proteobacteria | Alphaproteobacteria | Rhodobacterales | Rhodobacteraceae | NA | NA |
| ASV_89 | Bacteria | Proteobacteria | Alphaproteobacteria | Rhodobacterales | Rhodobacteraceae | NA | NA |
| ASV_52 | Bacteria | Proteobacteria | Alphaproteobacteria | Rhodobacterales | Rhodobacteraceae | Oceanicola | NA |
| ASV_43 | Bacteria | Proteobacteria | Alphaproteobacteria | Rhodobacterales | Rhodobacteraceae | Roseovarius | nubinhibens |
| ASV_16 | Bacteria | Proteobacteria | Alphaproteobacteria | Rhodobacterales | Rhodobacteraceae | Sulfitobacter | arcticus |
| ASV_12 | Bacteria | Proteobacteria | Alphaproteobacteria | Rhodobacterales | Rhodobacteraceae | Sulfitobacter | dubius |
| ASV_41 | Bacteria | Proteobacteria | Alphaproteobacteria | Rhodobacterales | Rhodobacteraceae | Sulfitobacter | NA |
| ASV_9 | Bacteria | Proteobacteria | Alphaproteobacteria | Rhodobacterales | Rhodobacteraceae | Sulfitobacter | NA |
| ASV_44 | Bacteria | Proteobacteria | Alphaproteobacteria | Rhodobacterales | Rhodobacteraceae | Thalassobius | NA |
| ASV_31 | Bacteria | Proteobacteria | Alphaproteobacteria | Rhodospirillales | Rhodospirillaceae | Thalassospira | NA |
| ASV_3 | Bacteria | Proteobacteria | Gammaproteobacteria | Alteromonadales | Alteromonadaceae | Alteromonas | NA |
| ASV_34 | Bacteria | Proteobacteria | Gammaproteobacteria | Alteromonadales | Alteromonadaceae | Alteromonas | NA |
| ASV_17 | Bacteria | Proteobacteria | Gammaproteobacteria | Alteromonadales | Alteromonadaceae | Paraglaciecola | NA |
| ASV_6 | Bacteria | Proteobacteria | Gammaproteobacteria | Alteromonadales | Pseudoalteromonadaceae | Pseudoalteromonas | NA |
| ASV_7 | Bacteria | Proteobacteria | Gammaproteobacteria | Alteromonadales | Pseudoalteromonadaceae | Pseudoalteromonas | NA |
| ASV_177 | Bacteria | Proteobacteria | Gammaproteobacteria | Oceanospirillales | Halomonadaceae | Cobetia | NA |
| ASV_14 | Bacteria | Proteobacteria | Gammaproteobacteria | Oceanospirillales | Oceanospirillaceae | Marinomonas | NA |
| ASV_19 | Bacteria | Proteobacteria | Gammaproteobacteria | Oceanospirillales | Oceanospirillaceae | Marinomonas | NA |
| ASV_21 | Bacteria | Proteobacteria | Gammaproteobacteria | Vibrionales | Vibrionaceae | Vibrio | NA |

**Table S2- Comparison between different diversity metrics and models for explaining community function measurements**

| **Diversity Metric** | **Model** | **Measurement** | **AIC** | **R^2^** |
| --- | --- | --- | --- | --- |
| SR | Hyperbolic | Cell Density | 3971.87 | 0.38 |
| SR | Linear | Cell Density | 3974.00 | 0.36 |
| SR | Log Linear | Cell Density | 3970.11 | 0.39 |
| MPD | Hyperbolic | Cell Density | 4006.16 | 0.16 |
| MPD | Linear | Cell Density | 3999.64 | 0.21 |
| MPD | Log Linear | Cell Density | 3997.31 | 0.22 |
| MNTD | Hyperbolic | Cell Density | 4024.37 | 0.02 |
| MNTD | Linear | Cell Density | 4012.92 | 0.11 |
| MNTD | Log Linear | Cell Density | 4021.29 | 0.04 |
| SR | Hyperbolic | Protein/Cell | -2307.11 | 0.05 |
| SR | Linear | Protein/Cell | -2321.94 | 0.16 |
| SR | Log Linear | Protein/Cell | -2317.03 | 0.13 |
| MPD | Hyperbolic | Protein/Cell | -2305.45 | 0.04 |
| MPD | Linear | Protein/Cell | -2313.57 | 0.10 |
| MPD | Log Linear | Protein/Cell | -2311.63 | 0.09 |
| MNTD | Hyperbolic | Protein/Cell | -2303.58 | 0.02 |
| MNTD | Linear | Protein/Cell | -2303.32 | 0.02 |
| MNTD | Log Linear | Protein/Cell | -2301.26 | 0.00 |
| SR | Hyperbolic | CO2 | 294.04 | 0.57 |
| SR | Linear | CO2 | 302.05 | 0.54 |
| SR | Log Linear | CO2 | 289.55 | 0.59 |
| MPD | Hyperbolic | CO2 | 367.35 | 0.19 |
| MPD | Linear | CO2 | 340.40 | 0.36 |
| MPD | Log Linear | CO2 | 347.33 | 0.32 |
| MNTD | Hyperbolic | CO2 | 389.42 | 0.00 |
| MNTD | Linear | CO2 | 354.66 | 0.27 |
| MNTD | Log Linear | CO2 | 368.74 | 0.18 |

**SR= species richness at stationary phase**

**MPD= abundance weighted mean pairwise distance**

**MNTD= abundance weighted mean nearest taxon distance**

**Table S3-Taxa associated with community function in all taxonomic richness windows**

| ASV number | RDP Classification | Kendall's Tau | p-value | Taxonomic richness window |
| --- | --- | --- | --- | --- |
| *Cell Density* | | | | |
| ASV_6 | Bacteria;Proteobacteria;Gammaproteobacteria;Alteromonadales;Pseudoalteromonadaceae;Pseudoalteromonas;NA | -0.49 | 3.99E-02 | 3-5 |
| ASV_6 | Bacteria;Proteobacteria;Gammaproteobacteria;Alteromonadales;Pseudoalteromonadaceae;Pseudoalteromonas;NA | -0.35 | 4.13E-02 | 9-17 |
| ASV_6 | Bacteria;Proteobacteria;Gammaproteobacteria;Alteromonadales;Pseudoalteromonadaceae;Pseudoalteromonas;NA | -0.37 | 2.36E-02 | 9-18 |
| ASV_6 | Bacteria;Proteobacteria;Gammaproteobacteria;Alteromonadales;Pseudoalteromonadaceae;Pseudoalteromonas;NA | -0.37 | 5.00E-02 | 10-18 |
| ASV_6 | Bacteria;Proteobacteria;Gammaproteobacteria;Alteromonadales;Pseudoalteromonadaceae;Pseudoalteromonas;NA | -0.45 | 1.43E-03 | 10-19 |
| ASV_6 | Bacteria;Proteobacteria;Gammaproteobacteria;Alteromonadales;Pseudoalteromonadaceae;Pseudoalteromonas;NA | -0.47 | 3.30E-03 | 11-19 |
| ASV_6 | Bacteria;Proteobacteria;Gammaproteobacteria;Alteromonadales;Pseudoalteromonadaceae;Pseudoalteromonas;NA | -0.45 | 2.88E-03 | 11-20 |
| ASV_6 | Bacteria;Proteobacteria;Gammaproteobacteria;Alteromonadales;Pseudoalteromonadaceae;Pseudoalteromonas;NA | -0.50 | 5.87E-03 | 12-19 |
| ASV_6 | Bacteria;Proteobacteria;Gammaproteobacteria;Alteromonadales;Pseudoalteromonadaceae;Pseudoalteromonas;NA | -0.46 | 7.92E-03 | 12-20 |
| ASV_6 | Bacteria;Proteobacteria;Gammaproteobacteria;Alteromonadales;Pseudoalteromonadaceae;Pseudoalteromonas;NA | -0.39 | 2.85E-02 | 12-21 |
| ASV_18 | Bacteria;Bacteroidetes;Flavobacteriia;Flavobacteriales;Flavobacteriaceae;Polaribacter;NA | 0.35 | 3.15E-02 | 12-21 |
| ASV_6 | Bacteria;Proteobacteria;Gammaproteobacteria;Alteromonadales;Pseudoalteromonadaceae;Pseudoalteromonas;NA | -0.53 | 5.70E-03 | 13-19 |
| ASV_6 | Bacteria;Proteobacteria;Gammaproteobacteria;Alteromonadales;Pseudoalteromonadaceae;Pseudoalteromonas;NA | -0.49 | 8.60E-03 | 13-20 |
| ASV_6 | Bacteria;Proteobacteria;Gammaproteobacteria;Alteromonadales;Pseudoalteromonadaceae;Pseudoalteromonas;NA | -0.40 | 3.14E-02 | 13-21 |
| ASV_6 | Bacteria;Proteobacteria;Gammaproteobacteria;Alteromonadales;Pseudoalteromonadaceae;Pseudoalteromonas;NA | -0.39 | 1.37E-02 | 13-22 |
| ASV_18 | Bacteria;Bacteroidetes;Flavobacteriia;Flavobacteriales;Flavobacteriaceae;Polaribacter;NA | 0.38 | 1.37E-02 | 13-22 |
| ASV_6 | Bacteria;Proteobacteria;Gammaproteobacteria;Alteromonadales;Pseudoalteromonadaceae;Pseudoalteromonas;NA | -0.58 | 3.77E-03 | 14-19 |
| ASV_6 | Bacteria;Proteobacteria;Gammaproteobacteria;Alteromonadales;Pseudoalteromonadaceae;Pseudoalteromonas;NA | -0.53 | 6.62E-03 | 14-20 |
| ASV_6 | Bacteria;Proteobacteria;Gammaproteobacteria;Alteromonadales;Pseudoalteromonadaceae;Pseudoalteromonas;NA | -0.43 | 3.09E-02 | 14-21 |
| ASV_6 | Bacteria;Proteobacteria;Gammaproteobacteria;Alteromonadales;Pseudoalteromonadaceae;Pseudoalteromonas;NA | -0.40 | 1.93E-02 | 14-22 |
| ASV_18 | Bacteria;Bacteroidetes;Flavobacteriia;Flavobacteriales;Flavobacteriaceae;Polaribacter;NA | 0.38 | 1.93E-02 | 14-22 |
| ASV_28 | Bacteria;Proteobacteria;Alphaproteobacteria;Rhodobacterales;Rhodobacteraceae;Celeribacter;NA | 0.34 | 3.63E-02 | 14-22 |
| ASV_6 | Bacteria;Proteobacteria;Gammaproteobacteria;Alteromonadales;Pseudoalteromonadaceae;Pseudoalteromonas;NA | -0.36 | 4.56E-02 | 14-23 |
| ASV_17 | Bacteria;Proteobacteria;Gammaproteobacteria;Alteromonadales;Alteromonadaceae;Paraglaciecola;NA | -0.31 | 4.56E-02 | 14-23 |
| ASV_18 | Bacteria;Bacteroidetes;Flavobacteriia;Flavobacteriales;Flavobacteriaceae;Polaribacter;NA | 0.31 | 4.56E-02 | 14-23 |
| ASV_19 | Bacteria;Proteobacteria;Gammaproteobacteria;Oceanospirillales;Oceanospirillaceae;Marinomonas;NA | 0.31 | 4.77E-02 | 14-23 |
| ASV_28 | Bacteria;Proteobacteria;Alphaproteobacteria;Rhodobacterales;Rhodobacteraceae;Celeribacter;NA | 0.32 | 4.56E-02 | 14-23 |
| ASV_6 | Bacteria;Proteobacteria;Gammaproteobacteria;Alteromonadales;Pseudoalteromonadaceae;Pseudoalteromonas;NA | -0.60 | 7.04E-03 | 15-19 |
| ASV_6 | Bacteria;Proteobacteria;Gammaproteobacteria;Alteromonadales;Pseudoalteromonadaceae;Pseudoalteromonas;NA | -0.52 | 1.67E-02 | 15-20 |
| ASV_6 | Bacteria;Proteobacteria;Gammaproteobacteria;Alteromonadales;Pseudoalteromonadaceae;Pseudoalteromonas;NA | -0.64 | 1.40E-02 | 16-19 |
| ASV_6 | Bacteria;Proteobacteria;Gammaproteobacteria;Alteromonadales;Pseudoalteromonadaceae;Pseudoalteromonas;NA | -0.52 | 4.66E-02 | 16-20 |
| ASV_39 | Bacteria;Proteobacteria;Gammaproteobacteria;Alteromonadales;Alteromonadaceae;Aliiglaciecola;NA | -0.45 | 4.04E-02 | 20-28 |
| ASV_39 | Bacteria;Proteobacteria;Gammaproteobacteria;Alteromonadales;Alteromonadaceae;Aliiglaciecola;NA | -0.48 | 1.24E-02 | 20-29 |
| ASV_18 | Bacteria;Bacteroidetes;Flavobacteriia;Flavobacteriales;Flavobacteriaceae;Polaribacter;NA | -0.38 | 4.95E-02 | 23-30 |
| ASV_20 | Bacteria;Proteobacteria;Alphaproteobacteria;Rhodobacterales;Rhodobacteraceae;NA;NA | -0.41 | 4.95E-02 | 23-30 |
| ASV_18 | Bacteria;Bacteroidetes;Flavobacteriia;Flavobacteriales;Flavobacteriaceae;Polaribacter;NA | -0.36 | 4.39E-02 | 23-31 |
| ASV_20 | Bacteria;Proteobacteria;Alphaproteobacteria;Rhodobacterales;Rhodobacteraceae;NA;NA | -0.43 | 1.72E-02 | 23-31 |
| ASV_8 | Bacteria;Proteobacteria;Alphaproteobacteria;Rhodobacterales;Rhodobacteraceae;Loktanella;pontiacus | 0.37 | 2.06E-02 | 23-33 |
| ASV_18 | Bacteria;Bacteroidetes;Flavobacteriia;Flavobacteriales;Flavobacteriaceae;Polaribacter;NA | -0.35 | 2.06E-02 | 23-33 |
| ASV_20 | Bacteria;Proteobacteria;Alphaproteobacteria;Rhodobacterales;Rhodobacteraceae;NA;NA | -0.30 | 4.35E-02 | 23-33 |
| ASV_20 | Bacteria;Proteobacteria;Alphaproteobacteria;Rhodobacterales;Rhodobacteraceae;NA;NA | -0.44 | 2.35E-02 | 24-31 |
| ASV_8 | Bacteria;Proteobacteria;Alphaproteobacteria;Rhodobacterales;Rhodobacteraceae;Loktanella;pontiacus | 0.33 | 4.22E-02 | 24-33 |
| ASV_18 | Bacteria;Bacteroidetes;Flavobacteriia;Flavobacteriales;Flavobacteriaceae;Polaribacter;NA | -0.31 | 4.22E-02 | 24-33 |
| ASV_20 | Bacteria;Proteobacteria;Alphaproteobacteria;Rhodobacterales;Rhodobacteraceae;NA;NA | -0.31 | 4.22E-02 | 24-33 |
| ASV_73 | Bacteria;Proteobacteria;Gammaproteobacteria;Oceanospirillales;Oceanospirillaceae;Marinomonas;NA | 0.42 | 3.25E-02 | 24-33 |
| ASV_8 | Bacteria;Proteobacteria;Alphaproteobacteria;Rhodobacterales;Rhodobacteraceae;Loktanella;pontiacus | 0.34 | 2.87E-02 | 24-34 |
| ASV_18 | Bacteria;Bacteroidetes;Flavobacteriia;Flavobacteriales;Flavobacteriaceae;Polaribacter;NA | -0.32 | 2.87E-02 | 24-34 |
| ASV_20 | Bacteria;Proteobacteria;Alphaproteobacteria;Rhodobacterales;Rhodobacteraceae;NA;NA | -0.32 | 2.87E-02 | 24-34 |
| ASV_20 | Bacteria;Proteobacteria;Alphaproteobacteria;Rhodobacterales;Rhodobacteraceae;NA;NA | -0.53 | 1.22E-02 | 25-30 |
| ASV_20 | Bacteria;Proteobacteria;Alphaproteobacteria;Rhodobacterales;Rhodobacteraceae;NA;NA | -0.53 | 4.29E-03 | 25-31 |
| ASV_20 | Bacteria;Proteobacteria;Alphaproteobacteria;Rhodobacterales;Rhodobacteraceae;NA;NA | -0.38 | 2.53E-02 | 25-34 |
| ASV_20 | Bacteria;Proteobacteria;Alphaproteobacteria;Rhodobacterales;Rhodobacteraceae;NA;NA | -0.39 | 1.09E-02 | 25-35 |
| ASV_20 | Bacteria;Proteobacteria;Alphaproteobacteria;Rhodobacterales;Rhodobacteraceae;NA;NA | -0.59 | 1.45E-02 | 26-30 |
| ASV_20 | Bacteria;Proteobacteria;Alphaproteobacteria;Rhodobacterales;Rhodobacteraceae;NA;NA | -0.61 | 2.64E-03 | 26-31 |
| ASV_8 | Bacteria;Proteobacteria;Alphaproteobacteria;Rhodobacterales;Rhodobacteraceae;Loktanella;pontiacus | 0.36 | 3.67E-02 | 26-33 |
| ASV_18 | Bacteria;Bacteroidetes;Flavobacteriia;Flavobacteriales;Flavobacteriaceae;Polaribacter;NA | -0.36 | 3.60E-02 | 26-33 |
| ASV_20 | Bacteria;Proteobacteria;Alphaproteobacteria;Rhodobacterales;Rhodobacteraceae;NA;NA | -0.43 | 2.26E-02 | 26-33 |
| ASV_73 | Bacteria;Proteobacteria;Gammaproteobacteria;Oceanospirillales;Oceanospirillaceae;Marinomonas;NA | 0.45 | 3.03E-02 | 26-33 |
| ASV_8 | Bacteria;Proteobacteria;Alphaproteobacteria;Rhodobacterales;Rhodobacteraceae;Loktanella;pontiacus | 0.36 | 3.42E-02 | 26-34 |
| ASV_18 | Bacteria;Bacteroidetes;Flavobacteriia;Flavobacteriales;Flavobacteriaceae;Polaribacter;NA | -0.36 | 3.32E-02 | 26-34 |
| ASV_20 | Bacteria;Proteobacteria;Alphaproteobacteria;Rhodobacterales;Rhodobacteraceae;NA;NA | -0.44 | 1.07E-02 | 26-34 |
| ASV_18 | Bacteria;Bacteroidetes;Flavobacteriia;Flavobacteriales;Flavobacteriaceae;Polaribacter;NA | -0.34 | 3.34E-02 | 26-35 |
| ASV_20 | Bacteria;Proteobacteria;Alphaproteobacteria;Rhodobacterales;Rhodobacteraceae;NA;NA | -0.45 | 3.39E-03 | 26-35 |
| ASV_8 | Bacteria;Proteobacteria;Alphaproteobacteria;Rhodobacterales;Rhodobacteraceae;Loktanella;pontiacus | 0.29 | 4.06E-02 | 26-36 |
| ASV_18 | Bacteria;Bacteroidetes;Flavobacteriia;Flavobacteriales;Flavobacteriaceae;Polaribacter;NA | -0.33 | 1.65E-02 | 26-36 |
| ASV_20 | Bacteria;Proteobacteria;Alphaproteobacteria;Rhodobacterales;Rhodobacteraceae;NA;NA | -0.37 | 1.23E-02 | 26-36 |
| ASV_20 | Bacteria;Proteobacteria;Alphaproteobacteria;Rhodobacterales;Rhodobacteraceae;NA;NA | -0.62 | 7.25E-03 | 27-31 |
| ASV_8 | Bacteria;Proteobacteria;Alphaproteobacteria;Rhodobacterales;Rhodobacteraceae;Loktanella;pontiacus | 0.41 | 2.19E-02 | 27-33 |
| ASV_18 | Bacteria;Bacteroidetes;Flavobacteriia;Flavobacteriales;Flavobacteriaceae;Polaribacter;NA | -0.41 | 2.19E-02 | 27-33 |
| ASV_73 | Bacteria;Proteobacteria;Gammaproteobacteria;Oceanospirillales;Oceanospirillaceae;Marinomonas;NA | 0.49 | 2.19E-02 | 27-33 |
| ASV_8 | Bacteria;Proteobacteria;Alphaproteobacteria;Rhodobacterales;Rhodobacteraceae;Loktanella;pontiacus | 0.41 | 1.33E-02 | 27-34 |
| ASV_18 | Bacteria;Bacteroidetes;Flavobacteriia;Flavobacteriales;Flavobacteriaceae;Polaribacter;NA | -0.42 | 1.33E-02 | 27-34 |
| ASV_20 | Bacteria;Proteobacteria;Alphaproteobacteria;Rhodobacterales;Rhodobacteraceae;NA;NA | -0.41 | 1.33E-02 | 27-34 |
| ASV_73 | Bacteria;Proteobacteria;Gammaproteobacteria;Oceanospirillales;Oceanospirillaceae;Marinomonas;NA | 0.46 | 1.33E-02 | 27-34 |
| ASV_8 | Bacteria;Proteobacteria;Alphaproteobacteria;Rhodobacterales;Rhodobacteraceae;Loktanella;pontiacus | 0.33 | 4.87E-02 | 27-35 |
| ASV_18 | Bacteria;Bacteroidetes;Flavobacteriia;Flavobacteriales;Flavobacteriaceae;Polaribacter;NA | -0.38 | 2.09E-02 | 27-35 |
| ASV_20 | Bacteria;Proteobacteria;Alphaproteobacteria;Rhodobacterales;Rhodobacteraceae;NA;NA | -0.43 | 1.48E-02 | 27-35 |
| ASV_8 | Bacteria;Proteobacteria;Alphaproteobacteria;Rhodobacterales;Rhodobacteraceae;Loktanella;pontiacus | 0.31 | 3.87E-02 | 27-36 |
| ASV_18 | Bacteria;Bacteroidetes;Flavobacteriia;Flavobacteriales;Flavobacteriaceae;Polaribacter;NA | -0.36 | 2.01E-02 | 27-36 |
| ASV_8 | Bacteria;Proteobacteria;Alphaproteobacteria;Rhodobacterales;Rhodobacteraceae;Loktanella;pontiacus | 0.30 | 3.76E-02 | 27-37 |
| ASV_18 | Bacteria;Bacteroidetes;Flavobacteriia;Flavobacteriales;Flavobacteriaceae;Polaribacter;NA | -0.37 | 5.38E-03 | 27-37 |
| ASV_20 | Bacteria;Proteobacteria;Alphaproteobacteria;Rhodobacterales;Rhodobacteraceae;NA;NA | -0.69 | 8.11E-03 | 28-31 |
| ASV_18 | Bacteria;Bacteroidetes;Flavobacteriia;Flavobacteriales;Flavobacteriaceae;Polaribacter;NA | -0.47 | 2.44E-02 | 28-33 |
| ASV_20 | Bacteria;Proteobacteria;Alphaproteobacteria;Rhodobacterales;Rhodobacteraceae;NA;NA | -0.44 | 2.44E-02 | 28-33 |
| ASV_73 | Bacteria;Proteobacteria;Gammaproteobacteria;Oceanospirillales;Oceanospirillaceae;Marinomonas;NA | 0.49 | 2.44E-02 | 28-33 |
| ASV_8 | Bacteria;Proteobacteria;Alphaproteobacteria;Rhodobacterales;Rhodobacteraceae;Loktanella;pontiacus | 0.37 | 4.59E-02 | 28-34 |
| ASV_18 | Bacteria;Bacteroidetes;Flavobacteriia;Flavobacteriales;Flavobacteriaceae;Polaribacter;NA | -0.48 | 1.19E-02 | 28-34 |
| ASV_20 | Bacteria;Proteobacteria;Alphaproteobacteria;Rhodobacterales;Rhodobacteraceae;NA;NA | -0.47 | 1.19E-02 | 28-34 |
| ASV_73 | Bacteria;Proteobacteria;Gammaproteobacteria;Oceanospirillales;Oceanospirillaceae;Marinomonas;NA | 0.47 | 2.77E-02 | 28-34 |
| ASV_18 | Bacteria;Bacteroidetes;Flavobacteriia;Flavobacteriales;Flavobacteriaceae;Polaribacter;NA | -0.43 | 1.19E-02 | 28-35 |
| ASV_20 | Bacteria;Proteobacteria;Alphaproteobacteria;Rhodobacterales;Rhodobacteraceae;NA;NA | -0.48 | 8.84E-03 | 28-35 |
| ASV_73 | Bacteria;Proteobacteria;Gammaproteobacteria;Oceanospirillales;Oceanospirillaceae;Marinomonas;NA | 0.42 | 3.90E-02 | 28-35 |
| ASV_18 | Bacteria;Bacteroidetes;Flavobacteriia;Flavobacteriales;Flavobacteriaceae;Polaribacter;NA | -0.40 | 8.22E-03 | 28-36 |
| ASV_18 | Bacteria;Bacteroidetes;Flavobacteriia;Flavobacteriales;Flavobacteriaceae;Polaribacter;NA | -0.43 | 7.66E-04 | 28-37 |
| ASV_36 | Bacteria;Proteobacteria;Gammaproteobacteria;Oceanospirillales;Oceanospirillaceae;Marinomonas;NA | 0.33 | 3.02E-02 | 28-37 |
| ASV_73 | Bacteria;Proteobacteria;Gammaproteobacteria;Oceanospirillales;Oceanospirillaceae;Marinomonas;NA | 0.51 | 7.66E-04 | 28-37 |
| ASV_18 | Bacteria;Bacteroidetes;Flavobacteriia;Flavobacteriales;Flavobacteriaceae;Polaribacter;NA | -0.43 | 5.12E-04 | 28-38 |
| ASV_20 | Bacteria;Proteobacteria;Alphaproteobacteria;Rhodobacterales;Rhodobacteraceae;NA;NA | -0.75 | 8.35E-03 | 29-31 |
| ASV_8 | Bacteria;Proteobacteria;Alphaproteobacteria;Rhodobacterales;Rhodobacteraceae;Loktanella;pontiacus | 0.51 | 4.84E-02 | 29-33 |
| ASV_8 | Bacteria;Proteobacteria;Alphaproteobacteria;Rhodobacterales;Rhodobacteraceae;Loktanella;pontiacus | 0.48 | 2.85E-02 | 29-34 |
| ASV_18 | Bacteria;Bacteroidetes;Flavobacteriia;Flavobacteriales;Flavobacteriaceae;Polaribacter;NA | -0.42 | 3.45E-02 | 29-34 |
| ASV_20 | Bacteria;Proteobacteria;Alphaproteobacteria;Rhodobacterales;Rhodobacteraceae;NA;NA | -0.45 | 2.85E-02 | 29-34 |
| ASV_73 | Bacteria;Proteobacteria;Gammaproteobacteria;Oceanospirillales;Oceanospirillaceae;Marinomonas;NA | 0.47 | 3.45E-02 | 29-34 |
| ASV_8 | Bacteria;Proteobacteria;Alphaproteobacteria;Rhodobacterales;Rhodobacteraceae;Loktanella;pontiacus | 0.38 | 4.74E-02 | 29-35 |
| ASV_18 | Bacteria;Bacteroidetes;Flavobacteriia;Flavobacteriales;Flavobacteriaceae;Polaribacter;NA | -0.38 | 4.74E-02 | 29-35 |
| ASV_20 | Bacteria;Proteobacteria;Alphaproteobacteria;Rhodobacterales;Rhodobacteraceae;NA;NA | -0.48 | 1.83E-02 | 29-35 |
| ASV_8 | Bacteria;Proteobacteria;Alphaproteobacteria;Rhodobacterales;Rhodobacteraceae;Loktanella;pontiacus | 0.37 | 1.48E-02 | 29-36 |
| ASV_12 | Bacteria;Proteobacteria;Alphaproteobacteria;Rhodobacterales;Rhodobacteraceae;Sulfitobacter;dubius | 0.33 | 2.73E-02 | 29-36 |
| ASV_18 | Bacteria;Bacteroidetes;Flavobacteriia;Flavobacteriales;Flavobacteriaceae;Polaribacter;NA | -0.37 | 1.48E-02 | 29-36 |
| ASV_73 | Bacteria;Proteobacteria;Gammaproteobacteria;Oceanospirillales;Oceanospirillaceae;Marinomonas;NA | 0.47 | 1.48E-02 | 29-36 |
| ASV_8 | Bacteria;Proteobacteria;Alphaproteobacteria;Rhodobacterales;Rhodobacteraceae;Loktanella;pontiacus | 0.33 | 2.19E-02 | 29-37 |
| ASV_12 | Bacteria;Proteobacteria;Alphaproteobacteria;Rhodobacterales;Rhodobacteraceae;Sulfitobacter;dubius | 0.28 | 4.07E-02 | 29-37 |
| ASV_18 | Bacteria;Bacteroidetes;Flavobacteriia;Flavobacteriales;Flavobacteriaceae;Polaribacter;NA | -0.41 | 2.36E-03 | 29-37 |
| ASV_36 | Bacteria;Proteobacteria;Gammaproteobacteria;Oceanospirillales;Oceanospirillaceae;Marinomonas;NA | 0.33 | 2.96E-02 | 29-37 |
| ASV_73 | Bacteria;Proteobacteria;Gammaproteobacteria;Oceanospirillales;Oceanospirillaceae;Marinomonas;NA | 0.51 | 2.28E-03 | 29-37 |
| ASV_8 | Bacteria;Proteobacteria;Alphaproteobacteria;Rhodobacterales;Rhodobacteraceae;Loktanella;pontiacus | 0.32 | 1.83E-02 | 29-38 |
| ASV_18 | Bacteria;Bacteroidetes;Flavobacteriia;Flavobacteriales;Flavobacteriaceae;Polaribacter;NA | -0.42 | 9.86E-04 | 29-38 |
| ASV_36 | Bacteria;Proteobacteria;Gammaproteobacteria;Oceanospirillales;Oceanospirillaceae;Marinomonas;NA | 0.30 | 3.69E-02 | 29-38 |
| ASV_73 | Bacteria;Proteobacteria;Gammaproteobacteria;Oceanospirillales;Oceanospirillaceae;Marinomonas;NA | 0.50 | 9.86E-04 | 29-38 |
| ASV_8 | Bacteria;Proteobacteria;Alphaproteobacteria;Rhodobacterales;Rhodobacteraceae;Loktanella;pontiacus | 0.31 | 1.85E-02 | 29-39 |
| ASV_18 | Bacteria;Bacteroidetes;Flavobacteriia;Flavobacteriales;Flavobacteriaceae;Polaribacter;NA | -0.42 | 6.40E-04 | 29-39 |
| ASV_73 | Bacteria;Proteobacteria;Gammaproteobacteria;Oceanospirillales;Oceanospirillaceae;Marinomonas;NA | 0.50 | 6.40E-04 | 29-39 |
| ASV_8 | Bacteria;Proteobacteria;Alphaproteobacteria;Rhodobacterales;Rhodobacteraceae;Loktanella;pontiacus | 0.66 | 4.18E-03 | 30-33 |
| ASV_18 | Bacteria;Bacteroidetes;Flavobacteriia;Flavobacteriales;Flavobacteriaceae;Polaribacter;NA | -0.62 | 4.18E-03 | 30-33 |
| ASV_73 | Bacteria;Proteobacteria;Gammaproteobacteria;Oceanospirillales;Oceanospirillaceae;Marinomonas;NA | 0.52 | 4.95E-02 | 30-33 |
| ASV_7 | Bacteria;Proteobacteria;Gammaproteobacteria;Alteromonadales;Pseudoalteromonadaceae;Pseudoalteromonas;NA | 0.44 | 4.18E-02 | 30-34 |
| ASV_8 | Bacteria;Proteobacteria;Alphaproteobacteria;Rhodobacterales;Rhodobacteraceae;Loktanella;pontiacus | 0.60 | 4.95E-03 | 30-34 |
| ASV_18 | Bacteria;Bacteroidetes;Flavobacteriia;Flavobacteriales;Flavobacteriaceae;Polaribacter;NA | -0.59 | 4.95E-03 | 30-34 |
| ASV_19 | Bacteria;Proteobacteria;Gammaproteobacteria;Oceanospirillales;Oceanospirillaceae;Marinomonas;NA | 0.42 | 4.18E-02 | 30-34 |
| ASV_73 | Bacteria;Proteobacteria;Gammaproteobacteria;Oceanospirillales;Oceanospirillaceae;Marinomonas;NA | 0.47 | 4.18E-02 | 30-34 |
| ASV_8 | Bacteria;Proteobacteria;Alphaproteobacteria;Rhodobacterales;Rhodobacteraceae;Loktanella;pontiacus | 0.45 | 3.40E-02 | 30-35 |
| ASV_18 | Bacteria;Bacteroidetes;Flavobacteriia;Flavobacteriales;Flavobacteriaceae;Polaribacter;NA | -0.52 | 1.21E-02 | 30-35 |
| ASV_8 | Bacteria;Proteobacteria;Alphaproteobacteria;Rhodobacterales;Rhodobacteraceae;Loktanella;pontiacus | 0.41 | 1.08E-02 | 30-36 |
| ASV_18 | Bacteria;Bacteroidetes;Flavobacteriia;Flavobacteriales;Flavobacteriaceae;Polaribacter;NA | -0.46 | 5.84E-03 | 30-36 |
| ASV_73 | Bacteria;Proteobacteria;Gammaproteobacteria;Oceanospirillales;Oceanospirillaceae;Marinomonas;NA | 0.47 | 1.08E-02 | 30-36 |
| ASV_8 | Bacteria;Proteobacteria;Alphaproteobacteria;Rhodobacterales;Rhodobacteraceae;Loktanella;pontiacus | 0.35 | 1.84E-02 | 30-37 |
| ASV_18 | Bacteria;Bacteroidetes;Flavobacteriia;Flavobacteriales;Flavobacteriaceae;Polaribacter;NA | -0.48 | 6.89E-04 | 30-37 |
| ASV_73 | Bacteria;Proteobacteria;Gammaproteobacteria;Oceanospirillales;Oceanospirillaceae;Marinomonas;NA | 0.51 | 1.71E-03 | 30-37 |
| ASV_8 | Bacteria;Proteobacteria;Alphaproteobacteria;Rhodobacterales;Rhodobacteraceae;Loktanella;pontiacus | 0.34 | 1.66E-02 | 30-38 |
| ASV_18 | Bacteria;Bacteroidetes;Flavobacteriia;Flavobacteriales;Flavobacteriaceae;Polaribacter;NA | -0.48 | 2.83E-04 | 30-38 |
| ASV_73 | Bacteria;Proteobacteria;Gammaproteobacteria;Oceanospirillales;Oceanospirillaceae;Marinomonas;NA | 0.51 | 1.03E-03 | 30-38 |
| ASV_8 | Bacteria;Proteobacteria;Alphaproteobacteria;Rhodobacterales;Rhodobacteraceae;Loktanella;pontiacus | 0.32 | 1.77E-02 | 30-39 |
| ASV_18 | Bacteria;Bacteroidetes;Flavobacteriia;Flavobacteriales;Flavobacteriaceae;Polaribacter;NA | -0.48 | 2.08E-04 | 30-39 |
| ASV_73 | Bacteria;Proteobacteria;Gammaproteobacteria;Oceanospirillales;Oceanospirillaceae;Marinomonas;NA | 0.50 | 5.79E-04 | 30-39 |
| ASV_18 | Bacteria;Bacteroidetes;Flavobacteriia;Flavobacteriales;Flavobacteriaceae;Polaribacter;NA | -0.47 | 6.24E-05 | 30-40 |
| ASV_73 | Bacteria;Proteobacteria;Gammaproteobacteria;Oceanospirillales;Oceanospirillaceae;Marinomonas;NA | 0.48 | 5.11E-04 | 30-40 |
| ASV_3 | Bacteria;Proteobacteria;Gammaproteobacteria;Alteromonadales;Alteromonadaceae;Alteromonas;NA | -0.69 | 4.22E-02 | 31-33 |
| ASV_8 | Bacteria;Proteobacteria;Alphaproteobacteria;Rhodobacterales;Rhodobacteraceae;Loktanella;pontiacus | 0.64 | 4.96E-02 | 31-33 |
| ASV_3 | Bacteria;Proteobacteria;Gammaproteobacteria;Alteromonadales;Alteromonadaceae;Alteromonas;NA | -0.52 | 4.72E-02 | 31-34 |
| ASV_8 | Bacteria;Proteobacteria;Alphaproteobacteria;Rhodobacterales;Rhodobacteraceae;Loktanella;pontiacus | 0.60 | 2.63E-02 | 31-34 |
| ASV_18 | Bacteria;Bacteroidetes;Flavobacteriia;Flavobacteriales;Flavobacteriaceae;Polaribacter;NA | -0.58 | 2.63E-02 | 31-34 |
| ASV_19 | Bacteria;Proteobacteria;Gammaproteobacteria;Oceanospirillales;Oceanospirillaceae;Marinomonas;NA | 0.61 | 2.63E-02 | 31-34 |
| ASV_3 | Bacteria;Proteobacteria;Gammaproteobacteria;Alteromonadales;Alteromonadaceae;Alteromonas;NA | -0.44 | 1.57E-02 | 31-36 |
| ASV_8 | Bacteria;Proteobacteria;Alphaproteobacteria;Rhodobacterales;Rhodobacteraceae;Loktanella;pontiacus | 0.40 | 1.88E-02 | 31-36 |
| ASV_18 | Bacteria;Bacteroidetes;Flavobacteriia;Flavobacteriales;Flavobacteriaceae;Polaribacter;NA | -0.48 | 1.10E-02 | 31-36 |
| ASV_73 | Bacteria;Proteobacteria;Gammaproteobacteria;Oceanospirillales;Oceanospirillaceae;Marinomonas;NA | 0.47 | 1.88E-02 | 31-36 |
| ASV_3 | Bacteria;Proteobacteria;Gammaproteobacteria;Alteromonadales;Alteromonadaceae;Alteromonas;NA | -0.36 | 2.55E-02 | 31-37 |
| ASV_8 | Bacteria;Proteobacteria;Alphaproteobacteria;Rhodobacterales;Rhodobacteraceae;Loktanella;pontiacus | 0.32 | 4.35E-02 | 31-37 |
| ASV_18 | Bacteria;Bacteroidetes;Flavobacteriia;Flavobacteriales;Flavobacteriaceae;Polaribacter;NA | -0.52 | 8.83E-04 | 31-37 |
| ASV_36 | Bacteria;Proteobacteria;Gammaproteobacteria;Oceanospirillales;Oceanospirillaceae;Marinomonas;NA | 0.33 | 4.84E-02 | 31-37 |
| ASV_73 | Bacteria;Proteobacteria;Gammaproteobacteria;Oceanospirillales;Oceanospirillaceae;Marinomonas;NA | 0.51 | 4.85E-03 | 31-37 |
| ASV_18 | Bacteria;Bacteroidetes;Flavobacteriia;Flavobacteriales;Flavobacteriaceae;Polaribacter;NA | -0.51 | 4.01E-04 | 31-38 |
| ASV_73 | Bacteria;Proteobacteria;Gammaproteobacteria;Oceanospirillales;Oceanospirillaceae;Marinomonas;NA | 0.51 | 2.78E-03 | 31-38 |
| ASV_18 | Bacteria;Bacteroidetes;Flavobacteriia;Flavobacteriales;Flavobacteriaceae;Polaribacter;NA | -0.51 | 2.64E-04 | 31-39 |
| ASV_73 | Bacteria;Proteobacteria;Gammaproteobacteria;Oceanospirillales;Oceanospirillaceae;Marinomonas;NA | 0.51 | 1.38E-03 | 31-39 |
| ASV_18 | Bacteria;Bacteroidetes;Flavobacteriia;Flavobacteriales;Flavobacteriaceae;Polaribacter;NA | -0.49 | 9.09E-05 | 31-40 |
| ASV_73 | Bacteria;Proteobacteria;Gammaproteobacteria;Oceanospirillales;Oceanospirillaceae;Marinomonas;NA | 0.47 | 1.34E-03 | 31-40 |
| ASV_3 | Bacteria;Proteobacteria;Gammaproteobacteria;Alteromonadales;Alteromonadaceae;Alteromonas;NA | -0.25 | 2.89E-02 | 31-41 |
| ASV_18 | Bacteria;Bacteroidetes;Flavobacteriia;Flavobacteriales;Flavobacteriaceae;Polaribacter;NA | -0.44 | 8.13E-05 | 31-41 |
| ASV_19 | Bacteria;Proteobacteria;Gammaproteobacteria;Oceanospirillales;Oceanospirillaceae;Marinomonas;NA | 0.25 | 2.89E-02 | 31-41 |
| ASV_66 | Bacteria;Proteobacteria;Deltaproteobacteria;Bdellovibrionales;Bacteriovoracaceae;Halobacteriovorax;NA | -0.31 | 2.89E-02 | 31-41 |
| ASV_18 | Bacteria;Bacteroidetes;Flavobacteriia;Flavobacteriales;Flavobacteriaceae;Polaribacter;NA | -0.49 | 5.57E-03 | 33-37 |
| ASV_73 | Bacteria;Proteobacteria;Gammaproteobacteria;Oceanospirillales;Oceanospirillaceae;Marinomonas;NA | 0.51 | 9.69E-03 | 33-37 |
| ASV_18 | Bacteria;Bacteroidetes;Flavobacteriia;Flavobacteriales;Flavobacteriaceae;Polaribacter;NA | -0.50 | 2.00E-03 | 33-38 |
| ASV_73 | Bacteria;Proteobacteria;Gammaproteobacteria;Oceanospirillales;Oceanospirillaceae;Marinomonas;NA | 0.51 | 4.85E-03 | 33-38 |
| ASV_18 | Bacteria;Bacteroidetes;Flavobacteriia;Flavobacteriales;Flavobacteriaceae;Polaribacter;NA | -0.48 | 1.68E-03 | 33-39 |
| ASV_73 | Bacteria;Proteobacteria;Gammaproteobacteria;Oceanospirillales;Oceanospirillaceae;Marinomonas;NA | 0.50 | 2.95E-03 | 33-39 |
| ASV_18 | Bacteria;Bacteroidetes;Flavobacteriia;Flavobacteriales;Flavobacteriaceae;Polaribacter;NA | -0.47 | 5.82E-04 | 33-40 |
| ASV_73 | Bacteria;Proteobacteria;Gammaproteobacteria;Oceanospirillales;Oceanospirillaceae;Marinomonas;NA | 0.47 | 2.46E-03 | 33-40 |
| ASV_18 | Bacteria;Bacteroidetes;Flavobacteriia;Flavobacteriales;Flavobacteriaceae;Polaribacter;NA | -0.42 | 4.77E-04 | 33-41 |
| ASV_66 | Bacteria;Proteobacteria;Deltaproteobacteria;Bdellovibrionales;Bacteriovoracaceae;Halobacteriovorax;NA | -0.35 | 1.66E-02 | 33-41 |
| ASV_73 | Bacteria;Proteobacteria;Gammaproteobacteria;Oceanospirillales;Oceanospirillaceae;Marinomonas;NA | 0.41 | 2.84E-03 | 33-41 |
| ASV_18 | Bacteria;Bacteroidetes;Flavobacteriia;Flavobacteriales;Flavobacteriaceae;Polaribacter;NA | -0.40 | 6.07E-04 | 33-42 |
| ASV_66 | Bacteria;Proteobacteria;Deltaproteobacteria;Bdellovibrionales;Bacteriovoracaceae;Halobacteriovorax;NA | -0.34 | 1.47E-02 | 33-42 |
| ASV_73 | Bacteria;Proteobacteria;Gammaproteobacteria;Oceanospirillales;Oceanospirillaceae;Marinomonas;NA | 0.41 | 1.51E-03 | 33-42 |
| ASV_18 | Bacteria;Bacteroidetes;Flavobacteriia;Flavobacteriales;Flavobacteriaceae;Polaribacter;NA | -0.37 | 3.29E-03 | 34-43 |
| ASV_18 | Bacteria;Bacteroidetes;Flavobacteriia;Flavobacteriales;Flavobacteriaceae;Polaribacter;NA | -0.35 | 9.64E-03 | 35-43 |
| ASV_18 | Bacteria;Bacteroidetes;Flavobacteriia;Flavobacteriales;Flavobacteriaceae;Polaribacter;NA | -0.37 | 1.07E-03 | 35-44 |
| ASV_73 | Bacteria;Proteobacteria;Gammaproteobacteria;Oceanospirillales;Oceanospirillaceae;Marinomonas;NA | 0.58 | 2.50E-02 | 36-39 |
| ASV_18 | Bacteria;Bacteroidetes;Flavobacteriia;Flavobacteriales;Flavobacteriaceae;Polaribacter;NA | -0.37 | 9.43E-03 | 36-43 |
| ASV_18 | Bacteria;Bacteroidetes;Flavobacteriia;Flavobacteriales;Flavobacteriaceae;Polaribacter;NA | -0.38 | 1.03E-03 | 36-44 |
| ASV_18 | Bacteria;Bacteroidetes;Flavobacteriia;Flavobacteriales;Flavobacteriaceae;Polaribacter;NA | -0.37 | 1.34E-03 | 36-45 |
| ASV_19 | Bacteria;Proteobacteria;Gammaproteobacteria;Oceanospirillales;Oceanospirillaceae;Marinomonas;NA | 0.39 | 4.97E-02 | 37-41 |
| ASV_18 | Bacteria;Bacteroidetes;Flavobacteriia;Flavobacteriales;Flavobacteriaceae;Polaribacter;NA | -0.38 | 2.44E-02 | 37-43 |
| ASV_19 | Bacteria;Proteobacteria;Gammaproteobacteria;Oceanospirillales;Oceanospirillaceae;Marinomonas;NA | 0.35 | 2.55E-02 | 37-43 |
| ASV_18 | Bacteria;Bacteroidetes;Flavobacteriia;Flavobacteriales;Flavobacteriaceae;Polaribacter;NA | -0.40 | 3.86E-03 | 37-44 |
| ASV_18 | Bacteria;Bacteroidetes;Flavobacteriia;Flavobacteriales;Flavobacteriaceae;Polaribacter;NA | -0.37 | 3.76E-03 | 37-45 |
| ASV_18 | Bacteria;Bacteroidetes;Flavobacteriia;Flavobacteriales;Flavobacteriaceae;Polaribacter;NA | -0.36 | 3.74E-03 | 37-46 |
| ASV_18 | Bacteria;Bacteroidetes;Flavobacteriia;Flavobacteriales;Flavobacteriaceae;Polaribacter;NA | -0.35 | 3.60E-02 | 38-44 |
| ASV_18 | Bacteria;Bacteroidetes;Flavobacteriia;Flavobacteriales;Flavobacteriaceae;Polaribacter;NA | -0.33 | 2.95E-02 | 38-45 |
| ASV_18 | Bacteria;Bacteroidetes;Flavobacteriia;Flavobacteriales;Flavobacteriaceae;Polaribacter;NA | -0.31 | 3.48E-02 | 38-46 |
| ASV_18 | Bacteria;Bacteroidetes;Flavobacteriia;Flavobacteriales;Flavobacteriaceae;Polaribacter;NA | -0.29 | 3.71E-02 | 38-47 |
| ASV_19 | Bacteria;Proteobacteria;Gammaproteobacteria;Oceanospirillales;Oceanospirillaceae;Marinomonas;NA | 0.52 | 2.95E-02 | 39-41 |
| ASV_19 | Bacteria;Proteobacteria;Gammaproteobacteria;Oceanospirillales;Oceanospirillaceae;Marinomonas;NA | 0.46 | 3.33E-02 | 39-42 |
| ASV_19 | Bacteria;Proteobacteria;Gammaproteobacteria;Oceanospirillales;Oceanospirillaceae;Marinomonas;NA | 0.42 | 4.74E-02 | 39-43 |
| ASV_78 | Bacteria;Proteobacteria;Gammaproteobacteria;Oceanospirillales;Oceanospirillaceae;Marinomonas;NA | 0.54 | 4.50E-02 | 44-46 |
| *Protein* | | | | |
| ASV_9 | Bacteria;Proteobacteria;Alphaproteobacteria;Rhodobacterales;Rhodobacteraceae;Sulfitobacter;NA | 0.40 | 4.92E-02 | 11-17 |
| ASV_9 | Bacteria;Proteobacteria;Alphaproteobacteria;Rhodobacterales;Rhodobacteraceae;Sulfitobacter;NA | 0.40 | 4.93E-02 | 11-18 |
| ASV_9 | Bacteria;Proteobacteria;Alphaproteobacteria;Rhodobacterales;Rhodobacteraceae;Sulfitobacter;NA | 0.37 | 2.31E-02 | 11-20 |
| ASV_39 | Bacteria;Proteobacteria;Gammaproteobacteria;Alteromonadales;Alteromonadaceae;Aliiglaciecola;NA | -0.45 | 4.33E-02 | 18-25 |
| ASV_39 | Bacteria;Proteobacteria;Gammaproteobacteria;Alteromonadales;Alteromonadaceae;Aliiglaciecola;NA | -0.44 | 4.09E-02 | 18-26 |
| ASV_39 | Bacteria;Proteobacteria;Gammaproteobacteria;Alteromonadales;Alteromonadaceae;Aliiglaciecola;NA | -0.42 | 4.25E-02 | 18-27 |
| ASV_39 | Bacteria;Proteobacteria;Gammaproteobacteria;Alteromonadales;Alteromonadaceae;Aliiglaciecola;NA | -0.44 | 4.78E-02 | 19-26 |
| ASV_39 | Bacteria;Proteobacteria;Gammaproteobacteria;Alteromonadales;Alteromonadaceae;Aliiglaciecola;NA | -0.42 | 4.90E-02 | 19-27 |
| ASV_39 | Bacteria;Proteobacteria;Gammaproteobacteria;Alteromonadales;Alteromonadaceae;Aliiglaciecola;NA | -0.40 | 4.34E-02 | 19-28 |
| ASV_39 | Bacteria;Proteobacteria;Gammaproteobacteria;Alteromonadales;Alteromonadaceae;Aliiglaciecola;NA | -0.59 | 1.27E-02 | 20-25 |
| ASV_39 | Bacteria;Proteobacteria;Gammaproteobacteria;Alteromonadales;Alteromonadaceae;Aliiglaciecola;NA | -0.55 | 1.42E-02 | 20-26 |
| ASV_39 | Bacteria;Proteobacteria;Gammaproteobacteria;Alteromonadales;Alteromonadaceae;Aliiglaciecola;NA | -0.53 | 1.07E-02 | 20-27 |
| ASV_39 | Bacteria;Proteobacteria;Gammaproteobacteria;Alteromonadales;Alteromonadaceae;Aliiglaciecola;NA | -0.51 | 7.89E-03 | 20-28 |
| ASV_13 | Bacteria;Bacteroidetes;Flavobacteriia;Flavobacteriales;Flavobacteriaceae;Algibacter;NA | 0.37 | 3.61E-02 | 20-29 |
| ASV_39 | Bacteria;Proteobacteria;Gammaproteobacteria;Alteromonadales;Alteromonadaceae;Aliiglaciecola;NA | -0.52 | 3.60E-03 | 20-29 |
| ASV_12 | Bacteria;Proteobacteria;Alphaproteobacteria;Rhodobacterales;Rhodobacteraceae;Sulfitobacter;dubius | 0.43 | 4.10E-02 | 23-31 |
| ASV_12 | Bacteria;Proteobacteria;Alphaproteobacteria;Rhodobacterales;Rhodobacteraceae;Sulfitobacter;dubius | 0.42 | 9.93E-03 | 23-33 |
| ASV_12 | Bacteria;Proteobacteria;Alphaproteobacteria;Rhodobacterales;Rhodobacteraceae;Sulfitobacter;dubius | 0.41 | 1.91E-02 | 24-33 |
| ASV_12 | Bacteria;Proteobacteria;Alphaproteobacteria;Rhodobacterales;Rhodobacteraceae;Sulfitobacter;dubius | 0.37 | 3.30E-02 | 24-34 |
| ASV_12 | Bacteria;Proteobacteria;Alphaproteobacteria;Rhodobacterales;Rhodobacteraceae;Sulfitobacter;dubius | 0.43 | 1.68E-02 | 25-33 |
| ASV_12 | Bacteria;Proteobacteria;Alphaproteobacteria;Rhodobacterales;Rhodobacteraceae;Sulfitobacter;dubius | 0.39 | 3.02E-02 | 25-34 |
| ASV_12 | Bacteria;Proteobacteria;Alphaproteobacteria;Rhodobacterales;Rhodobacteraceae;Sulfitobacter;dubius | 0.43 | 5.55E-03 | 25-35 |
| ASV_12 | Bacteria;Proteobacteria;Alphaproteobacteria;Rhodobacterales;Rhodobacteraceae;Sulfitobacter;dubius | 0.40 | 2.01E-02 | 26-35 |
| ASV_18 | Bacteria;Bacteroidetes;Flavobacteriia;Flavobacteriales;Flavobacteriaceae;Polaribacter;NA | -0.31 | 3.23E-02 | 33-41 |
| ASV_18 | Bacteria;Bacteroidetes;Flavobacteriia;Flavobacteriales;Flavobacteriaceae;Polaribacter;NA | -0.28 | 4.99E-02 | 33-42 |
| ASV_19 | Bacteria;Proteobacteria;Gammaproteobacteria;Oceanospirillales;Oceanospirillaceae;Marinomonas;NA | 0.26 | 4.99E-02 | 33-42 |
| ASV_18 | Bacteria;Bacteroidetes;Flavobacteriia;Flavobacteriales;Flavobacteriaceae;Polaribacter;NA | -0.29 | 1.89E-02 | 36-44 |
| ASV_19 | Bacteria;Proteobacteria;Gammaproteobacteria;Oceanospirillales;Oceanospirillaceae;Marinomonas;NA | 0.29 | 1.89E-02 | 36-44 |
| ASV_18 | Bacteria;Bacteroidetes;Flavobacteriia;Flavobacteriales;Flavobacteriaceae;Polaribacter;NA | -0.30 | 1.93E-02 | 36-45 |
| ASV_19 | Bacteria;Proteobacteria;Gammaproteobacteria;Oceanospirillales;Oceanospirillaceae;Marinomonas;NA | 0.25 | 4.70E-02 | 36-45 |
| ASV_18 | Bacteria;Bacteroidetes;Flavobacteriia;Flavobacteriales;Flavobacteriaceae;Polaribacter;NA | -0.34 | 2.54E-02 | 37-44 |
| ASV_19 | Bacteria;Proteobacteria;Gammaproteobacteria;Oceanospirillales;Oceanospirillaceae;Marinomonas;NA | 0.31 | 3.04E-02 | 37-44 |
| ASV_18 | Bacteria;Bacteroidetes;Flavobacteriia;Flavobacteriales;Flavobacteriaceae;Polaribacter;NA | -0.34 | 1.07E-02 | 37-45 |
| ASV_18 | Bacteria;Bacteroidetes;Flavobacteriia;Flavobacteriales;Flavobacteriaceae;Polaribacter;NA | -0.31 | 2.16E-02 | 37-46 |
| ASV_19 | Bacteria;Proteobacteria;Gammaproteobacteria;Oceanospirillales;Oceanospirillaceae;Marinomonas;NA | 0.26 | 4.38E-02 | 37-46 |
| ASV_18 | Bacteria;Bacteroidetes;Flavobacteriia;Flavobacteriales;Flavobacteriaceae;Polaribacter;NA | -0.32 | 4.18E-02 | 38-45 |
| ASV_18 | Bacteria;Bacteroidetes;Flavobacteriia;Flavobacteriales;Flavobacteriaceae;Polaribacter;NA | -0.31 | 4.05E-02 | 39-44 |
| ASV_19 | Bacteria;Proteobacteria;Gammaproteobacteria;Oceanospirillales;Oceanospirillaceae;Marinomonas;NA | 0.31 | 4.05E-02 | 39-44 |
| ASV_30 | Bacteria;Bacteroidetes;Flavobacteriia;Flavobacteriales;Flavobacteriaceae;Wenyingzhuangia;NA | 0.37 | 3.34E-02 | 39-44 |
| ASV_18 | Bacteria;Bacteroidetes;Flavobacteriia;Flavobacteriales;Flavobacteriaceae;Polaribacter;NA | -0.32 | 2.78E-02 | 39-45 |
| ASV_30 | Bacteria;Bacteroidetes;Flavobacteriia;Flavobacteriales;Flavobacteriaceae;Wenyingzhuangia;NA | 0.38 | 1.20E-02 | 39-45 |
| ASV_7 | Bacteria;Proteobacteria;Gammaproteobacteria;Alteromonadales;Pseudoalteromonadaceae;Pseudoalteromonas;NA | 0.45 | 4.01E-02 | 40-43 |
| ASV_30 | Bacteria;Bacteroidetes;Flavobacteriia;Flavobacteriales;Flavobacteriaceae;Wenyingzhuangia;NA | 0.37 | 4.21E-02 | 40-44 |
| ASV_18 | Bacteria;Bacteroidetes;Flavobacteriia;Flavobacteriales;Flavobacteriaceae;Polaribacter;NA | -0.34 | 2.19E-02 | 40-45 |
| ASV_30 | Bacteria;Bacteroidetes;Flavobacteriia;Flavobacteriales;Flavobacteriaceae;Wenyingzhuangia;NA | 0.38 | 1.60E-02 | 40-45 |
| ASV_18 | Bacteria;Bacteroidetes;Flavobacteriia;Flavobacteriales;Flavobacteriaceae;Polaribacter;NA | -0.31 | 1.11E-02 | 40-49 |
| ASV_19 | Bacteria;Proteobacteria;Gammaproteobacteria;Oceanospirillales;Oceanospirillaceae;Marinomonas;NA | 0.29 | 1.11E-02 | 40-49 |
| ASV_7 | Bacteria;Proteobacteria;Gammaproteobacteria;Alteromonadales;Pseudoalteromonadaceae;Pseudoalteromonas;NA | 0.43 | 2.43E-02 | 41-44 |
| ASV_18 | Bacteria;Bacteroidetes;Flavobacteriia;Flavobacteriales;Flavobacteriaceae;Polaribacter;NA | -0.41 | 2.43E-02 | 41-44 |
| ASV_18 | Bacteria;Bacteroidetes;Flavobacteriia;Flavobacteriales;Flavobacteriaceae;Polaribacter;NA | -0.41 | 1.50E-02 | 41-45 |
| ASV_30 | Bacteria;Bacteroidetes;Flavobacteriia;Flavobacteriales;Flavobacteriaceae;Wenyingzhuangia;NA | 0.34 | 4.51E-02 | 41-45 |
| ASV_18 | Bacteria;Bacteroidetes;Flavobacteriia;Flavobacteriales;Flavobacteriaceae;Polaribacter;NA | -0.36 | 2.60E-02 | 41-46 |
| ASV_30 | Bacteria;Bacteroidetes;Flavobacteriia;Flavobacteriales;Flavobacteriaceae;Wenyingzhuangia;NA | 0.32 | 3.86E-02 | 41-46 |
| ASV_18 | Bacteria;Bacteroidetes;Flavobacteriia;Flavobacteriales;Flavobacteriaceae;Polaribacter;NA | -0.37 | 1.12E-02 | 41-47 |
| ASV_18 | Bacteria;Bacteroidetes;Flavobacteriia;Flavobacteriales;Flavobacteriaceae;Polaribacter;NA | -0.33 | 2.81E-02 | 41-48 |
| ASV_18 | Bacteria;Bacteroidetes;Flavobacteriia;Flavobacteriales;Flavobacteriaceae;Polaribacter;NA | -0.36 | 2.84E-03 | 41-49 |
| ASV_19 | Bacteria;Proteobacteria;Gammaproteobacteria;Oceanospirillales;Oceanospirillaceae;Marinomonas;NA | 0.29 | 1.56E-02 | 41-49 |
| ASV_18 | Bacteria;Bacteroidetes;Flavobacteriia;Flavobacteriales;Flavobacteriaceae;Polaribacter;NA | -0.36 | 2.31E-03 | 41-50 |
| ASV_19 | Bacteria;Proteobacteria;Gammaproteobacteria;Oceanospirillales;Oceanospirillaceae;Marinomonas;NA | 0.31 | 8.87E-03 | 41-50 |
| ASV_18 | Bacteria;Bacteroidetes;Flavobacteriia;Flavobacteriales;Flavobacteriaceae;Polaribacter;NA | -0.32 | 1.54E-02 | 42-49 |
| ASV_19 | Bacteria;Proteobacteria;Gammaproteobacteria;Oceanospirillales;Oceanospirillaceae;Marinomonas;NA | 0.36 | 1.13E-02 | 42-49 |
| ASV_18 | Bacteria;Bacteroidetes;Flavobacteriia;Flavobacteriales;Flavobacteriaceae;Polaribacter;NA | -0.32 | 1.24E-02 | 42-50 |
| ASV_19 | Bacteria;Proteobacteria;Gammaproteobacteria;Oceanospirillales;Oceanospirillaceae;Marinomonas;NA | 0.38 | 5.13E-03 | 42-50 |
| ASV_19 | Bacteria;Proteobacteria;Gammaproteobacteria;Oceanospirillales;Oceanospirillaceae;Marinomonas;NA | 0.32 | 1.60E-02 | 42-51 |
| ASV_7 | Bacteria;Proteobacteria;Gammaproteobacteria;Alteromonadales;Pseudoalteromonadaceae;Pseudoalteromonas;NA | 0.64 | 2.55E-02 | 43-44 |
| ASV_18 | Bacteria;Bacteroidetes;Flavobacteriia;Flavobacteriales;Flavobacteriaceae;Polaribacter;NA | -0.52 | 3.71E-02 | 43-45 |
| ASV_18 | Bacteria;Bacteroidetes;Flavobacteriia;Flavobacteriales;Flavobacteriaceae;Polaribacter;NA | -0.37 | 1.06E-02 | 43-49 |
| ASV_19 | Bacteria;Proteobacteria;Gammaproteobacteria;Oceanospirillales;Oceanospirillaceae;Marinomonas;NA | 0.36 | 1.06E-02 | 43-49 |
| ASV_18 | Bacteria;Bacteroidetes;Flavobacteriia;Flavobacteriales;Flavobacteriaceae;Polaribacter;NA | -0.37 | 5.22E-03 | 43-50 |
| ASV_19 | Bacteria;Proteobacteria;Gammaproteobacteria;Oceanospirillales;Oceanospirillaceae;Marinomonas;NA | 0.37 | 5.22E-03 | 43-50 |
| ASV_18 | Bacteria;Bacteroidetes;Flavobacteriia;Flavobacteriales;Flavobacteriaceae;Polaribacter;NA | -0.31 | 1.68E-02 | 43-51 |
| ASV_19 | Bacteria;Proteobacteria;Gammaproteobacteria;Oceanospirillales;Oceanospirillaceae;Marinomonas;NA | 0.32 | 1.68E-02 | 43-51 |
| ASV_18 | Bacteria;Bacteroidetes;Flavobacteriia;Flavobacteriales;Flavobacteriaceae;Polaribacter;NA | -0.31 | 1.70E-02 | 43-53 |
| ASV_19 | Bacteria;Proteobacteria;Gammaproteobacteria;Oceanospirillales;Oceanospirillaceae;Marinomonas;NA | 0.32 | 1.70E-02 | 43-53 |
| ASV_19 | Bacteria;Proteobacteria;Gammaproteobacteria;Oceanospirillales;Oceanospirillaceae;Marinomonas;NA | 0.33 | 4.50E-02 | 44-50 |
| ASV_19 | Bacteria;Proteobacteria;Gammaproteobacteria;Oceanospirillales;Oceanospirillaceae;Marinomonas;NA | 0.38 | 2.31E-02 | 46-56 |
| ASV_19 | Bacteria;Proteobacteria;Gammaproteobacteria;Oceanospirillales;Oceanospirillaceae;Marinomonas;NA | 0.52 | 2.32E-02 | 47-50 |
| ASV_19 | Bacteria;Proteobacteria;Gammaproteobacteria;Oceanospirillales;Oceanospirillaceae;Marinomonas;NA | 0.43 | 1.49E-02 | 47-56 |
| ASV_19 | Bacteria;Proteobacteria;Gammaproteobacteria;Oceanospirillales;Oceanospirillaceae;Marinomonas;NA | 0.43 | 1.01E-02 | 47-57 |
| ASV_19 | Bacteria;Proteobacteria;Gammaproteobacteria;Oceanospirillales;Oceanospirillaceae;Marinomonas;NA | 0.40 | 4.16E-02 | 48-57 |
| ASV_19 | Bacteria;Proteobacteria;Gammaproteobacteria;Oceanospirillales;Oceanospirillaceae;Marinomonas;NA | 0.43 | 1.77E-02 | 48-58 |
| ASV_19 | Bacteria;Proteobacteria;Gammaproteobacteria;Oceanospirillales;Oceanospirillaceae;Marinomonas;NA | 0.43 | 3.05E-02 | 49-58 |
| ASV_19 | Bacteria;Proteobacteria;Gammaproteobacteria;Oceanospirillales;Oceanospirillaceae;Marinomonas;NA | 0.42 | 2.30E-02 | 49-59 |
| ASV_18 | Bacteria;Bacteroidetes;Flavobacteriia;Flavobacteriales;Flavobacteriaceae;Polaribacter;NA | -0.78 | 1.21E-02 | 53-63 |
| ASV_19 | Bacteria;Proteobacteria;Gammaproteobacteria;Oceanospirillales;Oceanospirillaceae;Marinomonas;NA | 0.67 | 3.17E-02 | 53-63 |
| ASV_30 | Bacteria;Bacteroidetes;Flavobacteriia;Flavobacteriales;Flavobacteriaceae;Wenyingzhuangia;NA | -0.61 | 4.12E-02 | 53-63 |
| ASV_18 | Bacteria;Bacteroidetes;Flavobacteriia;Flavobacteriales;Flavobacteriaceae;Polaribacter;NA | -0.64 | 3.33E-02 | 53-66 |
| ASV_19 | Bacteria;Proteobacteria;Gammaproteobacteria;Oceanospirillales;Oceanospirillaceae;Marinomonas;NA | 0.60 | 3.33E-02 | 53-66 |
| ASV_18 | Bacteria;Bacteroidetes;Flavobacteriia;Flavobacteriales;Flavobacteriaceae;Polaribacter;NA | -0.56 | 4.96E-02 | 53-77 |
| ASV_19 | Bacteria;Proteobacteria;Gammaproteobacteria;Oceanospirillales;Oceanospirillaceae;Marinomonas;NA | 0.60 | 4.96E-02 | 53-77 |
| ASV_18 | Bacteria;Bacteroidetes;Flavobacteriia;Flavobacteriales;Flavobacteriaceae;Polaribacter;NA | -0.67 | 2.53E-02 | 54-66 |
| ASV_19 | Bacteria;Proteobacteria;Gammaproteobacteria;Oceanospirillales;Oceanospirillaceae;Marinomonas;NA | 0.67 | 2.53E-02 | 54-66 |
| ASV_19 | Bacteria;Proteobacteria;Gammaproteobacteria;Oceanospirillales;Oceanospirillaceae;Marinomonas;NA | 0.69 | 2.81E-02 | 54-77 |
| ASV_18 | Bacteria;Bacteroidetes;Flavobacteriia;Flavobacteriales;Flavobacteriaceae;Polaribacter;NA | -0.67 | 3.17E-02 | 55-77 |
| ASV_19 | Bacteria;Proteobacteria;Gammaproteobacteria;Oceanospirillales;Oceanospirillaceae;Marinomonas;NA | 0.72 | 2.94E-02 | 55-77 |
| *CO2* | | | | |
| ASV_3 | Bacteria;Proteobacteria;Gammaproteobacteria;Alteromonadales;Alteromonadaceae;Alteromonas;NA | 0.36 | 1.16E-02 | 2-7 |
| ASV_3 | Bacteria;Proteobacteria;Gammaproteobacteria;Alteromonadales;Alteromonadaceae;Alteromonas;NA | 0.34 | 8.29E-03 | 2-8 |
| ASV_3 | Bacteria;Proteobacteria;Gammaproteobacteria;Alteromonadales;Alteromonadaceae;Alteromonas;NA | 0.34 | 5.40E-03 | 2-9 |
| ASV_3 | Bacteria;Proteobacteria;Gammaproteobacteria;Alteromonadales;Alteromonadaceae;Alteromonas;NA | 0.30 | 1.49E-02 | 2-10 |
| ASV_3 | Bacteria;Proteobacteria;Gammaproteobacteria;Alteromonadales;Alteromonadaceae;Alteromonas;NA | 0.28 | 2.14E-02 | 2-11 |
| ASV_3 | Bacteria;Proteobacteria;Gammaproteobacteria;Alteromonadales;Alteromonadaceae;Alteromonas;NA | 0.47 | 3.03E-02 | 3-5 |
| ASV_3 | Bacteria;Proteobacteria;Gammaproteobacteria;Alteromonadales;Alteromonadaceae;Alteromonas;NA | 0.45 | 1.38E-02 | 3-6 |
| ASV_3 | Bacteria;Proteobacteria;Gammaproteobacteria;Alteromonadales;Alteromonadaceae;Alteromonas;NA | 0.44 | 2.75E-03 | 3-7 |
| ASV_3 | Bacteria;Proteobacteria;Gammaproteobacteria;Alteromonadales;Alteromonadaceae;Alteromonas;NA | 0.40 | 2.41E-03 | 3-8 |
| ASV_3 | Bacteria;Proteobacteria;Gammaproteobacteria;Alteromonadales;Alteromonadaceae;Alteromonas;NA | 0.39 | 1.45E-03 | 3-9 |
| ASV_3 | Bacteria;Proteobacteria;Gammaproteobacteria;Alteromonadales;Alteromonadaceae;Alteromonas;NA | 0.34 | 5.36E-03 | 3-10 |
| ASV_3 | Bacteria;Proteobacteria;Gammaproteobacteria;Alteromonadales;Alteromonadaceae;Alteromonas;NA | 0.31 | 9.50E-03 | 3-11 |
| ASV_3 | Bacteria;Proteobacteria;Gammaproteobacteria;Alteromonadales;Alteromonadaceae;Alteromonas;NA | 0.30 | 8.69E-03 | 3-12 |
| ASV_3 | Bacteria;Proteobacteria;Gammaproteobacteria;Alteromonadales;Alteromonadaceae;Alteromonas;NA | 0.37 | 4.47E-02 | 4-7 |
| ASV_3 | Bacteria;Proteobacteria;Gammaproteobacteria;Alteromonadales;Alteromonadaceae;Alteromonas;NA | 0.34 | 3.22E-02 | 4-8 |
| ASV_3 | Bacteria;Proteobacteria;Gammaproteobacteria;Alteromonadales;Alteromonadaceae;Alteromonas;NA | 0.34 | 2.12E-02 | 4-9 |
| ASV_3 | Bacteria;Proteobacteria;Gammaproteobacteria;Alteromonadales;Alteromonadaceae;Alteromonas;NA | 0.41 | 4.90E-02 | 5-7 |
| ASV_3 | Bacteria;Proteobacteria;Gammaproteobacteria;Alteromonadales;Alteromonadaceae;Alteromonas;NA | 0.38 | 2.55E-02 | 5-8 |
| ASV_3 | Bacteria;Proteobacteria;Gammaproteobacteria;Alteromonadales;Alteromonadaceae;Alteromonas;NA | 0.38 | 1.43E-02 | 5-9 |
| ASV_28 | Bacteria;Proteobacteria;Alphaproteobacteria;Rhodobacterales;Rhodobacteraceae;Celeribacter;NA | 0.39 | 1.97E-02 | 10-19 |
| ASV_6 | Bacteria;Proteobacteria;Gammaproteobacteria;Alteromonadales;Pseudoalteromonadaceae;Pseudoalteromonas;NA | -0.36 | 3.55E-02 | 11-19 |
| ASV_28 | Bacteria;Proteobacteria;Alphaproteobacteria;Rhodobacterales;Rhodobacteraceae;Celeribacter;NA | 0.39 | 3.55E-02 | 11-19 |
| ASV_6 | Bacteria;Proteobacteria;Gammaproteobacteria;Alteromonadales;Pseudoalteromonadaceae;Pseudoalteromonas;NA | -0.36 | 2.33E-02 | 11-20 |
| ASV_28 | Bacteria;Proteobacteria;Alphaproteobacteria;Rhodobacterales;Rhodobacteraceae;Celeribacter;NA | 0.37 | 2.33E-02 | 11-20 |
| ASV_6 | Bacteria;Proteobacteria;Gammaproteobacteria;Alteromonadales;Pseudoalteromonadaceae;Pseudoalteromonas;NA | -0.47 | 2.27E-02 | 13-19 |
| ASV_6 | Bacteria;Proteobacteria;Gammaproteobacteria;Alteromonadales;Pseudoalteromonadaceae;Pseudoalteromonas;NA | -0.43 | 3.16E-02 | 13-20 |
| ASV_6 | Bacteria;Proteobacteria;Gammaproteobacteria;Alteromonadales;Pseudoalteromonadaceae;Pseudoalteromonas;NA | -0.49 | 3.06E-02 | 14-19 |
| ASV_6 | Bacteria;Proteobacteria;Gammaproteobacteria;Alteromonadales;Pseudoalteromonadaceae;Pseudoalteromonas;NA | -0.51 | 4.07E-02 | 15-19 |
| ASV_12 | Bacteria;Proteobacteria;Alphaproteobacteria;Rhodobacterales;Rhodobacteraceae;Sulfitobacter;dubius | 0.41 | 3.93E-02 | 20-27 |
| ASV_19 | Bacteria;Proteobacteria;Gammaproteobacteria;Oceanospirillales;Oceanospirillaceae;Marinomonas;NA | 0.43 | 3.93E-02 | 20-27 |
| ASV_39 | Bacteria;Proteobacteria;Gammaproteobacteria;Alteromonadales;Alteromonadaceae;Aliiglaciecola;NA | -0.43 | 3.93E-02 | 20-27 |
| ASV_19 | Bacteria;Proteobacteria;Gammaproteobacteria;Oceanospirillales;Oceanospirillaceae;Marinomonas;NA | 0.40 | 3.69E-02 | 20-28 |
| ASV_39 | Bacteria;Proteobacteria;Gammaproteobacteria;Alteromonadales;Alteromonadaceae;Aliiglaciecola;NA | -0.42 | 3.69E-02 | 20-28 |
| ASV_19 | Bacteria;Proteobacteria;Gammaproteobacteria;Oceanospirillales;Oceanospirillaceae;Marinomonas;NA | 0.34 | 4.94E-02 | 20-29 |
| ASV_39 | Bacteria;Proteobacteria;Gammaproteobacteria;Alteromonadales;Alteromonadaceae;Aliiglaciecola;NA | -0.40 | 4.94E-02 | 20-29 |
| ASV_19 | Bacteria;Proteobacteria;Gammaproteobacteria;Oceanospirillales;Oceanospirillaceae;Marinomonas;NA | 0.63 | 7.86E-03 | 22-27 |
| ASV_19 | Bacteria;Proteobacteria;Gammaproteobacteria;Oceanospirillales;Oceanospirillaceae;Marinomonas;NA | 0.56 | 1.05E-02 | 22-28 |
| ASV_19 | Bacteria;Proteobacteria;Gammaproteobacteria;Oceanospirillales;Oceanospirillaceae;Marinomonas;NA | 0.46 | 3.49E-02 | 22-29 |
| ASV_19 | Bacteria;Proteobacteria;Gammaproteobacteria;Oceanospirillales;Oceanospirillaceae;Marinomonas;NA | 0.45 | 1.66E-02 | 22-30 |
| ASV_20 | Bacteria;Proteobacteria;Alphaproteobacteria;Rhodobacterales;Rhodobacteraceae;NA;NA | -0.38 | 3.74E-02 | 22-30 |
| ASV_19 | Bacteria;Proteobacteria;Gammaproteobacteria;Oceanospirillales;Oceanospirillaceae;Marinomonas;NA | 0.47 | 5.07E-03 | 22-31 |
| ASV_20 | Bacteria;Proteobacteria;Alphaproteobacteria;Rhodobacterales;Rhodobacteraceae;NA;NA | -0.37 | 3.25E-02 | 22-31 |
| ASV_19 | Bacteria;Proteobacteria;Gammaproteobacteria;Oceanospirillales;Oceanospirillaceae;Marinomonas;NA | 0.68 | 4.34E-02 | 23-26 |
| ASV_19 | Bacteria;Proteobacteria;Gammaproteobacteria;Oceanospirillales;Oceanospirillaceae;Marinomonas;NA | 0.72 | 5.36E-03 | 23-27 |
| ASV_20 | Bacteria;Proteobacteria;Alphaproteobacteria;Rhodobacterales;Rhodobacteraceae;NA;NA | -0.54 | 3.71E-02 | 23-27 |
| ASV_19 | Bacteria;Proteobacteria;Gammaproteobacteria;Oceanospirillales;Oceanospirillaceae;Marinomonas;NA | 0.61 | 1.07E-02 | 23-28 |
| ASV_20 | Bacteria;Proteobacteria;Alphaproteobacteria;Rhodobacterales;Rhodobacteraceae;NA;NA | -0.56 | 1.07E-02 | 23-28 |
| ASV_19 | Bacteria;Proteobacteria;Gammaproteobacteria;Oceanospirillales;Oceanospirillaceae;Marinomonas;NA | 0.48 | 2.13E-02 | 23-29 |
| ASV_20 | Bacteria;Proteobacteria;Alphaproteobacteria;Rhodobacterales;Rhodobacteraceae;NA;NA | -0.50 | 1.97E-02 | 23-29 |
| ASV_19 | Bacteria;Proteobacteria;Gammaproteobacteria;Oceanospirillales;Oceanospirillaceae;Marinomonas;NA | 0.48 | 6.71E-03 | 23-30 |
| ASV_20 | Bacteria;Proteobacteria;Alphaproteobacteria;Rhodobacterales;Rhodobacteraceae;NA;NA | -0.47 | 6.71E-03 | 23-30 |
| ASV_19 | Bacteria;Proteobacteria;Gammaproteobacteria;Oceanospirillales;Oceanospirillaceae;Marinomonas;NA | 0.50 | 3.80E-03 | 23-31 |
| ASV_20 | Bacteria;Proteobacteria;Alphaproteobacteria;Rhodobacterales;Rhodobacteraceae;NA;NA | -0.45 | 5.65E-03 | 23-31 |
| ASV_19 | Bacteria;Proteobacteria;Gammaproteobacteria;Oceanospirillales;Oceanospirillaceae;Marinomonas;NA | 0.44 | 3.44E-03 | 23-33 |
| ASV_19 | Bacteria;Proteobacteria;Gammaproteobacteria;Oceanospirillales;Oceanospirillaceae;Marinomonas;NA | 0.70 | 1.65E-02 | 24-27 |
| ASV_19 | Bacteria;Proteobacteria;Gammaproteobacteria;Oceanospirillales;Oceanospirillaceae;Marinomonas;NA | 0.58 | 2.67E-02 | 24-28 |
| ASV_20 | Bacteria;Proteobacteria;Alphaproteobacteria;Rhodobacterales;Rhodobacteraceae;NA;NA | -0.54 | 2.67E-02 | 24-28 |
| ASV_20 | Bacteria;Proteobacteria;Alphaproteobacteria;Rhodobacterales;Rhodobacteraceae;NA;NA | -0.53 | 2.34E-02 | 24-29 |
| ASV_19 | Bacteria;Proteobacteria;Gammaproteobacteria;Oceanospirillales;Oceanospirillaceae;Marinomonas;NA | 0.43 | 3.16E-02 | 24-30 |
| ASV_20 | Bacteria;Proteobacteria;Alphaproteobacteria;Rhodobacterales;Rhodobacteraceae;NA;NA | -0.49 | 1.57E-02 | 24-30 |
| ASV_19 | Bacteria;Proteobacteria;Gammaproteobacteria;Oceanospirillales;Oceanospirillaceae;Marinomonas;NA | 0.45 | 1.01E-02 | 24-31 |
| ASV_20 | Bacteria;Proteobacteria;Alphaproteobacteria;Rhodobacterales;Rhodobacteraceae;NA;NA | -0.47 | 1.01E-02 | 24-31 |
| ASV_19 | Bacteria;Proteobacteria;Gammaproteobacteria;Oceanospirillales;Oceanospirillaceae;Marinomonas;NA | 0.39 | 2.26E-02 | 24-33 |
| ASV_19 | Bacteria;Proteobacteria;Gammaproteobacteria;Oceanospirillales;Oceanospirillaceae;Marinomonas;NA | 0.41 | 7.61E-03 | 24-34 |
| ASV_20 | Bacteria;Proteobacteria;Alphaproteobacteria;Rhodobacterales;Rhodobacteraceae;NA;NA | -0.32 | 4.31E-02 | 24-34 |
| ASV_20 | Bacteria;Proteobacteria;Alphaproteobacteria;Rhodobacterales;Rhodobacteraceae;NA;NA | -0.47 | 4.18E-02 | 25-30 |
| ASV_19 | Bacteria;Proteobacteria;Gammaproteobacteria;Oceanospirillales;Oceanospirillaceae;Marinomonas;NA | 0.44 | 1.89E-02 | 25-31 |
| ASV_20 | Bacteria;Proteobacteria;Alphaproteobacteria;Rhodobacterales;Rhodobacteraceae;NA;NA | -0.45 | 1.89E-02 | 25-31 |
| ASV_19 | Bacteria;Proteobacteria;Gammaproteobacteria;Oceanospirillales;Oceanospirillaceae;Marinomonas;NA | 0.38 | 4.33E-02 | 25-33 |
| ASV_19 | Bacteria;Proteobacteria;Gammaproteobacteria;Oceanospirillales;Oceanospirillaceae;Marinomonas;NA | 0.40 | 1.36E-02 | 25-34 |
| ASV_19 | Bacteria;Proteobacteria;Gammaproteobacteria;Oceanospirillales;Oceanospirillaceae;Marinomonas;NA | 0.38 | 1.73E-02 | 25-35 |
| ASV_8 | Bacteria;Proteobacteria;Alphaproteobacteria;Rhodobacterales;Rhodobacteraceae;Loktanella;pontiacus | 0.30 | 4.93E-02 | 26-36 |
| ASV_18 | Bacteria;Bacteroidetes;Flavobacteriia;Flavobacteriales;Flavobacteriaceae;Polaribacter;NA | -0.30 | 4.93E-02 | 26-36 |
| ASV_20 | Bacteria;Proteobacteria;Alphaproteobacteria;Rhodobacterales;Rhodobacteraceae;NA;NA | -0.53 | 4.02E-02 | 27-31 |
| ASV_19 | Bacteria;Proteobacteria;Gammaproteobacteria;Oceanospirillales;Oceanospirillaceae;Marinomonas;NA | 0.37 | 4.58E-02 | 27-35 |
| ASV_20 | Bacteria;Proteobacteria;Alphaproteobacteria;Rhodobacterales;Rhodobacteraceae;NA;NA | -0.35 | 4.58E-02 | 27-35 |
| ASV_8 | Bacteria;Proteobacteria;Alphaproteobacteria;Rhodobacterales;Rhodobacteraceae;Loktanella;pontiacus | 0.32 | 4.90E-02 | 28-36 |
| ASV_18 | Bacteria;Bacteroidetes;Flavobacteriia;Flavobacteriales;Flavobacteriaceae;Polaribacter;NA | -0.31 | 4.90E-02 | 28-36 |
| ASV_8 | Bacteria;Proteobacteria;Alphaproteobacteria;Rhodobacterales;Rhodobacteraceae;Loktanella;pontiacus | 0.31 | 4.90E-02 | 28-38 |
| ASV_8 | Bacteria;Proteobacteria;Alphaproteobacteria;Rhodobacterales;Rhodobacteraceae;Loktanella;pontiacus | 0.37 | 2.98E-02 | 29-36 |
| ASV_18 | Bacteria;Bacteroidetes;Flavobacteriia;Flavobacteriales;Flavobacteriaceae;Polaribacter;NA | -0.35 | 2.98E-02 | 29-36 |
| ASV_8 | Bacteria;Proteobacteria;Alphaproteobacteria;Rhodobacterales;Rhodobacteraceae;Loktanella;pontiacus | 0.35 | 3.16E-02 | 29-37 |
| ASV_18 | Bacteria;Bacteroidetes;Flavobacteriia;Flavobacteriales;Flavobacteriaceae;Polaribacter;NA | -0.33 | 3.16E-02 | 29-37 |
| ASV_8 | Bacteria;Proteobacteria;Alphaproteobacteria;Rhodobacterales;Rhodobacteraceae;Loktanella;pontiacus | 0.35 | 2.59E-02 | 29-38 |
| ASV_18 | Bacteria;Bacteroidetes;Flavobacteriia;Flavobacteriales;Flavobacteriaceae;Polaribacter;NA | -0.30 | 3.96E-02 | 29-38 |
| ASV_8 | Bacteria;Proteobacteria;Alphaproteobacteria;Rhodobacterales;Rhodobacteraceae;Loktanella;pontiacus | 0.36 | 1.33E-02 | 29-39 |
| ASV_18 | Bacteria;Bacteroidetes;Flavobacteriia;Flavobacteriales;Flavobacteriaceae;Polaribacter;NA | -0.30 | 3.88E-02 | 29-39 |
| ASV_8 | Bacteria;Proteobacteria;Alphaproteobacteria;Rhodobacterales;Rhodobacteraceae;Loktanella;pontiacus | 0.54 | 4.13E-02 | 30-33 |
| ASV_18 | Bacteria;Bacteroidetes;Flavobacteriia;Flavobacteriales;Flavobacteriaceae;Polaribacter;NA | -0.50 | 4.13E-02 | 30-33 |
| ASV_8 | Bacteria;Proteobacteria;Alphaproteobacteria;Rhodobacterales;Rhodobacteraceae;Loktanella;pontiacus | 0.51 | 3.44E-02 | 30-34 |
| ASV_18 | Bacteria;Bacteroidetes;Flavobacteriia;Flavobacteriales;Flavobacteriaceae;Polaribacter;NA | -0.47 | 3.44E-02 | 30-34 |
| ASV_19 | Bacteria;Proteobacteria;Gammaproteobacteria;Oceanospirillales;Oceanospirillaceae;Marinomonas;NA | 0.46 | 3.44E-02 | 30-34 |
| ASV_8 | Bacteria;Proteobacteria;Alphaproteobacteria;Rhodobacterales;Rhodobacteraceae;Loktanella;pontiacus | 0.45 | 3.40E-02 | 30-35 |
| ASV_18 | Bacteria;Bacteroidetes;Flavobacteriia;Flavobacteriales;Flavobacteriaceae;Polaribacter;NA | -0.47 | 3.29E-02 | 30-35 |
| ASV_19 | Bacteria;Proteobacteria;Gammaproteobacteria;Oceanospirillales;Oceanospirillaceae;Marinomonas;NA | 0.42 | 3.79E-02 | 30-35 |
| ASV_8 | Bacteria;Proteobacteria;Alphaproteobacteria;Rhodobacterales;Rhodobacteraceae;Loktanella;pontiacus | 0.42 | 1.23E-02 | 30-36 |
| ASV_18 | Bacteria;Bacteroidetes;Flavobacteriia;Flavobacteriales;Flavobacteriaceae;Polaribacter;NA | -0.45 | 8.21E-03 | 30-36 |
| ASV_8 | Bacteria;Proteobacteria;Alphaproteobacteria;Rhodobacterales;Rhodobacteraceae;Loktanella;pontiacus | 0.38 | 1.17E-02 | 30-37 |
| ASV_18 | Bacteria;Bacteroidetes;Flavobacteriia;Flavobacteriales;Flavobacteriaceae;Polaribacter;NA | -0.39 | 1.17E-02 | 30-37 |
| ASV_8 | Bacteria;Proteobacteria;Alphaproteobacteria;Rhodobacterales;Rhodobacteraceae;Loktanella;pontiacus | 0.37 | 1.16E-02 | 30-38 |
| ASV_18 | Bacteria;Bacteroidetes;Flavobacteriia;Flavobacteriales;Flavobacteriaceae;Polaribacter;NA | -0.36 | 1.16E-02 | 30-38 |
| ASV_8 | Bacteria;Proteobacteria;Alphaproteobacteria;Rhodobacterales;Rhodobacteraceae;Loktanella;pontiacus | 0.39 | 8.74E-03 | 30-39 |
| ASV_18 | Bacteria;Bacteroidetes;Flavobacteriia;Flavobacteriales;Flavobacteriaceae;Polaribacter;NA | -0.34 | 1.33E-02 | 30-39 |
| ASV_8 | Bacteria;Proteobacteria;Alphaproteobacteria;Rhodobacterales;Rhodobacteraceae;Loktanella;pontiacus | 0.31 | 1.81E-02 | 30-40 |
| ASV_18 | Bacteria;Bacteroidetes;Flavobacteriia;Flavobacteriales;Flavobacteriaceae;Polaribacter;NA | -0.36 | 6.05E-03 | 30-40 |
| ASV_19 | Bacteria;Proteobacteria;Gammaproteobacteria;Oceanospirillales;Oceanospirillaceae;Marinomonas;NA | 0.61 | 4.84E-02 | 31-34 |
| ASV_18 | Bacteria;Bacteroidetes;Flavobacteriia;Flavobacteriales;Flavobacteriaceae;Polaribacter;NA | -0.50 | 3.72E-02 | 31-35 |
| ASV_19 | Bacteria;Proteobacteria;Gammaproteobacteria;Oceanospirillales;Oceanospirillaceae;Marinomonas;NA | 0.52 | 3.72E-02 | 31-35 |
| ASV_18 | Bacteria;Bacteroidetes;Flavobacteriia;Flavobacteriales;Flavobacteriaceae;Polaribacter;NA | -0.51 | 5.53E-03 | 31-36 |
| ASV_18 | Bacteria;Bacteroidetes;Flavobacteriia;Flavobacteriales;Flavobacteriaceae;Polaribacter;NA | -0.43 | 1.47E-02 | 31-37 |
| ASV_18 | Bacteria;Bacteroidetes;Flavobacteriia;Flavobacteriales;Flavobacteriaceae;Polaribacter;NA | -0.38 | 2.79E-02 | 31-38 |
| ASV_8 | Bacteria;Proteobacteria;Alphaproteobacteria;Rhodobacterales;Rhodobacteraceae;Loktanella;pontiacus | 0.35 | 2.12E-02 | 31-39 |
| ASV_18 | Bacteria;Bacteroidetes;Flavobacteriia;Flavobacteriales;Flavobacteriaceae;Polaribacter;NA | -0.35 | 2.12E-02 | 31-39 |
| ASV_18 | Bacteria;Bacteroidetes;Flavobacteriia;Flavobacteriales;Flavobacteriaceae;Polaribacter;NA | -0.39 | 5.16E-03 | 31-40 |
| ASV_18 | Bacteria;Bacteroidetes;Flavobacteriia;Flavobacteriales;Flavobacteriaceae;Polaribacter;NA | -0.31 | 1.88E-02 | 31-41 |
| ASV_18 | Bacteria;Bacteroidetes;Flavobacteriia;Flavobacteriales;Flavobacteriaceae;Polaribacter;NA | -0.46 | 4.14E-02 | 33-36 |
| ASV_18 | Bacteria;Bacteroidetes;Flavobacteriia;Flavobacteriales;Flavobacteriaceae;Polaribacter;NA | -0.36 | 2.59E-02 | 33-40 |
| ASV_18 | Bacteria;Bacteroidetes;Flavobacteriia;Flavobacteriales;Flavobacteriaceae;Polaribacter;NA | -0.29 | 4.05E-02 | 33-42 |
| ASV_66 | Bacteria;Proteobacteria;Deltaproteobacteria;Bdellovibrionales;Bacteriovoracaceae;Halobacteriovorax;NA | -0.31 | 4.05E-02 | 33-42 |
| ASV_8 | Bacteria;Proteobacteria;Alphaproteobacteria;Rhodobacterales;Rhodobacteraceae;Loktanella;pontiacus | 0.43 | 3.05E-02 | 34-39 |
| ASV_8 | Bacteria;Proteobacteria;Alphaproteobacteria;Rhodobacterales;Rhodobacteraceae;Loktanella;pontiacus | 0.58 | 6.86E-03 | 36-39 |
| ASV_19 | Bacteria;Proteobacteria;Gammaproteobacteria;Oceanospirillales;Oceanospirillaceae;Marinomonas;NA | -0.45 | 4.55E-02 | 36-39 |
| ASV_3 | Bacteria;Proteobacteria;Gammaproteobacteria;Alteromonadales;Alteromonadaceae;Alteromonas;NA | -0.48 | 4.30E-02 | 40-42 |
| ASV_3 | Bacteria;Proteobacteria;Gammaproteobacteria;Alteromonadales;Alteromonadaceae;Alteromonas;NA | -0.45 | 3.32E-02 | 40-43 |
| ASV_3 | Bacteria;Proteobacteria;Gammaproteobacteria;Alteromonadales;Alteromonadaceae;Alteromonas;NA | -0.52 | 4.72E-02 | 41-43 |
| ASV_18 | Bacteria;Bacteroidetes;Flavobacteriia;Flavobacteriales;Flavobacteriaceae;Polaribacter;NA | -0.54 | 3.31E-02 | 42-44 |

**Table S4- ANOVA of community function**
